# Overview of miR-106a Regulatory Roles: from Cancer to Aging

**DOI:** 10.3390/bioengineering10080892

**Published:** 2023-07-27

**Authors:** Maryam Daneshpour, Ali Ghadimi-Daresajini

**Affiliations:** 1 Biotechnology Department, School of Advanced Technologies in Medicine, Shahid Beheshti University of Medical Sciences, Tehran 1985717443, Iran; 2 Department of Medical Biotechnology, School of Allied Medicine, Cellular and Molecular Research Center, Iran University of Medical Sciences, Tehran 1449614535, Iran; ghadimi.a@tak.iums.ac.ir

**Keywords:** miR-106a, microRNA, cancer, biomarker, drug resistance

## Abstract

MicroRNAs (miRNAs) comprise a class of non-coding RNA with extensive regulatory functions within cells. MiR-106a is recognized for its super-regulatory roles in vital processes. Hence, the analysis of its expression in association with diseases has attracted considerable attention for molecular diagnosis and drug development. Numerous studies have investigated miR-106 target genes and shown that this miRNA regulates the expression of some critical cell cycle and apoptosis factors, suggesting miR-106a as an ideal diagnostic and prognostic biomarker with therapeutic potential. Furthermore, the reported correlation between miR-106a expression level and cancer drug resistance has demonstrated the complexity of its functions within different tissues. In this study, we have conducted a comprehensive review on the expression levels of miR-106a in various cancers and other diseases, emphasizing its target genes. The promising findings surrounding miR-106a suggest its potential as a valuable biomolecule. However, further validation assessments and overcoming existing limitations are crucial steps before its clinical implementation can be realized.

## 1. Introduction

MicroRNAs (miRNAs) are small, endogenous, non-coding RNAs that control gene expression at the translation and even transcription levels. miRNAs are critical regulators of biological processes, including cellular proliferation, differentiation, development, apoptosis, and modulation of the host response to viral infection [1,2]. Moreover, extracellular miRNAs have been widely reported as potential biomarkers for different diseases and disorders while also serving as signaling molecules to mediate cell–cell communications [3,4].

The biogenesis process of these tiny biomolecules generally begins with RNA polymerase II/III activity and can be classified into canonical and noncanonical pathways. While the intron and exon parts of the genome are involved in processing intragenic miRNAs, intergenic miRNAs are believed to be transcribed independently of genes and regulated by their own promoters [5]. miRNAs may be transcribed in the form of long transcripts named clusters. If the clusters share similar seed regions, they are considered to be an miRNA family [4,5].

In this review, we focus on miR-106a, a multi-faceted miRNA, and its regulating role in vital cell processes [6]. miR-106a is a member of the miR-17 family and a part of the miR-106a-363 cluster located on the X chromosome [7]. As listed in Table S1 in Supplementary Materials, there are 1337 predicted targets for hsa-miR-106a-5p in miRDB, with target scores ranging from 100 to 50 (higher miRDB scores generally indicate a stronger likelihood of a functional miRNA-mRNA interaction, suggesting a higher probability that the miRNA can regulate the expression of the targeted mRNA) [8,9]. Considering that the majority of identified target genes are implicated in crucial processes such as cell cycle regulation, drug resistance, tumor progression, and the inflammatory response, it is expected that the dysregulation of miR-106a is associated with a wide range of biological phenomena, diseases, disorders, and malignancies (Figure S1 in Supplementary Materials). In addition, miR-106a regulates several genes involved in cell signaling pathways. However, confirmation of some of these genes still needs further clarification and verification [2,10].

## 2. mir-106a: Dysregulation in Cancers

### 2.1. Colorectal Cancer

Colorectal cancer (CRC) is the third most common cancer with the second-highest global mortality rate [11]. Despite progress in its diagnosis and treatment, the overall 5-year survival rate is 40%, and approximately half of the patients die due to the development of distant metastases [12].

More than 200 miRNAs have been found to be dysregulated in CRC, according to the literature and the miRCancer website [12,13]. A significant number of studies have reported the overexpression of miR-106a in plasma, cancer tissues, and stool samples of CRC patients via real-time RT-PCR and microarray analyses [14,15,16,17]; however, the precise role played by miR-106a in CRC is not completely clear. In a study by Feng et al., the authors studied 990 targets of miR-106a predicted by TargetScan and indicated that transforming growth factor-β receptor 2 (TGFBR2) is the direct functional target of miR-106a. As a member of the primary TGF-β signaling molecules, TGFBR2 expression is frequently altered in cancer cell lines through induction of EMT and promotion of tumor cell invasion. The results of this study showed that miR-106a was positively correlated with the migration and invasion potential of CRC cells by downregulating TGFBR2 [2,18,19]. In another study, Hao et al. investigated the correlation between miR-106a and autophagy-related gene 7 (ATG7) in CRC. The obtained results confirmed that miR-106a downregulates ATG7 mRNA, leading to the suppression of tumor cell death [14].

**Figure 1:**
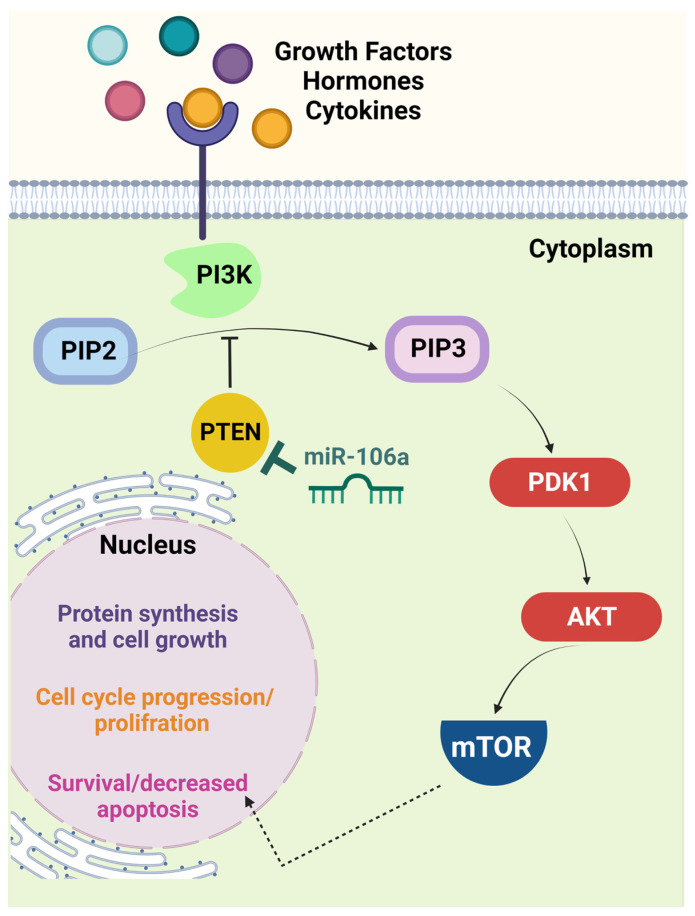
The PTEN/PI3K/AKT signaling pathway is a crucial target of miR-106a. In the presence of specific growth factors, hormones, or cytokines, interaction with the cell surface receptor activates the receptor complex and PI3K. PI3K then phosphorylates PIP2 to PIP3, which recruits PDK1 to activate AKT. Activated AKT interacts with different molecules, including mTOR. The latter molecule induces cell proliferation and limits apoptosis. PTEN is one of the principal negative regulators of this pathway via reversing the conversion of PIP2 to PIP3. This function makes PTEN a critical tumor suppressor, the expression of which is diminished in many malignancies via different mechanisms, including miR-106a-mediated downregulation. Thus, loss of PTEN correlates with uncontrolled cell cycle progression and probable tumorigenesis [25]. (Created with BioRender.com, accessed on 14 December 2022). AKT, serine/threonine protein kinase B; mTOR, mammalian target of rapamycin; PDK1, phosphoinositide-dependent protein kinase 1; PI3K, phosphatidylinositol 3-kinase; PIP2, phosphatidylinositol diphosphate; PIP3, phosphatidylinositol trisphosphate; PTEN, phosphatase and tensin homolog deleted on chromosome 10.

Phosphatase and tensin homolog deleted on chromosome ten (PTEN) is a tumor suppressor downregulated by miR-106a overexpression in CRC. The PTEN/PI3K/AKT signaling pathway (Figure 1) was identified as another target for miR-106a in CRC in research published by Qin et al. [20]. Evidence indicates that the increased activity of PI3K/AKT following PTEN reduction correlates with cell proliferation, migration, invasion, and apoptosis [21,22]. In addition, the inactivation of PTEN results in malignant progression, including advanced stage in CRC [23].

Despite studies emphasizing the overexpression of miR-106a in CRC, Huang and Ma performed an in vitro experiment using cultured CRC cells. Their results showed the tumor-suppressive function of miR-106a in CRC potentially works through regulating E2F1 and caspase-9 expression [24].

### 2.2. Cholangiocarcinoma

Cholangiocarcinoma (CCA) is a malignant tumor originating from the bile duct epithelium with frequent metastasis to the lymph node [26]. However, since its pathogenesis mechanism and provoking factors are still unclear, patients often reach the advanced stages when diagnosed with CCA. For CCA patients, radical resection is almost the only choice, but certainly not a viable one [26,27].

In interesting research by Cheng et al., a pooled analysis was performed to identify a clinically valid miRNA in CCA patients. Their results indicated that the circulating level of miR-106a was significantly lower in CCA patients with a higher susceptibility to lymph node metastasis [26]. However, these findings were not corroborated at the tissue level when assessing miR-106a expression. This paradox may stem from the potential influence of the tumor microenvironment [26]. The origin of circulating miRNAs has been the subject of several studies and remains a topic of debate, and researchers have discussed that serum miRNA levels may not only result from tumors but also from the immune response [26].

Regarding the current data obtained from several studies, the diagnostic value of miR-106a for CCA patients seems clear. Additionally, miR-106a is a possible prognostic biomarker in CCA patients thanks to presenting as a clinically promising indicator for evaluating lymph node metastasis risk [28].

### 2.3. Ewing Sarcoma

Ewing sarcoma (EWS) is the second most common malignancy related to solid bone and soft tissue in children and young adults [29]. Most EWS tumors harbor t(11,22)(q24:12) chromosomal translocation followed by EWS-FLI1 gene fusion. This fusion acts as an aberrant transcription factor, interacts with RNAs, and dysregulates cellular processes, such as cell cycle progression, and TGF-β signaling [29].

Several studies on the role of miRNAs in EWS have highlighted the upregulation of miR-106a in this malignancy. There is evidence indicating the oncogenic behavior of miR-106a leads to increased expression of Bim (BCL-2 interacting mediator of cell death) and decreased expression of CDK4/CDK6 [29,30,31]. Moreover, the results published by Dylla et al. showed that targeting miR-106a could not effectively inhibit tumor development in responsive EWS cell lines. They suggested that inhibition of the entire miR-106a~363 cluster may be required to obtain significant results [2,30,31].

### 2.4. Gastric Cancer

Gastric cancer (GC) is the fourth leading cancer and third cause of cancer-related deaths worldwide. Although research on gastric cancer has made significant advancements, the molecular mechanisms underlying cancer invasion and metastasis are still poorly understood [32]. Due to a lack of symptoms in the early stages, most patients with GC are diagnosed with advanced disease or distant metastasis. According to research, early detection in gastric cancer patients leads to a better prognosis and a 5-year survival rate of more than 90% [32,33].

Recently, many ectopically expressed miRNAs have been reported to be involved in the initiation and progression of gastric cancer as novel proto-oncogene and tumor-suppressor genes. The expression level of miR-106a increases and can be detected in tumor tissue, blood, fecal, and gastric fluids [34,35]. Moreover, it has been revealed that miR-106a levels are significantly associated with tumor stage, size, and differentiation, lymphatic and distant metastasis, and invasion [33,36]. 

**Figure 2:**
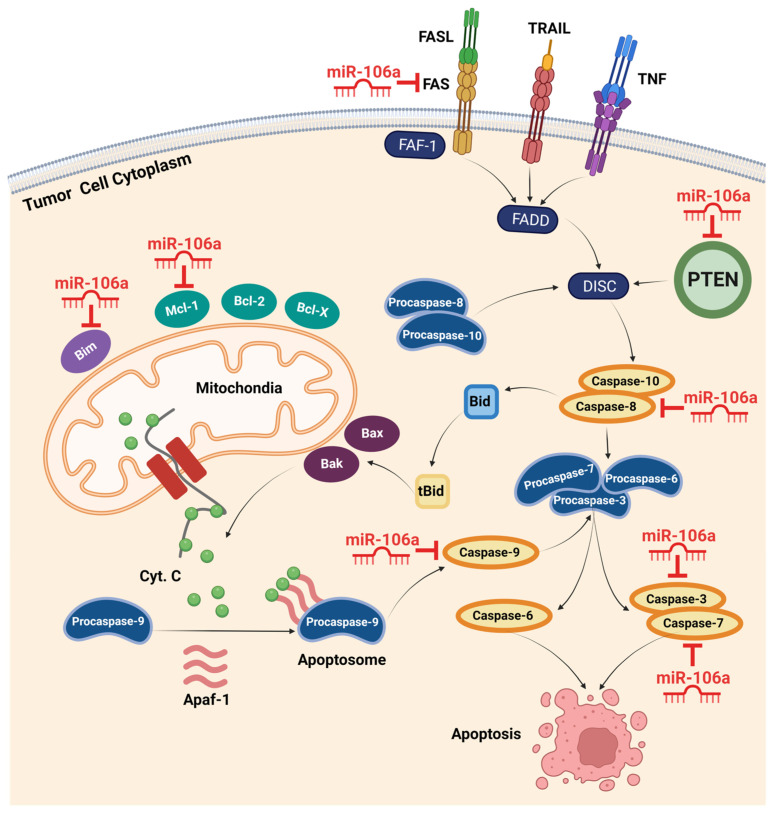
Intrinsic and extrinsic apoptotic pathways and confirmed target genes of miR-106a. The extrinsic pathway is mediated by death ligands and death receptors, including FASL and FAS. Activation of death receptors initiates a series of reactions with final stimulation of caspases 3, 6, and 7, resulting in apoptosis. miR-106a can hinder apoptosis by suppressing multiple members of this pathway, including FAS, PTEN, and caspase-8, -3, and -7. The intrinsic pathway incorporates mitochondria in promoting apoptosis. Activating this pathway in response to different stresses, such as oncogenic activity, stimulates mitochondrial membrane proteins to release cytochrome C into the cytoplasm. Recruiting Apaf-1 and cytochrome C, procaspase-9 forms the apoptosome complex and converts to active caspase-9. By activating other apoptotic caspases, the cell consequently heads to apoptosis. miR-106a overexpression inhibits this pathway via targeting Bim, Mcl-1, and caspase-9 in different cancers. (Created with BioRender.com, accessed on 14 December 2022). Apaf-1, apoptotic peptidase activating factor 1; Bak, Bcl-2-antagonist killer; Bax, Bcl-2-associated x protein; Bcl-2, B-cell lymphoma 2; Bcl-x, B-cell lymphoma-extra-large; Bid, BH3 interacting-domain death agonist; Bim, Bcl-2 interacting mediator of cell death; DISC, death-inducing signaling complex; FADD, FAS-associated death domain; FAF-1, FAS-associated factor 1; FASL, FAS ligand; Mcl-1, myeloid cell leukemia 1; PTEN, phosphatase and tensin homolog deleted on chromosome 10; TNF, tumor necrosis factor; TRAIL, TNF-related apoptosis-inducing ligand.

Zhu et al. used qualitative, quantitative, and positioning analyses to investigate the biological role of miR-106a in GC. The results demonstrated that the upregulation of miR-106a significantly enhanced GC cell proliferation, migration, and invasion by directly targeting TIMP2. TIMP2, a metallopeptidase inhibitor, plays a crucial role in suppressing endothelial cell proliferation [34]. Moreover, in a study by Wang et al., rescue experiments and examination of caspase-8, PARP, and caspase-3 demonstrated that miR-106a could inhibit gastric cancer cell apoptosis by interfering with the FAS-mediated apoptotic pathway [37]. A significant inverse correlation was also found between miR-106a and FAS expression in gastric cancer cell lines and specimens. Therefore, these findings suggest that ectopically overexpressed miR-106a plays an oncogenic role in gastric carcinogenesis by impairing extrinsic apoptotic pathways via targeting FAS [37,38]. The confirmed target genes of miR-106a in intrinsic and extrinsic apoptotic pathways are shown in Figure 2.

Recent research has revealed that certain long noncoding RNAs (lncRNAs) function as competing endogenous RNAs (ceRNAs) to regulate gene expression [39]. These lncRNAs, along with other RNAs, act as miRNA sponges by sharing miRNA response elements (MREs), thereby modulating intracellular miRNA function [32]. Given that noncoding RNAs are predominantly untranslated, they are presumed to be highly effective ceRNAs. One such lncRNA, FER1L4 (long noncoding RNA Fer-1 protein 4), has been shown to play crucial regulatory roles in tumor progression [40]. FER1L4 functions as a ceRNA by sequestering miR-106a-5p in gastric cancer, thereby impacting tumor suppression. Consequently, this ceRNA pathway can ultimately regulate the expression levels of several critical genes, including PTEN, RB1, RUNX1, VEGFA, CDKN1A, E2F1, HIPK3, IL-10, and PAK7, through lncRNA-FER1L4 [40,41].

The development of multidrug resistance (MDR) in gastric cancer poses significant challenges in effective treatment strategies. Dysregulation of key molecular pathways and the involvement of specific microRNAs have been implicated in MDR mechanisms. In this context, the role of miR-106a and its impact on the PTEN/Akt pathway and downstream targets have gained considerable attention.

PTEN, encoding a phosphatase involved in the negative regulation of the Akt pathway by dephosphorylating PIP3 and counteracting PI3K activity, has emerged as one of the main targets of miR-106a, particularly in MDR GC [41,42]. Studies have reported that miR-106a overexpression leads to PTEN knockdown, resulting in dysregulation of the PTEN/Akt pathway. This dysregulation has been associated with the development of MDR in cisplatin-treated GC patients [41,42].

Further investigations have shed light on additional mechanisms through which miR-106a induces MDR in GC patients. It has been observed that miR-106a targets P-glycoprotein (P-gp), an ATP-binding cassette transporter responsible for drug efflux [42]. Moreover, miR-106a overexpression correlates with the downregulation of runt-related transcription factor 3 (RUNX3), a tumor suppressor known to inhibit cell progression and tumorigenesis (Guo et al., 2005). According to Guo et al., RUNX3 prevents the expression of MDR-1, Bcl-2, and multidrug resistance protein-1, while enhancing the sensitivity of GC cells to chemotherapeutic drugs [43]. The dysregulation caused by miR-106a actively promotes MDR in GC cells by desensitizing tumor cells to anticancer drugs, increasing the efflux of chemotherapeutic agents, and suppressing GC cell apoptosis through the dysregulation of tumor suppressor genes [42,43].

### 2.5. Esophageal Carcinoma

Esophageal cancer is among the malignancies with high mortality worldwide [44]. Esophageal cancer is mainly classified as esophageal adenocarcinoma and esophageal squamous cell carcinoma (ESCC). ESCC is responsible for >90% of esophageal cancer and is the most aggressive carcinoma of the gastrointestinal tract [45,46]. 

Since the first study on the levels of serum miRNAs in ESCC patients was published [47], the differential expression of circulating miRNAs and the potential application of biomarkers in ESCC have gained considerable attention in the cancer research field [44,46]. One study analyzed the expression level of miR-106a in the tissues of 21 ESCC patients and reported its reduction in patients that developed recurrent disease or had a tumor-related death [48]. However, because of the small number of examined samples, these findings were not reliable.

In a study conducted by Zhou et al., an miRNA signature consisting of five upregulated miRNAs (miR-106a, miR-18a, miR-20b, miR-486-5p, and miR-584) and one down-regulated miRNA (miR-223-3p) was identified as a potential biomarker for the diagnosis of ESCC. The study utilized PCR assays to demonstrate that the levels of miR-106a and miR-584 were consistently elevated in both the plasma and tissue samples of ESCC patients. These findings suggest that the plasma levels of these two miRNAs hold promise as potential screening biomarkers for ESCC [44]. However, further investigations are necessary to identify the specific target genes or functional pathways associated with miR-106a in this particular cancer in order to establish the clinical validity of the reported results.

### 2.6. Renal Carcinoma

Renal cell carcinoma (RCC) is a highly lethal form of genitourinary cancer, comprising approximately 90% of all renal malignancies, with an annual increase in the incidence of 2%–3% [49]. The major problems of RCC are the unsatisfactory postoperative survival rate and poor responsiveness to radiotherapy and chemotherapy [49].

The regulatory role of miRNAs and their dysregulation in RCC have been investigated in numerous studies and miR-106a has been identified as a tumor suppressor with dramatically decreased levels in the plasma and tissue of RCC patients. It was shown that increased miR-106a levels inhibited the proliferation of RCC cells and prevented S/G2 transition [50].

It was also found that miR-106a expression was inversely correlated with the expression of insulin receptor substrate 2 (IRS-2) [51]. Insulin receptor substrates (IRSs), comprising six members (IRS-1-6) [52,53], are signaling adaptor proteins. IRSs are immediate downstream effectors of insulin-like growth factor-1 (IGF-1), insulin receptors, prolactin, growth hormone, cytokine, VEGF receptors, and integrin receptor family members [50,51]. The results reported by Ma et al. indicated that IRS-2 was the functional and direct target of miR-106a. Therefore, downregulation of miR-106a in RCC activates IRS-2, which is involved in the PI3K/Akt signaling pathway in different cancer model systems. This signaling pathway has been implicated in promoting tumor cell proliferation, migration, invasion, and survival [51].

PAK5 is a serine/threonine kinase downstream of Rho GTPases. Many studies have found that PAK5 plays a crucial role in promoting cell migration and invasion in tumorigenesis [54,55,56]. Due to harboring a highly conserved p21-GTPase-binding domain, PAK5 may easily interact with a Rho GTPase (GTP-binding Cdc42) and other Rho GTPases like Rho and Rac that affect the cytoskeleton, cell dynamics, and cell morphology. In addition, PAK5-mediated signaling pathways such as PAK5-Egr1-MMP2 are involved in tumor cell migration and invasion [54,56]. Pan et al. identified PAK5 as a target of miR-106a, which promotes the malignancy process in RCC via reduction of its miRNA levels [49].

Furthermore, it was reported that miR-106a-5p directly targets the 3′-UTR of VEGFA mRNA and decreases VEGFA expression, followed by degrading effects on tumor cell growth and colony formation [57]. Therefore, the low level of miR-106a in RCC tissues was correlated with the upregulation of VEGFA, which contributes to angiogenesis and tumor development [57,58].

### 2.7. Lung Cancer

Non-small cell lung cancer (NSCLC), which represents about 80% of all lung cancer cases, is a leading cause of cancer death in the developed world. Less than 15% of NSCLC patients live more than five years after diagnosis [59].

To study the principal miRNAs involved in NSCLC, Heegaard et al. examined serum and plasma samples from 220 patients with early-stage NSCLC and 220 matched controls using qRT-PCR. They found that the expression levels of miR-146b, miR-221, let-7a, miR-155, miR-17-5p, miR-27a, and miR-106a were significantly reduced in the serum of NSCLC cases while that of miR-29c was considerably increased. However, no significant differences were observed in the plasma of patients compared to controls, and expression levels in serum and plasma did not correlate well [60].

By studying the miRNAs dysregulated in NSCLC tissues and cell lines, Xie et al. confirmed miR-106a upregulation and identified PTEN as a target for this miRNA. This study suggested that miR-106a inhibited the growth and metastasis of NSCLC cells by decreasing PTEN expression [61]. 

Adenosine triphosphatase-binding cassette A1 (ABCA1) is a member of the ABC transporter family and is fundamental to cellular metabolism. ABCA1 is also one of the main players connected to miR-106a-induced drug resistance in NSCLC. Lee et al. [62] discovered that cancer-specific hypermethylation and downregulated expression of ABCA1 contributed to an elevated level of intracellular cholesterol, followed by enhanced vulnerability to tumor progression. These findings indicated the anticancer role of cholesterol exporter ABCA1 and its impact on tumor development, which were supported by the results of Smith and Land [63]. Their research provided evidence suggesting that the restoration of ABCA1 expression could inhibit tumor development [63]. In this regard, Ma et al. reported that the upregulation of miR-106a expression levels was associated with cisplatin (DDP) resistance by targeting ABCA1. It was also confirmed that the modulation of miR-106a in DDP-treated cells re-sensitized them to DDP [64].

### 2.8. Pancreatic Cancer

Pancreatic cancer (PC) is one of the leading causes of cancer-related mortality due to poor early diagnosis and imprecise prognosis [65]. The lack of warning signs at the initial stages of PC leads to late and commonly inefficient interventions, followed by a low survival rate [65]. Studies, however, indicate that detection of PC before reaching the ultimate stages or metastatic level, followed by surgical resection and chemotherapy, might notably increase the survival rate of patients (Rawat et al., 2019).

The potential of miRNAs as PC biomarkers has been broadly investigated (Vieira et al., 2021; Yan et al., 2020). miRNA dysregulation patterns can be practically evaluated to differentiate PC from other pancreatic diseases, such as pancreatitis and benign pancreatic masses, compared to healthy samples (Vieira et al., 2021; Yan et al., 2020).

Studies investigating the molecular mechanisms underlying the impact of miR-106a in pancreatic cancer have revealed additional key insights [65,66]. It has been observed that miR-106a promotes pancreatic tumorigenesis by directly targeting multiple genes involved in tumor suppression and cellular proliferation pathways [65,66]. For instance, miR-106a was been found to target retinoblastoma protein (RB1), a critical tumor suppressor involved in cell cycle regulation. By inhibiting RB1 expression, miR-106a disrupts cell cycle control and promotes uncontrolled cell growth in pancreatic cancer cells [66]. Moreover, miR-106a overexpression in PC is correlated with pancreatic tumorigenesis by inducing cancer cell proliferation, epithelial–mesenchymal transition, and invasion by targeting tissue inhibitor of metalloproteinase-2 (TIMP-2) [66]. These molecular interactions highlight the intricate role of miR-106a in driving pancreatic cancer progression through the modulation of key regulatory pathways.

### 2.9. Ovarian Cancer

Ovarian cancer (OC) is the most lethal gynecological malignancy commonly diagnosed at an advanced stage with extensive peritoneal metastases due to the absence of warning symptoms and lack of effective screening methods at the early stage [67]. Investigating the miRNAs dysregulated in OC led to the identification of miR-106a as an oncomiR that is overexpressed in tumor tissue samples and cell lines and facilitates cell growth and metastasis by reducing PTEN expression [68]. Chen et al. also indicated that IL-6 is a regulator of miR-106a and inhibits miR-106a expression by activating STAT3 [68]. In a series of experiments using the OncomiR database, western blotting, and luciferase reporter assays, Chao et al. validated Rho GTPase-activating protein 24 (ARHGAP24) as a direct target of miR-106a. The expression of ARHGAP24 protein was suppressed by the overexpression of miR-106a-5p [67].

In a study by Liu et al., miR-106a upregulation was indicated in high-grade serous ovarian carcinomas (HGSOC) based on the real-time reverse transcriptase PCR (qRT-PCR) results and miRNA in situ hybridization in a large cohort study of HGSOC samples. They additionally showed the correlation of miR-106a with HGSOC tumor growth and differentiation both in vivo and in vitro, confirming p130 (RBL2), a retinoblastoma (Rb) tumor suppressor family member, as a specific target of miR-106a [69].

The role of miR-106a in the drug resistance behavior of OC-involved tissue has also been explored, revealing that miR-106a-mediated resistance of ovarian cancer cell line A2780 to the chemotherapeutic agent cisplatin (DDP) was accomplished through targeting Mcl-1 [70]. Reports showed that the Mcl-1 gene was involved in cisplatin and paclitaxel resistance in OC. Mcl-1, a member of the Bcl-2 family, contributes to the ability of cells to survive and evade the toxic effects of the drug [70,71,72].

The association between PDCD4 (programmed cell death 4) protein expression level and miR-106a upregulation in the ovarian cancer OVCAR3 cell line and the cisplatin (DDP)-resistant ovarian cancer OVCAR3/CIS cell line was studied using stem-loop qPCR [73]. PDCD4, a tumor suppressor, reportedly inhibited tumorous transformation, progression, and translation. By interacting with such factors as RNA helicase eIF4A and scaffold protein eIF4G, PDCD4 inhibits protein synthesis by suppressing translation initiation [2]. The results demonstrated that PDCD4 is a target of miR-106a and played a role in DDP resistance in the OVCR3 cell line. Furthermore, the knockdown of PDCD4 significantly increased the cell survival rate and had an overall effect similar to miR-106a overexpression [73].

Moreover, through immunoblotting and luciferase assays, Huh et al. identified BCL10 and caspase-7 as direct target genes of miR-106a downregulation in OC. The authors suggested that the chemoresistance associated with miR-106a in the OC cell line may be induced by direct regulation of BCL10 and/or caspase-7 [71].

Guo et al. conducted investigations on the lncRNA X inactive specific transcript (XIST) and observed its correlation with OC and miR-106a expression [74]. Although the XIST mechanism of action in OC still requires validation and in vivo proof, it is clear that its expression declines as OC progresses. Since the interaction between miR-106a and XIST has been shown using various techniques, such as dual-luciferase reporter and RNA pull-down assays, XIST is considered another target for miR-106a in OC. Further in vitro and in vivo results have also demonstrated the probable therapeutic potential of XIST in restoring tumor cell apoptosis via sponging miR-106a [74].

### 2.10. Brain Tumors

Tumors that originate in the brain are classified as primary brain tumors, which can be either benign or malignant. Approximately half of all primary brain tumors arise from glial cells, collectively named gliomas. The World Health Organization (WHO) has divided gliomas into two categories: low-grade (WHO grades I and II: pilocytic astrocytoma and diffuse astrocytoma, respectively) and high-grade (WHO grades III and IV: anaplastic astrocytoma and glioblastoma multiforms (GBMs), respectively) [75,76].

Other types of brain tumors include choroids plexus tumors, primitive neuroectodermal tumors (PNET, e.g., medulloblastoma, neuroblastoma, retinoblastoma, pineoblastoma), tumors originating from neuronal cells (gangliocytoma, central neurocytoma), mixed glioneuronal tumors (tumors displaying a neuronal as well as a glial component, such as ganglioglioma), and dysembryoplastic neuroepithelial tumors (DNET) [76].

The role of miRNAs and their dysregulation in brain tumors have been extensively investigated in separate studies and miR-106a has an undeniable but unclear effect on brain tumor development and progression [75,76,77] Although different researchers have reported the overexpression of miR-106a in brain tumors [7,77,78,79], there are studies paradoxically indicating the tumor suppression behavior of this miRNA in GMB and astrocytoma [80,81,82,83].

According to published data of glioblastoma cell cultures, it is currently believed that miR-106a promotes tumor cell invasion via activation of the WNT signaling pathway and altering β-catenin cellular localization [78]. Binding β-catenin to the promoter regions of a series of oncogenes, including MMP2, NANOG, MYC, RUNX2, CD44, SOX9, and OCT4, is functionally related to cell invasion [78]. Wang et al. have also explored two functionally related microRNAs, miR-20a and miR-106a, in human glioma stem cells (GSCs) and found that these two molecules were significantly overexpressed in related tissues and enhanced the invasiveness of CD133 + GSCs by directly targeting TIMP-2 [79].

On the other hand, one of the first studies reporting the tumor-suppressive effect of miR-106a in GBM demonstrated that the low expression of miR-106a in human glioma specimens is associated with the accumulation of E2F1 protein and high-grade glioma [81]. The authors identified E2F1 as a direct functional target of miR-106a, suggesting that the effect of miR-106a on the glioma suppressive effect may result from the inhibition of E2F1 via posttranscriptional regulation. In addition, their results revealed that miR-106a could increase p53 expression via E2F1 inhibition, whereas the effect of miR-106a on the proliferation of glioma cells was independent of p53 status [81]. Furthermore, Dai et al. reported the tumor suppressor role of miR-106a and its involvement in GBM cell proliferation and even glucose uptake by targeting SLC2A3. In addition, they showed that the downregulation of miR-106a in GBM tissues leads to poor survival in GBM patients [80].

Significant decreases in miR-106a levels have also been reported in brain tumor types other than GBM. It was revealed that miR-106a targets FASTK in astrocytoma cells and inhibits their proliferation and migration while inducing apoptosis [83]. Jones et al. reported the upregulation of miR-106a in PA and suggested its effect via targeting the MAPK and NF-κB pathways [84]. Some studies demonstrated that overexpression of miR-92, miR-106a, miR-17-5p, and miR-93 in neuroblastoma was functionally linked to MYCN amplification. These miRNAs were correlated with MYCN amplification and activation [85].

To explore the effects and mechanisms of miR-106a on MDR reversal in human glioma cells, Wang et al. knocked down miR-106a in the cisplatin-resistant U87 (U87/DDP) and gefitinib-resistant U251 (U251/G) glioma cell lines and evaluated their sensitivity to drugs, apoptosis rate, and rhodamine 123 content. In addition, they detected decreased expression levels of P-gp, MDR1, MRP1, GST-π, CDX2, ERCC1, RhoE, Bcl-2, Survivin, and Topo-II, as well as reduced production of IL-6, IL-8, and TGF-β in these cell lines. Furthermore, they found decreased expression of p-AKT and transcriptional activation of NF-κB, Twist, AP-1, and Snail in these cell lines. These results suggest that miR-106a is a promising therapeutic target for treating human multidrug-resistant glioma [86].

### 2.11. Breast Cancer

Breast cancer (BC) is the most common cancer among women worldwide, accounting for 25%–30% of all newly diagnosed cancer and exhibiting the second highest mortality among all cancer types in women [87]. Despite advances in early detection and diagnosis, breast cancer’s overall prevalence and survival rate have only marginally improved. Developed countries have maintained a high incidence of breast cancer, whereas the incidence of breast cancer in the developing world is also increasing due to lifestyle changes and longer life expectancies [87,88].

There is not much information published regarding the role of miR-106a in BC. However, recent reports have shown that miRNA-106a promotes breast cancer cell proliferation, clonogenicity, migration, and invasion by inhibiting apoptosis and chemosensitivity [88,89,90]. To identify candidate miRNAs as BC diagnostic biomarkers, Li et al. accomplished a three-phase study to evaluate the expression of 12 miRNAs from the miR-106a-363 cluster in plasma and serum samples. The candidate miRNAs identified utilizing qRT-RCR were subjected to further analysis in breast tissue, plasma exosomes, and serum exosomes. The results showed the significant elevation of miR-106a expression was associated with specific clinical parameters in plasma and serum, indicating the potential roles of this miRNA in BC pathogenesis [89]. MiR-106a overexpression additionally upregulated the levels of Bcl-2 and ABCG2 and downregulated the expression of P53, Bax, and RUNX3 [88]. In an interesting study by Yang et al., miR-106a was among the miRNAs that target ZBTB4a, a transcriptional repressor that inactivates genes through binding their respective GC-rich promoters. These results suggested miR-106a indirectly regulates the enhancer of zeste homolog 2 (EZH2) and its overexpression is inversely correlated with ZBTB4, as has been observed in multiple cancers [91]. Recently, Liu et al. identified DAX-1 as a direct target of miR-106a in BC. DAX-1 is a member of the atypical nuclear receptor family that is downregulated in BC. The authors showed that miR-106a was responsible for promoting the invasion, migration, and proliferation of BC cells by targeting DAX-1. Therefore, the expression level of this miRNA is closely correlated to BC stages, distant metastasis, lymph node metastasis, and poor prognosis [90].

### 2.12. Endometrial Adenocarcinoma

Endometrial cancer (EMC) is a gynecological malignancy with increasing incidence because of today’s lifestyle, particularly in developed countries [92,93].

The evaluation of miRNA expression patterns in EMC cells compared to normal tissue has indicated significant alterations and identified multiple miRNA candidates as diagnostic and prognostic biomarkers [92,94]. In a differential analysis-based study by Wang et al., miR-106a was reported among the five specific prognostic miRNA markers involved in constructing a prognostic model using machine learning [95].

In addition, it was reported that BCL2L11 is one of the main miR-106a targets in EMC. BCL2L11 is a known regulator in the cell cycle and apoptosis. Therefore, miR-106a overexpression correlated with tumor growth, migration, and invasion as the result of BCL2L11 downregulation in EMC tumor cells [96]. Other reports also validated these findings, where knocking down miR-106a notably attenuated EMC cell proliferation and tumor invasiveness while inducing apoptosis [93,95].

Furthermore, a study by Oplawski et al. provided evidence of miR-106a’s involvement in the epithelial–mesenchymal transition (EMT) in endometrial cancer. The researchers not only identified BCL2L as a target of miR-106a but also highlighted its association with EMT [97].

### 2.13. Cervical Cancer

Despite widespread screening for cervical cancer (CC) and significant advances in the development of vaccines, cervical cancer frequently occurs in women worldwide, with high mortality in developing countries [98,99]. Currently, there is no effective treatment for advanced-stage or recurrent cervical cancer. Thus, the search for therapeutically functional molecules or effective targets is critical. Pathogenesis studies have provided reliable evidence for promoting the role of human papillomavirus (HPV) infection in the tumorigenesis of CC [100]. Additional factors, such as changes in levels of cellular proteins that are crucial regulators in certain molecular signaling pathways, are involved in the progression of HPV-infected lesions to cancer [100,101].

There are few reports concerning the dysregulation of miR-106a in CC. However, published results indicate the overexpression of miR-106a in CC cell lines and tissues [98,101,102]. It was revealed that the upregulation of miR-106a was associated with increases in cell migration, invasion, and invasion-related gene expression [98,102]. In addition, miR-106a regulates TIMP-2 by directly binding to its 3’-UTR and inducing the expression of matrix metalloproteinases (MMPs) [102]. On the other hand, the results of clinical sample analysis showed an inverse correlation between miR-106a and TIMP-2 expression since re-expression of TIMP-2 led to the restriction of tumor cell migration, invasion, and MMP expression in CC cells [102].

In an interesting study by Cui et al., the role of miR-106a was studied in HPV-16-positive CC patients. The results showed the overexpression of miR-106a in both HPV-16-positive CC tissues and cell lines [101]. Liver kinase B (LKB1) was also reported as a newly discovered target of miR-106a. MiR-106a appears to play an oncogenic role by targeting the AMPK-mTOR pathway via silencing LKB1. Furthermore, HPV-16 E7 was found to upregulate miR-106a in both E7-expressing and cervical cancer cells [101].

### 2.14. Hepatocellular Carcinoma

Hepatocellular carcinoma (HCC) is one of the most diagnosed malignancies and represents the second leading cause of cancer-related deaths [103,104]. Despite improvements in surgery and other treatments, the overall 5-year survival rate for HCC patients remains extremely low, mainly caused by the tumor’s late detection. Another important reason for poor outcomes is tumor metastasis and recurrence [105,106,107].

As Yuan et al. reported, the overexpression of miR-106a by its promoter hypomethylation contributes to the progression of HCC. The observations confirmed the downregulation of TIMP-2, TP53INP1, and CDKN1A expression in HCC tissues, and these genes were considered as possible miR-106a direct targets [104].

It is known that chemotherapeutic drug gemcitabine-resistant (GR) HCC cells acquire EMT characteristics. EMT, which stands for epithelial–mesenchymal transition, is a phenomenon in which epithelial cells convert into mesenchymal cells through losing epithelial cell–cell junction and epithelial markers (E-cadherin and γ-catenin) while inducing mesenchymal markers (Twist, Vimentin, Snail, and Slug) and is associated with facilitating migration and invasion of HCC tumor cells [108]. Some studies confirmed the drug-resistance role of miR-106a in HCC [105,108,109].

FBXW7 (F-box and WD repeat domain containing 7) is a protein downregulated in some malignancies, including HCC. The published findings indicated the involvement of FBXW7 in suppressing tumor cell migration, invasion, and MDR in cancers [109]. The correlation between FBXW7 and miR-106a in HCC has been assessed by adopting various molecular and cellular methods, such as qRT-PCR, western blotting, luciferase activity, and RNA immunoprecipitation analyses. The results confirmed the upregulation of miR-106a and validated FBXW7 as one of its targets. As shown in one study, decreasing the miR-106a level via elimination by FBXW7 restored the normal features of liver cells [109].

Bioinformatic analyses demonstrated the inverse correlation between long noncoding RNA T cell leukemia/lymphoma 6 (IncTCL6) and miR-106a in HCC [103]. In a study conducted by Luo et al., the functional mechanism of miR-106a and IncTCL6 dysregulation in HCC was investigated, revealing that IncTCL6 directly binds to miR-106a and reduces its expression, resulting in suppressed tumor progression through the PI3K/AKT signaling pathway and restored PTEN expression [103].

In a recent study by Liang et al., downregulation of protein tyrosine phosphatase non-receptor type 12 (PTPN12) in HCC was linked to miR-106a overexpression followed by a significant increase in the proliferation, invasion, and migration of tumor cells [110].

On the contrary, in a study by Wang et al., it was demonstrated that platelet-derived growth factor-D (PDGF-D) mediated EMT through inhibition of miR-106a and subsequent upregulation of Twist1 in HCC GR cells [108]. In line with the role of miRNAs in regulating Twist, this study revealed that miR-106a inhibited cell invasion through the downregulation of Twist1 in HCC GR cells. Since the downregulation of Twist1 reversed EMT to MET (mesenchymal–epithelial transition) in GR cells, miR-106a suppressed EMT partly due to targeting Twist1 [108]. This research group additionally confirmed the tumor suppression behavior of miR-106a in HCC by indicating the role of Fer-1-like family member 4 (FER1L4) in tumor progression via reducing miR-106a and miR-372 levels in the liver. These two miRNAs are believed to inhibit HCC by targeting E2F1 as an NF-kB gene activator at the transcription level [105].

### 2.15. Melanoma

Melanoma is the most dangerous form of skin cancer and accounts for more than 70% of skin tumor-related deaths [111]. A number of studies reviewed miRNA expression patterns as potential biomarkers for screening, early detection, diagnosis, and prognosis of melanoma. Along with other candidates, miR-106a is among those that regulate the highest number of transcription factors in melanoma [112,113,114].

Connexin 43 (Cx43) is a vital gap junction protein in the tumor microenvironment (TME). As Wang et al. reported, the expression of Cx43 is lower in melanoma cells than in human epidermal melanocytes (HEMn) [111]. The lower expression of Cx43 is significantly associated with increased malignancy in multiple cancers [115]. Bioinformatic analyses and luciferase reporter assays confirmed Cx43 as the main target of miR-106a in melanoma cells. The in vitro experiments with melanoma cells also showed a direct correlation between Cx43 overexpression and decreased cell proliferation as well as colony formation ability [111].

On the other hand, in another study by Luan et al., the downregulation of miR-106a was reported. The authors indicated that the overexpression of lncRNA H19 in melanoma was correlated with poor prognosis in patients [116]. According to the tumor-suppressing activity of miR-106a by targeting E2F3 (a member of the E2F transcription factor family), it was demonstrated that lncRNA H19 may function as a sponge of miR-106a-5p to upregulate E2F3 expression and consequently promote glucose metabolism and growth in melanoma cells. Thus, lncRNA H19 may serve as a survival indicator and potential therapeutic target for melanoma patients [116].

### 2.16. Osteosarcoma

Osteosarcoma (OS) is one of the most common types of malignant bone tumors with high mortality rates in children and adolescents, and despite medical interventions such as neoadjuvant chemotherapy and surgery, the survival rate remains low, which suggests poor prognosis [117,118].

In genome-wide miRNA profiling and a cohort study using serum and plasma samples from OS patients and healthy persons, it was discovered that a total of 56 miRNAs were upregulated and 164 miRNAs were downregulated [119]. Subsequently, the researchers validated three miRNAs, miR-21, miR-106a, and miR-221, with elevated levels in the peripheral blood samples of OS patients as compared to healthy controls. In contrast, they did not find that miR-106a was significantly upregulated or downregulated in OS tumors compared to normal bone controls. The increase in miR-106a levels in the plasma of OS patients may be derived from other tissues in the body rather than the tumor itself [119].

In addition, Chen et al. performed a study that indicated the regulating role of miR-106a in the proliferation and invasion of human osteosarcoma cells by targeting vascular non-inflammatory molecule 2 (VNN2) [117]. VNN2 protein is a novel glycosylphosphatidyl inositol-anchored protein member of the VNN family that serves an essential role in the transendothelial migration of cells [120]. The VNN family includes membrane-associated proteins reported to be associated with regulating neutrophil trafficking and adherence. Moreover, the VNN family belongs to a broader pantetheinase family with a role in redox regulation, which may be associated with tumor progression in vitro. The results of this study showed that miR-106a is upregulated in human osteosarcoma cells and the knockdown of miR-106a mediated tumor progression at least partially by targeting VNN2 in osteosarcoma [117].

The upregulation of miR-106a in OS was also confirmed by qRT-PCR and statistical analysis in a study by Zhao et al. The authors demonstrated that the expression of miR-106a was higher in lower OS grades, and this miRNA’s increased level was detected in OS with lung metastasis. Thus, miR-106a was identified as a potential biomarker for early diagnosis and targeted therapy for OS [121].

Furthermore, it was reported that some lncRNAs, including lncRNA HOTAIR (HOX antisense intergenic RNA), are dysregulated in cancer conditions [118]. As Guo et al. reported, the increased level of lncRNA HOTAIR in drug-resistant OS was associated with higher tumor cell proliferation and invasion. Interestingly, they found that lncRNA HOTAIR binds to miR-106a, which is downregulated in DDP-resistant OS. According to the confirmed attenuation of STAT3 by miR-106a, it was concluded that lncRNA HOTAIR overexpression results in the upregulation of STAT3 via inhibiting miR-106a [118].

## 3. Cancer Drug Resistance: The Role of miR-106a

Although the advancement of chemotherapeutic agents in terms of personalized medicine and high-tech development has empowered oncologists to fight cancers, drug resistance still seems to be one of the main challenges in cancer treatment, being responsible for more than 90% of cancer-related mortalities [122,123].

As a complex phenomenon, drug resistance is affected by numerous factors and has cancer-specific features. The mechanisms of miRNA involvement in promoting or suppressing drug resistance are different for each miRNA and each cancer. However, they mainly include alterations in cell cycle checkpoints and drug efflux functions, affecting apoptosis and autophagy, stimulating aberrant DNA damage repair, and inducing abnormal metabolism [122,124,125]. The proven roles of miR-106a in promoting drug resistance in cancers include its involvement in increasing drug clearance or accumulation of oncogenic factors by targeting membrane protein genes. Additionally, miR-106a disruption of apoptosis via targeting key genes and pathways is the critical reason underlying drug resistance in cisplatin-treated patients. Furthermore, it is indicated that miR-106a can modulate drug resistance in colorectal tumors by regulating the properties of cancer stem cells via suppressing tumor suppressor genes [2,42,71,124]. It should be noted that the expression level of miR-106a and its impact on the sensitivity of tumor cells to therapeutics may vary depending on the stage of chemotherapy [122,124] and the mechanism of action of the drug [126].

According to the importance of miRNAs in drug resistance, particularly in cancer chemotherapy, novel approaches have recently emerged for developing medications designed as a combination of chemotherapeutic agents and miRNA-based treatments. Such synergistic strategies for simultaneously targeting tumorigenic pathways and controlling critical miRNA functions may be more effective in improving the clinical outcomes of chemotherapy and prolonging the survival of cancer patients [127,128].

## 4. mir-106a: Dysregulation in Non-Cancer Diseases

### 4.1. Hepatitis B

The Hepatitis B virus (HBV) was one of the first viruses demonstrated to be associated with cancer [129]. Although HBV is well known today as the cause of acute and chronic hepatitis, cirrhosis, and hepatocellular carcinoma, it does not directly cause malignancy [130]. It is believed that HBV infection makes the immune system release complex and abnormal responses that are the cause of liver destruction. Thus, exploring the mechanisms of the immune response in chronic hepatitis B (CHB) patients has been the focus of intense research [130,131].

Continuous release of cytokines by immune cells in response to virus infections frequently results in harmful impacts due to recruiting inflammatory cells, constraining virus replication and spread, and inducing adaptive immunity [132]. In the case of HBV infection, IL-8 is one of the main involved inflammatory cytokines that can stimulate immune responses via granulocytes, NK cells, and T-cell chemotaxis [133].

In a study by Hong et al., the qRT-PCR results suggested that the miR-106a level of peripheral blood mononuclear cells was decreased in CHB patients [134]. Using luciferase activity assays, the authors indicated that IL-8 levels were inversely correlated with miR-106a levels in CHB and they identified IL-8 as a target of miR-106a. Moreover, it was confirmed that IL-8 overexpression in CHB was attenuated by inducing miR-106a to reverse the damage. These results have represented miR-106a as an important player to be considered in the molecular research of CHB [134].

### 4.2. Multiple Sclerosis

Multiple sclerosis (MS) is an autoimmune disease that impacts the brain’s white matter and spinal cord (central nervous system). The rate of MS is higher in women than in men, with a ratio of three to one [135,136]. There are four major types of MS, including primary progressive multiple sclerosis (PPMS), relapsing-remitting multiple sclerosis (RRMS), secondary progressive multiple sclerosis (SPMS), and progressive relapsing multiple sclerosis (PRMS) [136].

With evidence regarding the involvement of miRNAs in modulating the immune response, inflammatory diseases, and autoimmunity [137,138,139], Rahimirad et al. performed a multi-stage experimental study to identify MS-associated miRNAs and their target genes. The qRT-PCR results indicated that miR-106a levels tended to decrease in the blood samples of MS patients. A gene target interaction network of hsa-miR-106a-5p was constructed using the miRTarBase file deposited in Cytoscape and 28 targets with strong support interactions were identified. RBL2, APP, CYP19A1, and BMP2 were upregulated during MS progression and reasonably reported as the potential targets of miR-106a. Hence, miR-106a may be therapeutically valuable in MS condition attenuation [136].

### 4.3. Myasthenia Gravis

Myasthenia gravis (MG) is an antibody-mediated and T cell-dependent autoimmune disease of the neuromuscular junction (NMJ), the pathogenesis of which is poorly understood. About 50%-85% of ocular myasthenia gravis (OMG) patients progress to generalized myasthenia gravis (GMG), and approximately 15%-20% of patients with MG will experience potentially fatal myasthenic crises due to respiratory muscle weakness [140]. In recent years, many studies have revealed that miRNAs are key regulators of MG pathogenesis [140,141].

In a study by Xu et al., the expression of plasma exosomal miRNAs in MG and their involvement in MG pathogenesis were investigated [142]. The authors used deep sequencing and qRT-PCR analyses to reveal that exosomal miR-106a-5p was significantly downregulated in patients with OMG and GMG compared to healthy control subjects. They additionally indicated that levels of this miRNA were notably reduced in patients with GMG compared to OMG [142].

### 4.4. Cardiac Hypertrophy

It is now believed that cardiac hypertrophy is a pathological process, as it can lead to heart failure, arrhythmia, and even sudden death [143]. Cardiac hypertrophy is a condition associated with growth of cardiomyocyte size and the overexpression of many fetal genes, causing left ventricular (LV) wall thickness and diminished normal cardiac function [144].

The reports on miRNA involvement in the regulatory network of cardiovascular disease have shown the upregulation of miR-106a in cardiac hypertrophy, and mitofusin 2 (Mfn2) was identified as the potential target gene for this miRNA. Mfn2, a key member of the mitofusin protein family, is located in the outer membrane of mitochondria and acts to maintain this organelle’s structure [145,146]. Mfn2 is known as a cell proliferation suppressor gene, probably by inhibiting the MAPK/ERK pathway. Recent studies indicate that Mfn2 deficiency is involved in cardiovascular disease [144,146,147].

Based on these facts, a study was conducted by Guan et al. to investigate the role of miR-106a in hypertrophic growth of the heart by targeting Mfn2 using a mouse model of cardiac hypertrophy and a cellular model of cardiomyocyte hypertrophy [144]. They showed that miR-106a overexpression was sufficient to induce hypertrophic growth via directly targeting Mfn2. It was confirmed that the knockdown of miR-106a could inhibit the damage while reversing the hypertrophic alterations. Thus, miR-106a may be a promising molecular target in treating pathological hypertrophy and other cardiac disorders [144,148].

## 5. The Role in Spermatogenesis

Spermatogenesis in male mammals has three phases: mitotic proliferation of spermatogonia, meiosis of spermatocytes, and haploid differentiation of spermatids. The genome of male germ cells is actively transcribed to generate a complex transcriptome with a specific expression pattern in every spermatogenesis phase [149]. According to the importance of the precise regulation needed for successful sperm production, the role of miRNAs has been highlighted particularly for their post-translation effects on stable mRNA expression [150]. Kotaja has comprehensively reviewed spermatogenesis-engaged miRNAs regarding their specific roles in the phases and also their confirmed or potential targets [151].

In a study by He et al. using genetically modified mouse models and various molecular and cellular methodologies, including real-time PCR, microarray, in situ hybridization, immunohistochemistry, etc., the impact of the miRNA expression profile in spermatogenesis was investigated. Additional evidence obtained from clinical evaluation of human spermatozoa or seminal plasma not only confirmed the regulatory role of miRNAs in spermatogenesis but also specified them as potential biomarkers for male factor infertility [152]. Through conducting in vitro and in vivo analyses on mouse spermatogonial stem cells (SSCs), the authors reported that miRNA-20 and miRNA-106a are preferentially expressed in SSCs. They further demonstrated that these two miRNAs promoted post-transcriptional regulation via targeting STAT3 and Ccnd1 and that knockdown of STAT3, Fos, and Ccnd1 resulted in the regeneration of SSCs [152].

## 6. mir-106a and Human Aging

Aging may be the most apparent but still mysterious process in nature. It is accepted that both genetic and non-genetic factors are correlated with the deterioration of body functions due to attenuation of the repair system’s abilities. With all the phenotypic and genotypic alterations, there are always unknown niches to be studied about this condition [153,154].

Many studies have addressed the genes, proteins, and mechanisms involved in this multifactorial process; however, the confirmation of miRNA roles in regulating cellular functions opened new doors for scientists to perform innovative research identifying the miRNA candidates engaged in aging. Although the number of aging-related miRNAs is increasing, their mechanisms of action and specific targets have remained elusive [154,155,156,157].

To address this question, Hackl et al. performed an interesting study (Figure S2 in Supplementary Materials) [153]. They selected four replicative cell aging models, which included endothelial cells, replicated CD8+ T cells, renal proximal tubular epithelial cells, and skin fibroblasts. In addition, they included three organismal aging models, namely foreskin cells, mesenchymal stem cells, and CD8+ T cell populations from old and young donors. Through the utilization of miRNA microarrays using locked nucleic acid technology, a total of four commonly regulated miRNAs were identified. These miRNAs included miR-17, which was downregulated in all seven models; miR-19b and miR-20a, which showed downregulation in six models; and miR-106a, which was downregulated in five models. The reduction in levels of these miRNAs was correlated with an increase in transcript levels of specific target genes, particularly CDK inhibitor p21/CDKN1A. These findings established miRNAs as novel indicators of cell aging in humans [153].

Furthermore, in a miRNA analysis of human longevity at a genome-wide scale, a panel of 863 miRNAs was examined to identify significant differences in miRNA regulation between 15 long-lived individuals (LLI), consisting of centenarians and nonagenarians, and 55 younger individuals [158]. The study design encompassed two distinct experimental approaches: microarrays for initial screening and qRT-PCR for technical validation and replication [158]. The results of this screening study confirmed downregulation of miR-17, miR-106a, and miR-20a in accordance with Hackl et al.’s report [153,158]. Moreover, it was found that the downregulated miRNAs were significantly enriched in disease-associated pathways and had a higher number of reported associations with diseases compared to the upregulated miRNAs. The clustering of these downregulated miRNAs suggested that they may work together in a coordinated manner rather than functioning independently. This finding supports the hypothesis that miRNA dysregulation during aging likely occurs in groups or “packs” rather than as isolated entities [158]. KEGG mapping highlighting miR-106a as a target in the longevity regulating pathway is presented in Figure S3 in Supplementary Materials.

## 7. Perspectives and Conclusions

Since their discovery, miRNAs have remained among hot biology topics and an increasing number of studies have been undertaken to elucidate more about these biomolecules. As presented in this review exploring the dysregulation of miR-106a, this miRNA can be selected for further investigation and validation for application as a biomarker and/or therapeutic target (Table S2 in Supplementary Materials). It cannot be denied that the multi-aspect roles of this miRNA in critical cellular processes make it a great candidate for medical and biomedical applications [159]. 

Many published reports have indicated that most miRNAs hardly pass the validation evaluation to be applied as a biomarker. A wide variety of analysis methods with different levels of sensitivity, lack of standard normalization methods, and challenges in reliable real sample investigation may be the main reasons why miRNAs have not yet entered the market as clinically approved biomarkers [160]. Table 1 lists the current kits and platforms developed for miRNA-based diagnosis.

In the case of miR-106a, determining its precise healthy and non-healthy expression levels in blood and related tissue samples appears to be a primary challenge. Being a circulating miRNA, miR-106a originates from extracellular vesicles released into the blood by various cell types [161]. Despite its remarkable stability in blood, extracting and analyzing miR-106a from these vesicles may present difficulties due to potential false results caused by release from blood cells in response to different conditions [161,162]. Moreover, the levels of circulating miRNAs can be influenced by numerous factors, such as age, gender, ethnicity, medication, and smoking, further complicating the assessment [161,163].

Additionally, as a potential diagnostic tool in clinical settings, miR-106a faces another limitation. It has been detected in patients with different tumor types; however, the results are inconsistent, even among closely related studies of the same diseases. These inconsistencies raise doubts about its reliability and applicability as a universal biomarker in clinical practice [163].

Despite about ten miRNA-based drugs having reached clinical trials, none are in phase III yet, while the number of discontinued studies appears to be increasing (Table 2). In addition to delivery system limitations, dosage concerns, and potential silent side effects, the current regulatory gap covering essential safety and quality attributes for advanced therapies, including miRNA-based therapies, is a bottleneck in translating miRNAs from bench to clinic [163,164].

In an intriguing study, Zhang et al. conducted a review of the challenges in miRNA therapeutics, focusing on the available resources from a drug target perspective [165]. Although miRNA therapeutics showed promise at the outset, the researchers highlighted a phenomenon they termed “too many targets for miRNA effect” (TMTME). This refers to the fact that most miRNAs target tens to hundreds of genes [165]. While miRNAs like miR-106 that participate in multiple regulatory networks and target essential genes in crucial biological pathways seem like attractive therapeutic candidates, developing drugs based on them often proves difficult and complex due to the multiple consequences of altering their levels in the body.

An analysis of unsuccessful miRNA therapeutic candidates revealed that unforeseen side effects emerged during phases I and II of clinical studies, leading to the discontinuation of drug development. These unexpected side effects hindered the advancement of miRNA-based therapies and underscored the challenges in translating miRNA research into successful clinical applications [165].

Therefore, despite the abundance of evidence showcasing the significant regulatory roles of miR-106a in various diseases, including cancers, it is imperative to emphasize the crucial step of validation, particularly through the comprehensive analysis of real samples, before considering the inclusion of this miRNA in pre-clinical or clinical studies. The data on the confirmed target genes of miR-106a, especially those altered in cancer resistance conditions, may be a potential niche that promotes miR-106a to be upgraded in clinical-oriented studies.

## Figures and Tables

**Table 1:** miRNA-based diagnostics in the market.

Product	Platform	Company	Targeted miRNA	Disease/Condition	Development Phase
ThyGeNEXT^®^/ThyraMIR^®^v2	microRNA Pairwise Expression Profiling	Interpace Diagnostics	miR-29b-1-5p miR-31-5p miR-138-1-3p miR-139-5p miR-146b-5p miR-155 miR-204-5p miR-222-3p miR-375 miR-551b-3p	Thyroid cancer	Available-No Certification Information
CogniMIR™	Blood-based assay for assessing plasma levels of specific miRNAs	DIAMIR	miRNA panel is not published	Alzheimer’s disease	Clinical Trial Assay (CTA)
OsteomiR™ kit	qPCR	TAmiRNA	let-7b-5p miR-127-3p miR-1336 miR-141-3p miR-143-3p miR-144-5p miR-152-3p miR-17-5p miR-188-5p miR-19b-3p miR-203a miR-214-3p miR-29b-3p miR-31-5p miR-320a miR-335-5p miR-375 miR-550a-3p miR-582-5p	Bone Quality	Research Use Only (RUO)
ThrombomiR^®^ kit	qPCR	TAmiRNA	hsa-miR-126-3p hsa-miR-223-3p hsa-miR-197-3p hsa-miR-191-5p hsa-miR-24-3p hsa-miR-21-5p hsa-miR-28-3p hsa-miR-320a hsa-miR-150-5p hsa-miR-27b-3p hsa-miR-122-5p	Intrinsic and on-treatment platelet reactivity	Research Use Only (RUO)
HepatomiR^®^ kit	qPCR	TAmiRNA	hsa-miR-122-5p hsa-miR-192-5p hsa-miR-151a-5p	Liver function and disease	CE-IVD (Conformité Européene- In Vitro Diagnostic)
miRisk	qPCR	Hummingbird Diagnostics GmbH	miRNA panel is not published.	Predicts immunotherapy response in stage IV non-small cell lung cancer	Clinical Validation
miLung				Early detection of lung cancer	Clinical Validation

**Table 2:** miRNA-based therapeutics in pre-clinical and clinical phases.

Clinical Trial Number(s) *	Therapeutic Molecule	Targeted miRNA	Mode of Action	Disease	Biotechnology or Biopharmaceutical Company	Stage of Development (Clinical Trial/Preclinical Trial)	Status	Last Update Posted
NCT02508090 NCT02452814 NCT01200420 NCT01872936 NCT01727934 NCT01646489	Miravirsen/SPC3649	miR-122	Anti-miR	Chronic hepatitis C virus	Roche/Santaris Pharma A/S	Phase II	Completed-Unknown (Study has passed its completion date and status has not been verified in more than two years)	February 2018
EudraCT numbers 2015-001535-21 2015-004702-42 2016-002069-77	RG-101	miR-122	Anti-miR	Chronic hepatitis C virus	Regulus Therapeutics Inc.	Phase II	Terminated (Side effects were reported)	September 2017
NCT04536688	RGLS4326	miR-17	Anti-miR	Polycystic kidney disease (PKD)	Regulus Therapeutics Inc.	Phase I	Completed	December 2021
NCT03373786 NCT02855268	RG-012/Lademirsen/SAR339375	miR-21	Anti-miR	Alport Syndrome	Genzyme (a Sanofi Company)	Phase II	Terminated (The results of the futility analysis led to the study termination. No unexpected safety findings were identified)	October 2022
NCT02612662 NCT02826525	RG-125/AZD4076	miR-103/miR-107	Anti-miR	Non-alcoholic Steatohepatitis (NASH)	AstraZeneca	Phase I	Completed	June 2022
NCT05243017 NCT04120493	AMT-130	Artificial miRNA	Artificial miRNA Expression	Huntington Disease	UniQure Biopharma B.V.	Integrated Phase I/II	Ongoing	October 2022
NCT02369198	Mesomir 1	miR-16	miRNA mimic	Malignant Pleural Mesothelioma, Non-Small Cell Lung Cancer	Asbestos Diseases Research Foundation/EnGeneIC Limited	Phase I	Completed	April 2017
NCT05507216 NCT05507203	ABX464/Obefazimod	miR-124	miRNA upregulation	Ulcerative Colitis	Abivax S.A.	Phase III	Ongoing	November 2022
NCT05350969 NCT04045405	CDR132L	miR-132	Anti-miR	Heart failure	Cardior Pharmaceuticals GmbH	Phase II	Ongoing	November 2022
NCT04675996	INT-1B3	miR-193a-3p	miRNA mimic	Solid Tumor	InteRNA	Phase Ib	Ongoing	February 2022
NCT02603224 NCT03601052	Remlarsen/MRG-201	miR-29b	miRNA mimic	Keloid disorder	miRagen Therapeutics, Inc.	Phase II	Completed	August 2021
NCT02580552 NCT03713320 NCT03837457	Cobomarsen/MRG-106	miR-155	Anti-miR	Mycosis fungoides	miRagen Therapeutics, Inc.	Phase II	Terminated (The study was terminated early for business reasons, not due to concerns regarding safety or lack of efficacy)	April 2022
NCT01829971 NCT02862145	MRX34	miR-34a	miRNA mimic	Primary Liver Cancer, SCLC, Lymphoma, Melanoma, Multiple Myeloma, Renal Cell Carcinoma, NSCLC	miRagen Therapeutics, Inc.	Phase I	Prematurely terminated/Withdrawn (5 immune related serious adverse events in Phase 1 study)	September 2016
NCT03603431	MRG-110	miR-92a	Anti-miR	Wounds	miRagen Therapeutics, Inc.	Phase I	Completed	May 2019
_	MGN-2677	miR-143/miR-145	Anti-miR	Vascular Disease	miRagen Therapeutics, Inc.	Pre-clinical	_	_
_	MGN-1374	miR-15/miR-195	Anti-miR	Post-myocardial infection	miRagen Therapeutics, Inc.	Pre-clinical	_	_
_	MRG-107	miR-155	Anti-miR	Amyotrophic lateral sclerosis (ALS)	miRagen Therapeutics, Inc.	Pre-clinical	_	_
_	MGN-4220	miR-29	Anti-miR	Cardiac fibrosis	miRagen Therapeutics, Inc.	Pre-clinical	_	_
_	MGN-4893	miR-451	Anti-miR	Abnormal red blood cell	miRagen Therapeutics, Inc.	Pre-clinical	_	_
_	MGN-5804	miR-378	Anti-miR	Cardiometabolic disease	miRagen Therapeutics, Inc.	Pre-clinical	_	_
_	MGN-6114	miR-92/miR-92a	Anti-miR	Peripheral artery disease (PAD)	miRagen Therapeutics, Inc.	Pre-clinical	_	_
_	MGN-9103	miR-208/miR-208a	Anti-miR	Chronic heart failure	miRagen Therapeutics, Inc.	Pre-clinical	_	_

* NCT numbered trials are registered at ClinicalTrials.gov; EudraCT numbered trials are registered at EU Clinical Trials Register (www.clinicaltrialsregister.eu, accessed on 10 July 2023).

## Data Availability

Not Applicable.

## Supplementary Materials

The following supporting information can be downloaded at: https://www.mdpi.com/article/10.3390/bioengineering10080892/s1, Figure S1: KEGG Maps highlighting miR-106a targets in cancer pathways. The target genes are highlighted in yellow. The graph is rendered by miRPath v4.0.; Figure S2: Experimental design of differential miRNA analysis in Hackl et al. study on alteration in miRNAs’ expression in human aging. Reproduced from [153].; Figure S3: KEGG Maps highlighting miR-106a targets in longevity regulating pathway. The target genes are highlighted in yellow. The graph is rendered by miRPath v4.0.; Table S1: miRDB results for has-miR-106a-5p. There are 1337 predicted targets for hsa-miR-106a-5p in miRDB; Table S2: miR-106a, its regulation alterations, and its target genes by condition type (cancers, diseases, spermatogenesis, and aging).

## Abbreviations

ABCA1	Adenosine triphosphatase-binding cassette A1
ABCG2	Adenosine triphosphatase-binding cassette G2
AKT (PKB)	Serine/threonine protein kinase B
AMPK-mTOR	AMP-activated protein kinase-mammalian target of the rapamycin
AP-1	Activator protein 1
Apaf-1	Apoptotic peptidase activating factor 1
APP	Amyloid precursor protein
ARHGAP24	Rho GTPase-activating protein 24
ATG7	Autophagy-related gene 7
Bak	Bcl-2-antagonist killer
Bax	Bcl-2-associated x protein
BC	Breast cancer
Bcl-1	B-cell lymphoma 1
Bcl-2	B-cell lymphoma 2
Bcl-x	B-cell lymphoma-extra-large
BCL2L11	Bcl-2-like protein 11
Bid	BH3 interacting-domain death agonist
Bim	Bcl-2 interacting mediator of cell death
BMP2	Bone morphogenetic protein 2
CC	Cervical cancer
CCA	Cholangiocarcinoma
Ccnd1	Cyclin D1
CDK	Cyclin-dependent kinase
CDKN1A	Cyclin dependent kinase inhibitor 1A
CDX2	Caudal-type homeobox transcription factor 2
ceRNA	Competing endogenous RNA
CHB	Chronic hepatitis B
CRC	Colorectal cancer
Cx43	Connexin 43
CYP19A1	Cytochrome P450 family 19 subfamily A
DAX-1	Dosage sensitive sex reversal, adrenal hypoplasia, critical region on the X chromosome, gene 1
DDP	Diamminedichloroplatinum
DISC	Death inducing signaling complex
E2F1	E2 promoter binding factor 1 (Transcription factor E2F1)
eIF4A	Eukaryotic initiation factor 4A-I
EMC	Endometrial cancer
EMT	Epithelial–mesenchymal transition
ERCC1	Excision repair cross complementation group 1
ESCC	Esophageal squamous cell carcinoma
EWS	Ewing sarcoma
EZH2	Enhancer of zeste homolog 2
FADD	Fas-associated death domain
FAF-1	FAS-associated factor 1
FASL	FAS ligand
FASTK	Fas-activated serine/threonine kinase
FBXW7	F-box and WD repeat domain containing 7
FER1L4	Long non-coding RNA Fer-1 protein 4
FER1L4	Fer-1 like family member 4
GBMs	Glioblastoma multiforms
GC	Gastric cancer
GMG	Generalized myasthenia gravis
GR	Gemcitabine-resistant
GST-π	Glutathione S-transferase π
HBV	Hepatitis B virus
HCC	Hepatocellular carcinoma
HEMn	Human epidermal melanocytes
HGSOC	High-grade serous ovarian carcinomas
HIPK3	Homeodomain-interacting protein kinase 3
HOTAIR	HOX antisense intergenic RNA
HPV	Human papillomavirus
IGF-1	Insulin-like growth factor-1
IL	Interleukin
IncTCL6	Long noncoding RNA T cell leukemia/lymphoma 6
IRS	Insulin receptor substrates
lncRNA	Long noncoding RNA
lncRNA	Long noncoding RNA
lncRNA XIST, XIST	Long noncoding RNA X inactive specific transcript
MAPK	Mitogen-activated protein kinase
Mcl-1	Myeloid cell leukemia 1
MDR	Multidrug resistance
MDR1, MRP1	Multidrug resistance protein 1
MET	Mesenchymal-epithelial transition
mfn2	Mitofusin 2
MG	Myasthenia gravis
miRNA	Microrna
MMP	Matrix metalloproteinase
MMP2	Mmp2
MRE	Mirna response element
MS	Multiple sclerosis
MYCN	MYC expressed in neuroblastoma
NF-ᴋB	Nuclear factor kappa B
NMJ	Neuromuscular junction
NSCLC	Non-small cell lung cancer
OC	Ovarian cancer
4-Oct	Octamer-binding transcription factor 4
OMG	Ocular myasthenia gravis
OS	Osteosarcoma
PA	Pilocytic astrocytomas
PAK7	P21-activated kinase 7
PARP	Poly-ADP ribose polymerase
PC	Pancreatic cancer
PDCD4	Programmed cell death 4
PDGF-D	Platelet-derived growth factor-D
PDK1	Phosphoinositide-dependent protein kinase 1
P-gp	P-glycoprotein
PI3K	Phosphatidylinositol 3-kinase
PIP2	Phosphatidylinositol diphosphate
PIP3	Phosphatidylinositol trisphosphate
PNET	Primitive neuroectodermal tumor
PPMS	Primary progressive multiple sclerosis
PRMS	Progressive relapsing multiple sclerosis
PTEN	Phosphatase and tensin homolog deleted on chromosome 10
PTPN12	Protein tyrosine phosphatase non-receptor type 12
RB1	Retinoblastoma protein 1
RBL2	Retinoblastoma 2
RCC	Renal cell carcinoma
RNA	Ribonucleic acid
RRMS	Relapsing-remitting multiple sclerosis
RT-PCR	Real-time reverse transcriptase PCR
RUNX1	Runt-related transcription factor 1
RUNX2	Runt-related transcription factor 2
RUNX3	Runt-related transcription factor 3
SLC2A3, GLUT3	Solute carrier family 2 member 3
SOX9	Sry-box transcription factor 9
SPMS	Secondary progressive multiple sclerosis
SSCs	Spermatogonial stem cells
STAT3	Signal transducers and activators of transcription 3
TGFBR2	Transforming growth factor-b receptor 2
TGF-β	Transforming growth factor-β
TIMP2	Tissue inhibitors of metalloproteinase 2
TME	Tumor microenvironment
TNF	Tumor necrosis factor
Topo-II	DNA-topoisomerase II
TP53INP1	Tumor protein p53-inducible nuclear protein 1
TRAIL	TNF-related apoptosis-inducing ligand
VEGF	Vascular endothelial growth factor
VEGFA	Endogenous vascular endothelial growth factor-a
VNN2	Vascular non-inflammatory molecule 2
WHO	World Health Organization
WNT	Wingless-related integration site
