# Supplementary material for: Overview of miR-106a Regulatory Roles: from Cancer to Aging
Source: Bioengineering (Basel). 2023 Jul 27;10(8):892. doi: 10.3390/bioengineering10080892 (PMC10451182; doi:10.3390/bioengineering10080892)
Supplement: Supplementary file 1 [file bioengineering-10-00892-s001.zip › bioengineering-2510123-supplementary.pdf]

bioengineering

MDPI

Supplementary Materials

Overview of miR-106a Regulatory Roles: from Cancer to Aging

Maryam Daneshpour [1,*] and Ali Ghadimi-Daresajini [2]

1 Biotechnology Department, School of Advanced Technologies in Medicine, Shahid Beheshti University of Medical Sciences, Tehran 1985717443, Iran
2 Department of Medical Biotechnology, School of Allied Medicine, Cellular and Molecular Research Center, Iran University of Medical Sciences, Tehran 1449614535, Iran; ghadimi.a@tak.iums.ac.ir
* Correspondence: m20daneshpour@gmail.com

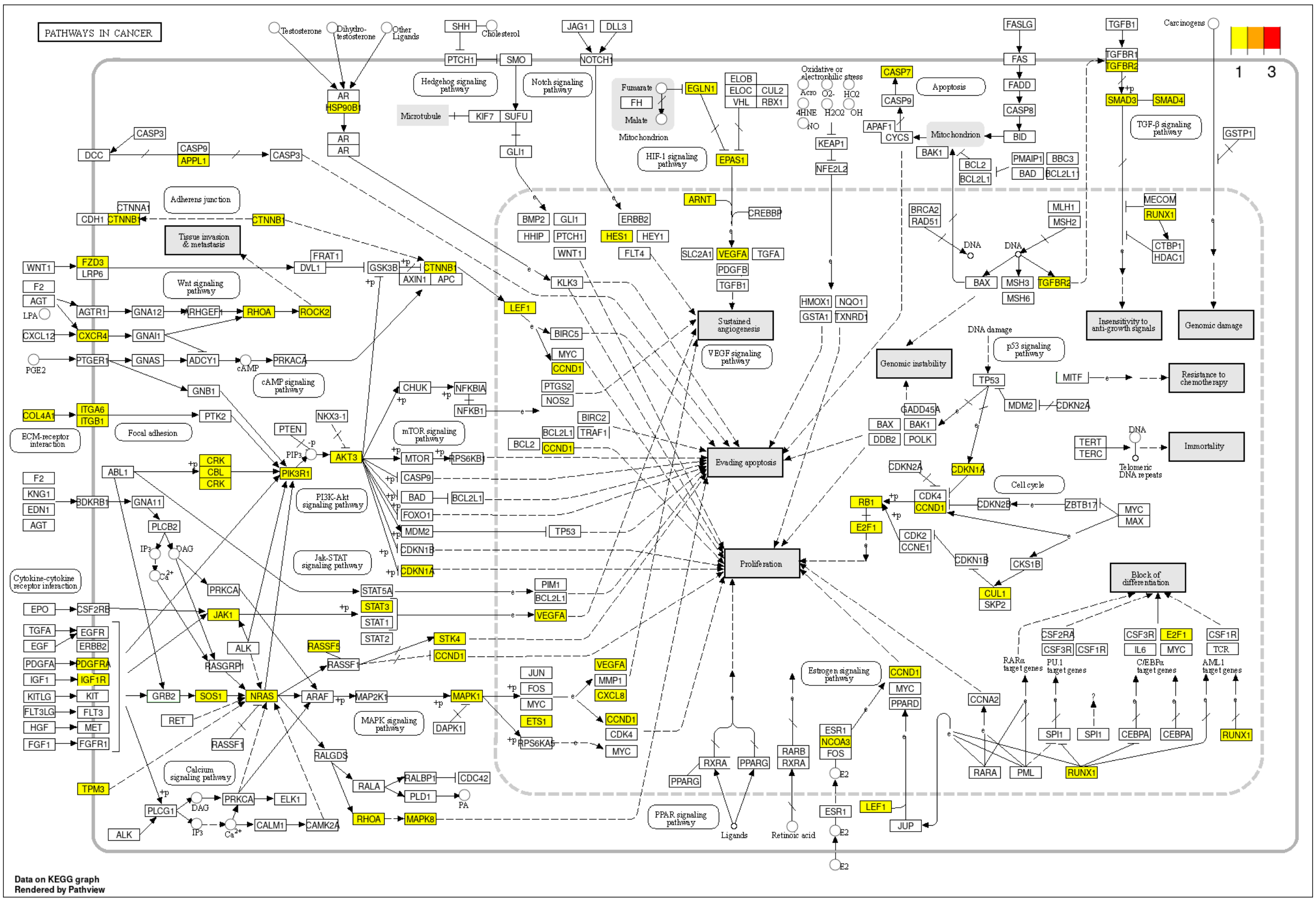

Figure S1. KEGG Maps highlighting miR-106a targets in cancer pathways. The target genes are highlighted in yellow. The graph is rendered by miRPath v4.0, accessed on 20 July 2023.

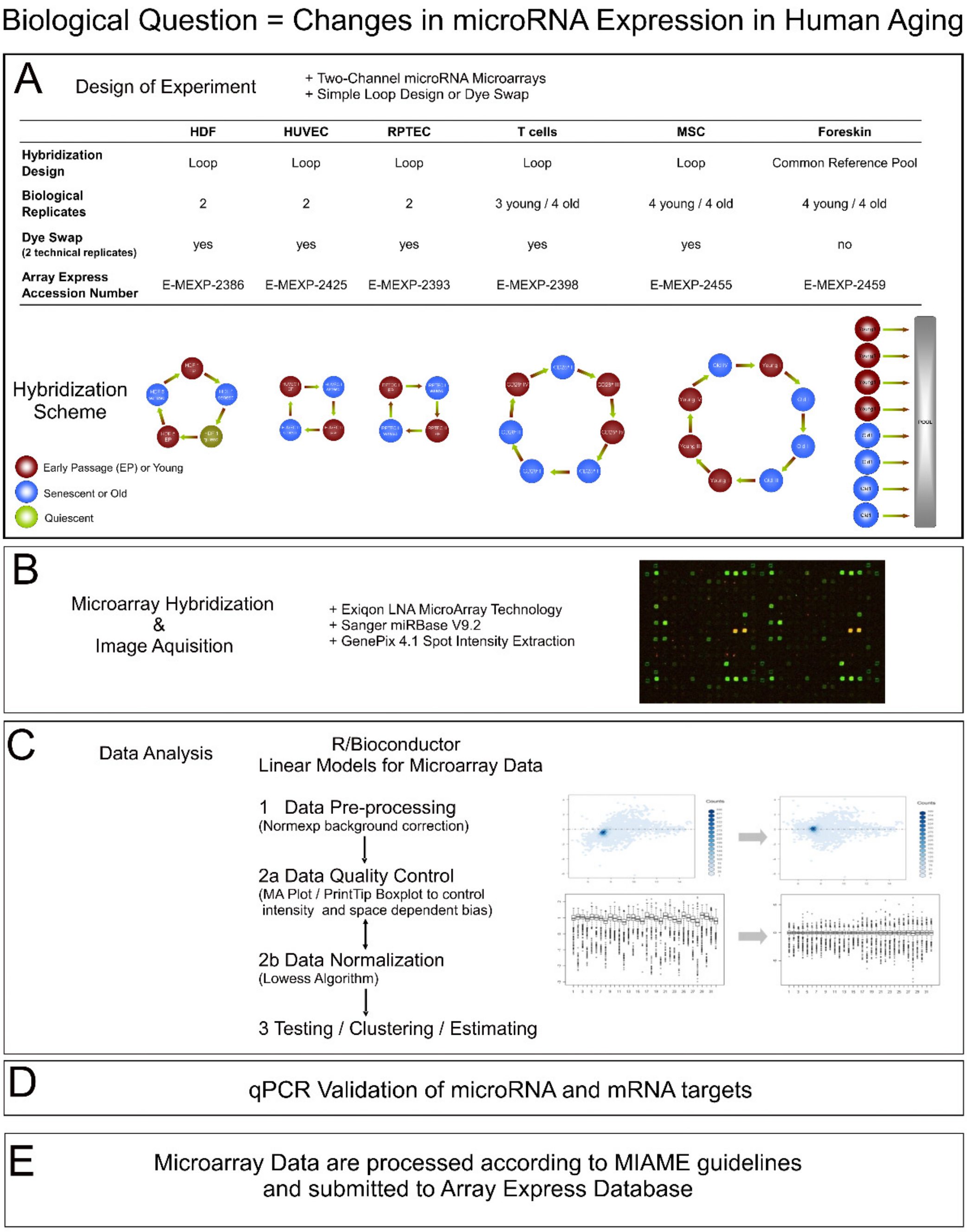

Figure S2. Experimental design of differential miRNA analysis in Hackl et al. study on alteration in miRNAs' expression in human aging. Reproduced from [153].

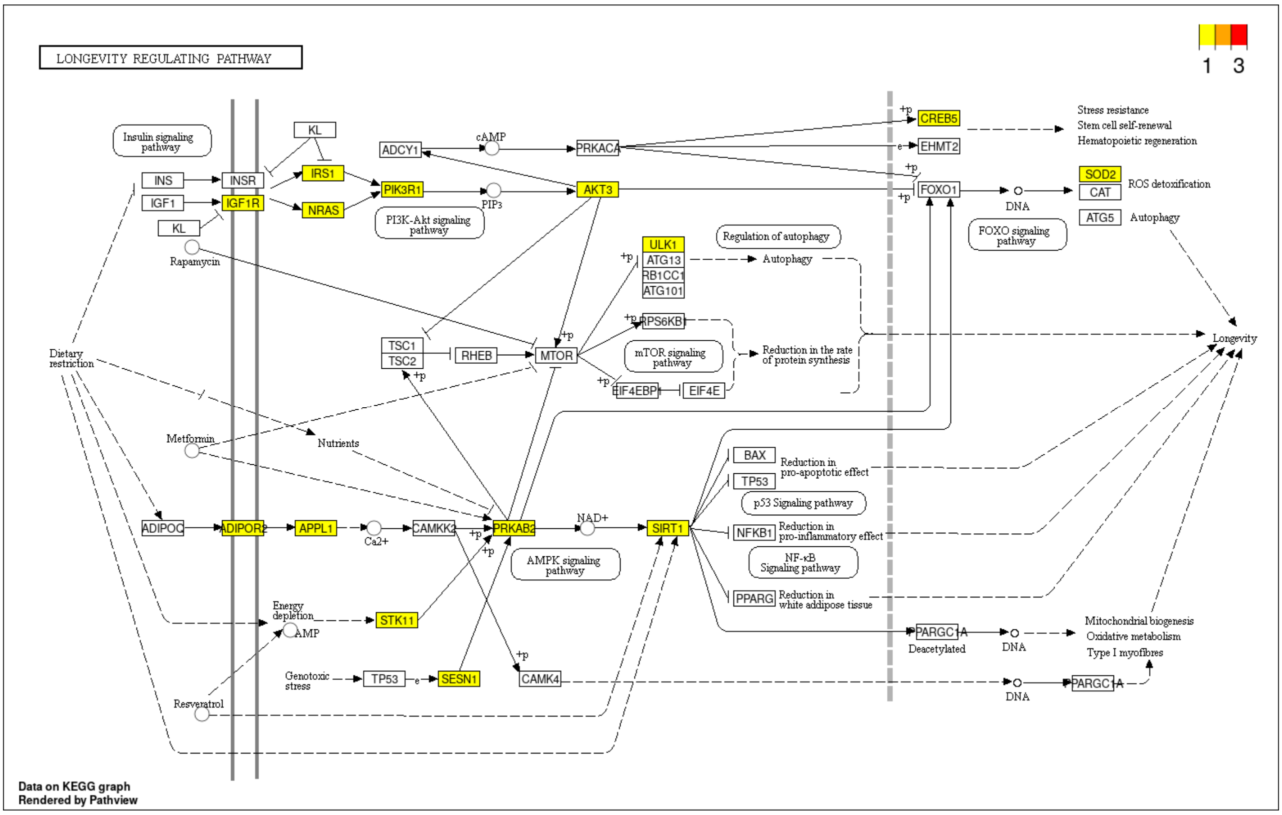

Figure S3. KEGG Maps highlighting miR-106a targets in longevity regulating pathway. The target genes are highlighted in yellow. The graph is rendered by miRPath v4.0, accessed on 20 July 2023.

Table S1. miRDB results for has-miR-106a-5p. There are 1337 predicted targets for hsa-miR-106a-5p in miRDB.

Target Rank	Target Score	miRNA Name	Gene Symbol	Gene Description
1	100	hsa-miR-106a-5p	ENPP5	ectonucleotide pyrophosphatase/phosphodiesterase 5 (putative)
2	100	hsa-miR-106a-5p	GPR137C	G protein-coupled receptor 137C
3	100	hsa-miR-106a-5p	FYCO1	FYVE and coiled-coil domain containing 1
4	100	hsa-miR-106a-5p	DYNC1LI2	dynein cytoplasmic 1 light intermediate chain 2
5	100	hsa-miR-106a-5p	ZNFX1	zinc finger NFX1-type containing 1
6	99	hsa-miR-106a-5p	NPAT	nuclear protein, coactivator of histone transcription
7	99	hsa-miR-106a-5p	ARID4B	AT-rich interaction domain 4B

8	99	hsa-miR-106a-5p	MED12L	mediator complex subunit 12 like
9	99	hsa-miR-106a-5p	TBC1D20	TBC1 domain family member 20
10	99	hsa-miR-106a-5p	ZNF800	zinc finger protein 800
11	99	hsa-miR-106a-5p	NPAS2	neuronal PAS domain protein 2
12	99	hsa-miR-106a-5p	SAR1B	secretion associated Ras related GTPase 1B
13	99	hsa-miR-106a-5p	BRMS1L	BRMS1 like transcriptional repressor
14	99	hsa-miR-106a-5p	ITGB8	integrin subunit beta 8
15	99	hsa-miR-106a-5p	ANKRD52	ankyrin repeat domain 52
16	99	hsa-miR-106a-5p	VLDLR	very low density lipoprotein receptor
17	99	hsa-miR-106a-5p	TXNIP	thioredoxin interacting protein
18	99	hsa-miR-106a-5p	KCNB1	potassium voltage-gated channel subfamily B member 1
19	99	hsa-miR-106a-5p	CLOCK	clock circadian regulator
20	99	hsa-miR-106a-5p	RUFY2	RUN and FYVE domain containing 2
21	99	hsa-miR-106a-5p	ARHGAP12	Rho GTPase activating protein 12
22	99	hsa-miR-106a-5p	KCNK10	potassium two pore domain channel subfamily K member 10
23	99	hsa-miR-106a-5p	STK17B	serine/threonine kinase 17b
24	99	hsa-miR-106a-5p	PDCD1LG2	programmed cell death 1 ligand 2
25	99	hsa-miR-106a-5p	ZFYVE26	zinc finger FYVE-type containing 26
26	99	hsa-miR-106a-5p	RAB22A	RAB22A, member RAS oncogene family
27	99	hsa-miR-106a-5p	SLC40A1	solute carrier family 40 member 1
28	99	hsa-miR-106a-5p	REEP3	receptor accessory protein 3
29	99	hsa-miR-106a-5p	RRAGD	Ras related GTP binding D

30	99	hsa-miR-106a-5p	TBC1D9	TBC1 domain family member 9
31	99	hsa-miR-106a-5p	CFL2	cofilin 2
32	99	hsa-miR-106a-5p	PTPN4	protein tyrosine phosphatase, non-receptor type 4
33	99	hsa-miR-106a-5p	AAK1	AP2 associated kinase 1
34	99	hsa-miR-106a-5p	SACS	sacsin molecular chaperone
35	99	hsa-miR-106a-5p	PKD2	polycystin 2, transient receptor potential cation channel
36	99	hsa-miR-106a-5p	ZNF827	zinc finger protein 827
37	99	hsa-miR-106a-5p	MAP3K2	mitogen-activated protein kinase kinase kinase 2
38	99	hsa-miR-106a-5p	NAPEPLD	N-acyl phosphatidylethanolamine phospholipase D
39	98	hsa-miR-106a-5p	EPHA4	EPH receptor A4
40	98	hsa-miR-106a-5p	FAM45A	family with sequence similarity 45 member A
41	98	hsa-miR-106a-5p	EPHA5	EPH receptor A5
42	98	hsa-miR-106a-5p	GPR6	G protein-coupled receptor 6
43	98	hsa-miR-106a-5p	E2F1	E2F transcription factor 1
44	98	hsa-miR-106a-5p	AKTIP	AKT interacting protein
45	98	hsa-miR-106a-5p	FCHO2	FCH domain only 2
46	98	hsa-miR-106a-5p	USP46	ubiquitin specific peptidase 46
47	98	hsa-miR-106a-5p	NFAT5	nuclear factor of activated T cells 5
48	98	hsa-miR-106a-5p	C2CD2	C2 calcium dependent domain containing 2
49	98	hsa-miR-106a-5p	RNF128	ring finger protein 128, E3 ubiquitin protein ligase
50	98	hsa-miR-106a-5p	EZH1	enhancer of zeste 1 polycomb repressive complex 2 subunit
51	98	hsa-miR-106a-5p	RGL1	ral guanine nucleotide dissociation stimulator like 1
52	98	hsa-miR-106a-5p	MKRN1	makorin ring finger protein 1
53	98	hsa-miR-106a-5p	ITPRIPL2	ITPRIP like 2
54	98	hsa-miR-106a-5p	ZNF367	zinc finger protein 367

55	98	hsa-miR-106a-5p	PLEKHA3	pleckstrin homology domain containing A3
56	98	hsa-miR-106a-5p	ANKIB1	ankyrin repeat and IBR domain containing 1
57	98	hsa-miR-106a-5p	REST	RE1 silencing transcription factor
58	98	hsa-miR-106a-5p	KLHL28	kelch like family member 28
59	98	hsa-miR-106a-5p	KIAA0513	KIAA0513
60	97	hsa-miR-106a-5p	FBXL5	F-box and leucine rich repeat protein 5
61	97	hsa-miR-106a-5p	NCOA3	nuclear receptor coactivator 3
62	97	hsa-miR-106a-5p	CCND1	cyclin D1
63	97	hsa-miR-106a-5p	USP3	ubiquitin specific peptidase 3
64	97	hsa-miR-106a-5p	FRMD6	FERM domain containing 6
65	97	hsa-miR-106a-5p	VANGL1	VANGL planar cell polarity protein 1
66	97	hsa-miR-106a-5p	ZFYVE9	zinc finger FYVE-type containing 9
67	97	hsa-miR-106a-5p	RPS6KA5	ribosomal protein S6 kinase A5
68	97	hsa-miR-106a-5p	ATXN1L	ataxin 1 like
69	97	hsa-miR-106a-5p	CAMTA1	calmodulin binding transcription activator 1
70	97	hsa-miR-106a-5p	ATG16L1	autophagy related 16 like 1
71	97	hsa-miR-106a-5p	LRIG1	leucine rich repeats and immunoglobulin like domains 1
72	97	hsa-miR-106a-5p	ZBTB4	zinc finger and BTB domain containing 4
73	97	hsa-miR-106a-5p	SAMD12	sterile alpha motif domain containing 12
74	97	hsa-miR-106a-5p	HSPA8	heat shock protein family A (Hsp70) member 8
75	97	hsa-miR-106a-5p	RASGRF2	Ras protein specific guanine nucleotide releasing factor 2
76	97	hsa-miR-106a-5p	ELK4	ELK4, ETS transcription factor

77	97	hsa-miR-106a-5p	EMSY	EMSY, BRCA2 interacting transcriptional repressor
78	97	hsa-miR-106a-5p	BCL11B	BCL11B, BAF complex component
79	97	hsa-miR-106a-5p	NABP1	nucleic acid binding protein 1
80	97	hsa-miR-106a-5p	GLIS3	GLIS family zinc finger 3
81	97	hsa-miR-106a-5p	KMT2B	lysine methyltransferase 2B
82	97	hsa-miR-106a-5p	ZNF512B	zinc finger protein 512B
83	97	hsa-miR-106a-5p	FSD1L	fibronectin type III and SPRY domain containing 1 like
84	96	hsa-miR-106a-5p	VASH2	vasohibin 2
85	96	hsa-miR-106a-5p	RBL2	RB transcriptional corepressor like 2
86	96	hsa-miR-106a-5p	STXBP5	syntaxin binding protein 5
87	96	hsa-miR-106a-5p	AGTPBP1	ATP/GTP binding protein 1
88	96	hsa-miR-106a-5p	MFSD8	major facilitator superfamily domain containing 8
89	96	hsa-miR-106a-5p	FAM126B	family with sequence similarity 126 member B
90	96	hsa-miR-106a-5p	CMTR2	cap methyltransferase 2
91	96	hsa-miR-106a-5p	TNFRSF21	TNF receptor superfamily member 21
92	96	hsa-miR-106a-5p	TGFBR2	transforming growth factor beta receptor 2
93	96	hsa-miR-106a-5p	MYT1L	myelin transcription factor 1 like
94	96	hsa-miR-106a-5p	SEMA7A	semaphorin 7A (John Milton Hagen blood group)
95	96	hsa-miR-106a-5p	ZFP91	ZFP91 zinc finger protein
96	96	hsa-miR-106a-5p	HS3ST5	heparan sulfate-glucosamine 3-sulfotransferase 5
97	96	hsa-miR-106a-5p	BNIP2	BCL2 interacting protein 2

98	96	hsa-miR-106a-5p	SPRED1	sprouty related EVH1 domain containing 1
99	96	hsa-miR-106a-5p	MAP3K14	mitogen-activated protein kinase kinase kinase 14
100	96	hsa-miR-106a-5p	ATAD2	ATPase family, AAA domain containing 2
101	96	hsa-miR-106a-5p	ITGA4	integrin subunit alpha 4
102	96	hsa-miR-106a-5p	PHC3	polyhomeotic homolog 3
103	96	hsa-miR-106a-5p	FAM129A	family with sequence similarity 129 member A
104	96	hsa-miR-106a-5p	DPYSL5	dihydropyrimidinase like 5
105	96	hsa-miR-106a-5p	USP31	ubiquitin specific peptidase 31
106	96	hsa-miR-106a-5p	ZNF652	zinc finger protein 652
107	96	hsa-miR-106a-5p	ZNF704	zinc finger protein 704
108	96	hsa-miR-106a-5p	LAPTM4A	lysosomal protein transmembrane 4 alpha
109	96	hsa-miR-106a-5p	EIF5A2	eukaryotic translation initiation factor 5A2
110	96	hsa-miR-106a-5p	FOXJ3	forkhead box J3
111	96	hsa-miR-106a-5p	TET3	tet methylcytosine dioxygenase 3
112	96	hsa-miR-106a-5p	FRS2	fibroblast growth factor receptor substrate 2
113	96	hsa-miR-106a-5p	CNOT4	CCR4-NOT transcription complex subunit 4
114	96	hsa-miR-106a-5p	FJX1	four-jointed box kinase 1
115	96	hsa-miR-106a-5p	NR2C2	nuclear receptor subfamily 2 group C member 2
116	96	hsa-miR-106a-5p	GAB1	GRB2 associated binding protein 1
117	96	hsa-miR-106a-5p	ANKRD29	ankyrin repeat domain 29
118	96	hsa-miR-106a-5p	ZC3H12C	zinc finger CCCH-type containing 12C
119	96	hsa-miR-106a-5p	ARID4A	AT-rich interaction domain 4A
120	95	hsa-miR-106a-5p	KAT2B	lysine acetyltransferase 2B

121	95	hsa-miR-106a-5p	ZBTB41	zinc finger and BTB domain containing 41
122	95	hsa-miR-106a-5p	U2SURP	U2 snRNP associated SURP domain containing
123	95	hsa-miR-106a-5p	GPR63	G protein-coupled receptor 63
124	95	hsa-miR-106a-5p	NCKAP5	NCK associated protein 5
125	95	hsa-miR-106a-5p	UBXN2A	UBX domain protein 2A
126	95	hsa-miR-106a-5p	FAM117B	family with sequence similarity 117 member B
127	95	hsa-miR-106a-5p	SALL3	spalt like transcription factor 3
128	95	hsa-miR-106a-5p	LAMA3	laminin subunit alpha 3
129	95	hsa-miR-106a-5p	ZBTB7A	zinc finger and BTB domain containing 7A
130	95	hsa-miR-106a-5p	CEP97	centrosomal protein 97
131	95	hsa-miR-106a-5p	B3GALT2	beta-1,3-galactosyltransferase 2
132	95	hsa-miR-106a-5p	TRIP10	thyroid hormone receptor interactor 10
133	95	hsa-miR-106a-5p	SALL1	spalt like transcription factor 1
134	95	hsa-miR-106a-5p	GXYLT1	glucoside xylosyltransferase 1
135	95	hsa-miR-106a-5p	LPGAT1	lysophosphatidylglycerol acyltransferase 1
136	95	hsa-miR-106a-5p	F3	coagulation factor III, tissue factor
137	95	hsa-miR-106a-5p	SPOPL	speckle type BTB/POZ protein like
138	95	hsa-miR-106a-5p	E2F5	E2F transcription factor 5
139	95	hsa-miR-106a-5p	ZXDA	zinc finger X-linked duplicated A
140	95	hsa-miR-106a-5p	SERTAD2	SERTA domain containing 2
141	95	hsa-miR-106a-5p	FGD4	FYVE, RhoGEF and PH domain containing 4
142	95	hsa-miR-106a-5p	PRRG1	proline rich and Gla domain 1
143	95	hsa-miR-106a-5p	ANKRD33B	ankyrin repeat domain 33B

144	95	hsa-miR-106a-5p	PEX5L	peroxisomal biogenesis factor 5 like
145	95	hsa-miR-106a-5p	FGD5	FYVE, RhoGEF and PH domain containing 5
146	95	hsa-miR-106a-5p	PTHLH	parathyroid hormone like hormone
147	95	hsa-miR-106a-5p	TRIM36	tripartite motif containing 36
148	95	hsa-miR-106a-5p	CHRM2	cholinergic receptor muscarinic 2
149	95	hsa-miR-106a-5p	EGLN3	egl-9 family hypoxia inducible factor 3
150	95	hsa-miR-106a-5p	PURB	purine rich element binding protein B
151	95	hsa-miR-106a-5p	SLC24A2	solute carrier family 24 member 2
152	95	hsa-miR-106a-5p	UEVLD	UEV and lactate/malate dehyrogenase domains
153	95	hsa-miR-106a-5p	EFCAB14	EF-hand calcium binding domain 14
154	95	hsa-miR-106a-5p	PPP6C	protein phosphatase 6 catalytic subunit
155	95	hsa-miR-106a-5p	HAS2	hyaluronan synthase 2
156	95	hsa-miR-106a-5p	LIMK1	LIM domain kinase 1
157	95	hsa-miR-106a-5p	SGMS1	sphingomyelin synthase 1
158	95	hsa-miR-106a-5p	RORA	RAR related orphan receptor A
159	94	hsa-miR-106a-5p	LIMA1	LIM domain and actin binding 1
160	94	hsa-miR-106a-5p	EPHA7	EPH receptor A7
161	94	hsa-miR-106a-5p	PAK5	p21 (RAC1) activated kinase 5
162	94	hsa-miR-106a-5p	SLC46A3	solute carrier family 46 member 3
163	94	hsa-miR-106a-5p	MASTL	microtubule associated serine/threonine kinase like
164	94	hsa-miR-106a-5p	PDE3B	phosphodiesterase 3B
165	94	hsa-miR-106a-5p	PTPRD	protein tyrosine phosphatase, receptor type D
166	94	hsa-miR-106a-5p	PGM2L1	phosphoglucomutase 2 like 1

167	94	hsa-miR-106a-5p	SLC17A7	solute carrier family 17 member 7
168	94	hsa-miR-106a-5p	AGO1	argonaute RISC catalytic component 1
169	94	hsa-miR-106a-5p	UBE2Q2	ubiquitin conjugating enzyme E2 Q2
170	94	hsa-miR-106a-5p	RASD1	ras related dexamethasone induced 1
171	94	hsa-miR-106a-5p	RAB11FIP5	RAB11 family interacting protein 5
172	94	hsa-miR-106a-5p	PFKP	phosphofructokinase, platelet
173	94	hsa-miR-106a-5p	IRF9	interferon regulatory factor 9
174	94	hsa-miR-106a-5p	FAT2	FAT atypical cadherin 2
175	94	hsa-miR-106a-5p	BTG3	BTG anti-proliferation factor 3
176	94	hsa-miR-106a-5p	BBX	BBX, HMG-box containing
177	94	hsa-miR-106a-5p	SSH1	slingshot protein phosphatase 1
178	94	hsa-miR-106a-5p	FBXO48	F-box protein 48
179	94	hsa-miR-106a-5p	SOS1	SOS Ras/Rac guanine nucleotide exchange factor 1
180	94	hsa-miR-106a-5p	RASL11B	RAS like family 11 member B
181	94	hsa-miR-106a-5p	SNTB2	syntrophin beta 2
182	94	hsa-miR-106a-5p	PRDM6	PR/SET domain 6
183	94	hsa-miR-106a-5p	TRIP11	thyroid hormone receptor interactor 11
184	94	hsa-miR-106a-5p	MKNK2	MAP kinase interacting serine/threonine kinase 2
185	94	hsa-miR-106a-5p	VSX1	visual system homeobox 1
186	94	hsa-miR-106a-5p	ARMC8	armadillo repeat containing 8
187	94	hsa-miR-106a-5p	TOPORS	TOP1 binding arginine/serine rich protein
188	94	hsa-miR-106a-5p	ZBTB20	zinc finger and BTB domain containing 20
189	94	hsa-miR-106a-5p	CLIP4	CAP-Gly domain containing linker protein family member 4

190	94	hsa-miR-106a-5p	KMT2A	lysine methyltransferase 2A
191	94	hsa-miR-106a-5p	FZD3	frizzled class receptor 3
192	93	hsa-miR-106a-5p	RORC	RAR related orphan receptor C
193	93	hsa-miR-106a-5p	BMPR2	bone morphogenetic protein receptor type 2
194	93	hsa-miR-106a-5p	TMEM127	transmembrane protein 127
195	93	hsa-miR-106a-5p	ABCA1	ATP binding cassette subfamily A member 1
196	93	hsa-miR-106a-5p	NEDD4L	neural precursor cell expressed, developmentally down-regulated 4-like, E3 ubiquitin protein ligase
197	93	hsa-miR-106a-5p	IQSEC2	IQ motif and Sec7 domain 2
198	93	hsa-miR-106a-5p	DCBLD2	discoidin, CUB and LCCL domain containing 2
199	93	hsa-miR-106a-5p	DUSP2	dual specificity phosphatase 2
200	93	hsa-miR-106a-5p	ANKFY1	ankyrin repeat and FYVE domain containing 1
201	93	hsa-miR-106a-5p	ANKH	ANKH inorganic pyrophosphate transport regulator
202	93	hsa-miR-106a-5p	DRD1	dopamine receptor D1
203	93	hsa-miR-106a-5p	KIF3B	kinesin family member 3B
204	93	hsa-miR-106a-5p	BNC2	basonuclin 2
205	93	hsa-miR-106a-5p	HEG1	heart development protein with EGF like domains 1
206	93	hsa-miR-106a-5p	RAB5B	RAB5B, member RAS oncogene family
207	93	hsa-miR-106a-5p	NKIRAS1	NFKB inhibitor interacting Ras like 1
208	93	hsa-miR-106a-5p	NIN	ninein
209	93	hsa-miR-106a-5p	JPT1	Jupiter microtubule associated homolog 1
210	93	hsa-miR-106a-5p	ARHGAP26	Rho GTPase activating protein 26
211	93	hsa-miR-106a-5p	LDLRAP1	low density lipoprotein receptor adaptor protein 1

212	93	hsa-miR-106a-5p	GPR137B	G protein-coupled receptor 137B
213	93	hsa-miR-106a-5p	DNAJC27	DnaJ heat shock protein family (Hsp40) member C27
214	93	hsa-miR-106a-5p	ADARB1	adenosine deaminase, RNA specific B1
215	93	hsa-miR-106a-5p	NAGK	N-acetylglucosamine kinase
216	93	hsa-miR-106a-5p	SLITRK3	SLIT and NTRK like family member 3
217	92	hsa-miR-106a-5p	MAP3K9	mitogen-activated protein kinase kinase kinase 9
218	92	hsa-miR-106a-5p	ADAM9	ADAM metallopeptidase domain 9
219	92	hsa-miR-106a-5p	TMEM168	transmembrane protein 168
220	92	hsa-miR-106a-5p	PCDHA8	protocadherin alpha 8
221	92	hsa-miR-106a-5p	PCDHAC1	protocadherin alpha subfamily C, 1
222	92	hsa-miR-106a-5p	L3MBTL3	L3MBTL3, histone methyl-lysine binding protein
223	92	hsa-miR-106a-5p	CCNG2	cyclin G2
224	92	hsa-miR-106a-5p	TAOK3	TAO kinase 3
225	92	hsa-miR-106a-5p	DDHD1	DDHD domain containing 1
226	92	hsa-miR-106a-5p	PPP1R15B	protein phosphatase 1 regulatory subunit 15B
227	92	hsa-miR-106a-5p	ANKRD17	ankyrin repeat domain 17
228	92	hsa-miR-106a-5p	PCDHAC2	protocadherin alpha subfamily C, 2
229	92	hsa-miR-106a-5p	PCDHA13	protocadherin alpha 13
230	92	hsa-miR-106a-5p	RRM2	ribonucleotide reductase regulatory subunit M2
231	92	hsa-miR-106a-5p	DNAJC16	DnaJ heat shock protein family (Hsp40) member C16
232	92	hsa-miR-106a-5p	ATL3	atlastin GTPase 3
233	92	hsa-miR-106a-5p	HBP1	HMG-box transcription factor 1
234	92	hsa-miR-106a-5p	PCDHA10	protocadherin alpha 10

235	92	hsa-miR-106a-5p	HPS5	HPS5, biogenesis of lysosomal organelles complex 2 subunit 2
236	92	hsa-miR-106a-5p	PCDHA5	protocadherin alpha 5
237	92	hsa-miR-106a-5p	IGSF10	immunoglobulin superfamily member 10
238	92	hsa-miR-106a-5p	XRN1	5'-3' exoribonuclease 1
239	92	hsa-miR-106a-5p	RACGAP1	Rac GTPase activating protein 1
240	92	hsa-miR-106a-5p	URI1	URI1, prefoldin like chaperone
241	92	hsa-miR-106a-5p	ANO6	anoctamin 6
242	92	hsa-miR-106a-5p	PCDHA6	protocadherin alpha 6
243	92	hsa-miR-106a-5p	PCDHA11	protocadherin alpha 11
244	92	hsa-miR-106a-5p	KLHL2	kelch like family member 2
245	92	hsa-miR-106a-5p	PCDHA3	protocadherin alpha 3
246	92	hsa-miR-106a-5p	PTPN3	protein tyrosine phosphatase, non-receptor type 3
247	92	hsa-miR-106a-5p	LASP1	LIM and SH3 protein 1
248	92	hsa-miR-106a-5p	OCRL	OCRL, inositol polyphosphate-5-phosphatase
249	92	hsa-miR-106a-5p	SUCO	SUN domain containing ossification factor
250	92	hsa-miR-106a-5p	RAP2C	RAP2C, member of RAS oncogene family
251	92	hsa-miR-106a-5p	ACSL4	acyl-CoA synthetase long chain family member 4
252	92	hsa-miR-106a-5p	SUSD6	sushi domain containing 6
253	92	hsa-miR-106a-5p	PCDHA4	protocadherin alpha 4
254	92	hsa-miR-106a-5p	SRCIN1	SRC kinase signaling inhibitor 1
255	92	hsa-miR-106a-5p	PCDHA1	protocadherin alpha 1
256	92	hsa-miR-106a-5p	BRWD1	bromodomain and WD repeat domain containing 1
257	92	hsa-miR-106a-5p	ELK3	ELK3, ETS transcription factor
258	92	hsa-miR-106a-5p	CEP120	centrosomal protein 120
259	92	hsa-miR-106a-5p	PCDHA7	protocadherin alpha 7
260	92	hsa-miR-106a-5p	PCDHA2	protocadherin alpha 2

261	92	hsa-miR-106a-5p	PCDHA12	protocadherin alpha 12
262	92	hsa-miR-106a-5p	CTSK	cathepsin K
263	91	hsa-miR-106a-5p	RAPH1	Ras association (RalGDS/AF-6) and pleckstrin homology domains 1
264	91	hsa-miR-106a-5p	SEMA4B	semaphorin 4B
265	91	hsa-miR-106a-5p	PPP1R3B	protein phosphatase 1 regulatory subunit 3B
266	91	hsa-miR-106a-5p	NTN4	netrin 4
267	91	hsa-miR-106a-5p	BTBD10	BTB domain containing 10
268	91	hsa-miR-106a-5p	RB1CC1	RB1 inducible coiled-coil 1
269	91	hsa-miR-106a-5p	ARHGAP1	Rho GTPase activating protein 1
270	91	hsa-miR-106a-5p	TET1	tet methylcytosine dioxygenase 1
271	91	hsa-miR-106a-5p	TM2D2	TM2 domain containing 2
272	91	hsa-miR-106a-5p	DENND5B	DENN domain containing 5B
273	91	hsa-miR-106a-5p	SCN1A	sodium voltage-gated channel alpha subunit 1
274	91	hsa-miR-106a-5p	CSRNP3	cysteine and serine rich nuclear protein 3
275	91	hsa-miR-106a-5p	MAPRE3	microtubule associated protein RP/EB family member 3
276	91	hsa-miR-106a-5p	CD69	CD69 molecule
277	91	hsa-miR-106a-5p	ULK1	unc-51 like autophagy activating kinase 1
278	91	hsa-miR-106a-5p	RUNX3	runt related transcription factor 3
279	91	hsa-miR-106a-5p	NBEA	neurobeachin
280	91	hsa-miR-106a-5p	FAM102A	family with sequence similarity 102 member A
281	91	hsa-miR-106a-5p	DAB2	DAB2, clathrin adaptor protein
282	91	hsa-miR-106a-5p	USP28	ubiquitin specific peptidase 28
283	91	hsa-miR-106a-5p	CROT	carnitine O-octanoyltransferase
284	91	hsa-miR-106a-5p	ERAP1	endoplasmic reticulum aminopeptidase 1

285	91	hsa-miR-106a-5p	TNKS2	tankyrase 2
286	91	hsa-miR-106a-5p	CENPQ	centromere protein Q
287	91	hsa-miR-106a-5p	RAB11FIP1	RAB11 family interacting protein 1
288	91	hsa-miR-106a-5p	SESN3	sestrin 3
289	91	hsa-miR-106a-5p	WDFY3	WD repeat and FYVE domain containing 3
290	91	hsa-miR-106a-5p	AMER2	APC membrane recruitment protein 2
291	91	hsa-miR-106a-5p	NRIP3	nuclear receptor interacting protein 3
292	91	hsa-miR-106a-5p	TIAM1	T cell lymphoma invasion and metastasis 1
293	91	hsa-miR-106a-5p	SLAIN2	SLAIN motif family member 2
294	91	hsa-miR-106a-5p	HAUS8	HAUS augmin like complex subunit 8
295	91	hsa-miR-106a-5p	LDLR	low density lipoprotein receptor
296	91	hsa-miR-106a-5p	PCDH15	protocadherin related 15
297	91	hsa-miR-106a-5p	THRA	thyroid hormone receptor alpha
298	91	hsa-miR-106a-5p	LRRC55	leucine rich repeat containing 55
299	91	hsa-miR-106a-5p	SMOC1	SPARC related modular calcium binding 1
300	91	hsa-miR-106a-5p	FAM189A1	family with sequence similarity 189 member A1
301	91	hsa-miR-106a-5p	LRP8	LDL receptor related protein 8
302	91	hsa-miR-106a-5p	LRCH1	leucine rich repeats and calponin homology domain containing 1
303	90	hsa-miR-106a-5p	SLC33A1	solute carrier family 33 member 1
304	90	hsa-miR-106a-5p	ENTPD4	ectonucleoside triphosphate diphosphohydrolase 4
305	90	hsa-miR-106a-5p	S1PR1	sphingosine-1-phosphate receptor 1
306	90	hsa-miR-106a-5p	GNB5	G protein subunit beta 5

307	90	hsa-miR-106a-5p	CCDC71L	coiled-coil domain containing 71 like
308	90	hsa-miR-106a-5p	GOLGA1	golgin A1
309	90	hsa-miR-106a-5p	ETV1	ETS variant 1
310	90	hsa-miR-106a-5p	YOD1	YOD1 deubiquitinase
311	90	hsa-miR-106a-5p	TRPV6	transient receptor potential cation channel subfamily V member 6
312	90	hsa-miR-106a-5p	EGR2	early growth response 2
313	90	hsa-miR-106a-5p	AP2B1	adaptor related protein complex 2 subunit beta 1
314	90	hsa-miR-106a-5p	C7orf43	chromosome 7 open reading frame 43
315	90	hsa-miR-106a-5p	MAPK1	mitogen-activated protein kinase 1
316	90	hsa-miR-106a-5p	MTMR3	myotubularin related protein 3
317	90	hsa-miR-106a-5p	CREB1	cAMP responsive element binding protein 1
318	90	hsa-miR-106a-5p	CAPRIN2	caprin family member 2
319	90	hsa-miR-106a-5p	NACC2	NACC family member 2
320	90	hsa-miR-106a-5p	TGM2	transglutaminase 2
321	90	hsa-miR-106a-5p	CMKLR1	chemerin chemokine-like receptor 1
322	90	hsa-miR-106a-5p	APCDD1	APC down-regulated 1
323	90	hsa-miR-106a-5p	AFG1L	AFG1 like ATPase
324	90	hsa-miR-106a-5p	FAM219B	family with sequence similarity 219 member B
325	90	hsa-miR-106a-5p	NAA30	N(alpha)-acetyltransferase 30, NatC catalytic subunit
326	90	hsa-miR-106a-5p	KPNA2	karyopherin subunit alpha 2
327	90	hsa-miR-106a-5p	RGMB	repulsive guidance molecule BMP co-receptor b
328	90	hsa-miR-106a-5p	HECTD2	HECT domain E3 ubiquitin protein ligase 2
329	90	hsa-miR-106a-5p	PRR15	proline rich 15
330	90	hsa-miR-106a-5p	SSH2	slingshot protein phosphatase 2

331	90	hsa-miR-106a-5p	PLXDC2	plexin domain containing 2
332	90	hsa-miR-106a-5p	MEX3D	mex-3 RNA binding family member D
333	90	hsa-miR-106a-5p	ARHGEF11	Rho guanine nucleotide exchange factor 11
334	90	hsa-miR-106a-5p	SLMAP	sarcolemma associated protein
335	90	hsa-miR-106a-5p	BEST3	bestrophin 3
336	90	hsa-miR-106a-5p	SIKE1	suppressor of IKBKE 1
337	90	hsa-miR-106a-5p	CALD1	caldesmon 1
338	90	hsa-miR-106a-5p	SSX2IP	SSX family member 2 interacting protein
339	90	hsa-miR-106a-5p	AGFG1	ArfGAP with FG repeats 1
340	90	hsa-miR-106a-5p	DPYSL2	dihydropyrimidinase like 2
341	90	hsa-miR-106a-5p	STRIP2	striatin interacting protein 2
342	90	hsa-miR-106a-5p	UBE3C	ubiquitin protein ligase E3C
343	90	hsa-miR-106a-5p	OSTM1	osteoclastogenesis associated transmembrane protein 1
344	89	hsa-miR-106a-5p	DERL2	derlin 2
345	89	hsa-miR-106a-5p	APP	amyloid beta precursor protein
346	89	hsa-miR-106a-5p	CYBRD1	cytochrome b reductase 1
347	89	hsa-miR-106a-5p	ABL2	ABL proto-oncogene 2, non-receptor tyrosine kinase
348	89	hsa-miR-106a-5p	ROCK2	Rho associated coiled-coil containing protein kinase 2
349	89	hsa-miR-106a-5p	SLC4A8	solute carrier family 4 member 8
350	89	hsa-miR-106a-5p	IRF1	interferon regulatory factor 1
351	89	hsa-miR-106a-5p	KIAA1191	KIAA1191
352	89	hsa-miR-106a-5p	OLFM3	olfactomedin 3
353	89	hsa-miR-106a-5p	ZFAND4	zinc finger AN1-type containing 4
354	89	hsa-miR-106a-5p	TMEM64	transmembrane protein 64

355	89	hsa-miR-106a-5p	ZBTB18	zinc finger and BTB domain containing 18
356	89	hsa-miR-106a-5p	CHP2	calcineurin like EF-hand protein 2
357	89	hsa-miR-106a-5p	NIPA1	NIPA magnesium transporter 1
358	89	hsa-miR-106a-5p	LHX6	LIM homeobox 6
359	89	hsa-miR-106a-5p	CHD5	chromodomain helicase DNA binding protein 5
360	89	hsa-miR-106a-5p	SLC16A6	solute carrier family 16 member 6
361	89	hsa-miR-106a-5p	BTBD7	BTB domain containing 7
362	89	hsa-miR-106a-5p	ZBTB33	zinc finger and BTB domain containing 33
363	89	hsa-miR-106a-5p	IL1RAP	interleukin 1 receptor accessory protein
364	89	hsa-miR-106a-5p	RPS6KA4	ribosomal protein S6 kinase A4
365	89	hsa-miR-106a-5p	TNFAIP1	TNF alpha induced protein 1
366	89	hsa-miR-106a-5p	SMOC2	SPARC related modular calcium binding 2
367	89	hsa-miR-106a-5p	CC2D1A	coiled-coil and C2 domain containing 1A
368	89	hsa-miR-106a-5p	SRGAP1	SLIT-ROBO Rho GTPase activating protein 1
369	89	hsa-miR-106a-5p	LYST	lysosomal trafficking regulator
370	89	hsa-miR-106a-5p	TNKS1BP1	tankyrase 1 binding protein 1
371	89	hsa-miR-106a-5p	CERCAM	cerebral endothelial cell adhesion molecule
372	89	hsa-miR-106a-5p	UNC80	unc-80 homolog, NALCN channel complex subunit
373	89	hsa-miR-106a-5p	KLHL15	kelch like family member 15
374	89	hsa-miR-106a-5p	MAP10	microtubule associated protein 10
375	89	hsa-miR-106a-5p	ST6GALNAC6	ST6 N-acetylgalactosaminide alpha-2,6-sialyltransferase 6

376	89	hsa-miR-106a-5p	PITPNA	phosphatidylinositol transfer protein alpha
377	88	hsa-miR-106a-5p	PANX2	pannexin 2
378	88	hsa-miR-106a-5p	FAM19A1	family with sequence similarity 19 member A1, C-C motif chemokine like
379	88	hsa-miR-106a-5p	PGBD5	piggyBac transposable element derived 5
380	88	hsa-miR-106a-5p	KLF11	Kruppel like factor 11
381	88	hsa-miR-106a-5p	UNKL	unk like zinc finger
382	88	hsa-miR-106a-5p	PXK	PX domain containing serine/threonine kinase like
383	88	hsa-miR-106a-5p	ERC1	ELKS/RAB6-interacting/CAST family member 1
384	88	hsa-miR-106a-5p	OSM	oncostatin M
385	88	hsa-miR-106a-5p	FNBP1L	formin binding protein 1 like
386	88	hsa-miR-106a-5p	CXCL6	C-X-C motif chemokine ligand 6
387	88	hsa-miR-106a-5p	HTR2A	5-hydroxytryptamine receptor 2A
388	88	hsa-miR-106a-5p	MOSMO	modulator of smoothened
389	88	hsa-miR-106a-5p	SMAD5	SMAD family member 5
390	88	hsa-miR-106a-5p	TRIM3	tripartite motif containing 3
391	88	hsa-miR-106a-5p	PIK3R1	phosphoinositide-3-kinase regulatory subunit 1
392	88	hsa-miR-106a-5p	CRYBG3	crystallin beta-gamma domain containing 3
393	88	hsa-miR-106a-5p	SFMBT1	Scm like with four mbt domains 1
394	88	hsa-miR-106a-5p	WNK3	WNK lysine deficient protein kinase 3
395	88	hsa-miR-106a-5p	WDR37	WD repeat domain 37
396	88	hsa-miR-106a-5p	SLC16A9	solute carrier family 16 member 9
397	88	hsa-miR-106a-5p	RLIM	ring finger protein, LIM domain interacting

398	88	hsa-miR-106a-5p	OSR1	odd-skipped related transcription factor 1
399	88	hsa-miR-106a-5p	STK38	serine/threonine kinase 38
400	88	hsa-miR-106a-5p	SLC22A23	solute carrier family 22 member 23
401	88	hsa-miR-106a-5p	GOSR1	golgi SNAP receptor complex member 1
402	88	hsa-miR-106a-5p	FBXO31	F-box protein 31
403	88	hsa-miR-106a-5p	OTUD4	OTU deubiquitinase 4
404	88	hsa-miR-106a-5p	CNRIP1	cannabinoid receptor interacting protein 1
405	88	hsa-miR-106a-5p	OXR1	oxidation resistance 1
406	88	hsa-miR-106a-5p	PLAGL2	PLAG1 like zinc finger 2
407	87	hsa-miR-106a-5p	SNX8	sorting nexin 8
408	87	hsa-miR-106a-5p	FAM13A	family with sequence similarity 13 member A
409	87	hsa-miR-106a-5p	AKAP11	A-kinase anchoring protein 11
410	87	hsa-miR-106a-5p	STAT3	signal transducer and activator of transcription 3
411	87	hsa-miR-106a-5p	ARHGEF10	Rho guanine nucleotide exchange factor 10
412	87	hsa-miR-106a-5p	ZNF202	zinc finger protein 202
413	87	hsa-miR-106a-5p	PHIP	pleckstrin homology domain interacting protein
414	87	hsa-miR-106a-5p	HIF1A	hypoxia inducible factor 1 subunit alpha
415	87	hsa-miR-106a-5p	ISM2	isthmin 2
416	87	hsa-miR-106a-5p	ZNF264	zinc finger protein 264
417	87	hsa-miR-106a-5p	CREB5	cAMP responsive element binding protein 5
418	87	hsa-miR-106a-5p	FNDC3B	fibronectin type III domain containing 3B
419	87	hsa-miR-106a-5p	DNAL1	dynein axonemal light chain 1
420	87	hsa-miR-106a-5p	CD274	CD274 molecule
421	87	hsa-miR-106a-5p	BTN3A1	butyrophilin subfamily 3 member A1
422	87	hsa-miR-106a-5p	ORMDL3	ORMDL sphingolipid biosynthesis regulator 3

423	87	hsa-miR-106a-5p	EREG	epiregulin
424	87	hsa-miR-106a-5p	MIDN	midnolin
425	87	hsa-miR-106a-5p	CNOT7	CCR4-NOT transcription complex subunit 7
426	87	hsa-miR-106a-5p	FEM1C	fem-1 homolog C
427	87	hsa-miR-106a-5p	SH3PXD2A	SH3 and PX domains 2A
428	87	hsa-miR-106a-5p	BAHD1	bromo adjacent homology domain containing 1
429	87	hsa-miR-106a-5p	ST3GAL1	ST3 beta-galactoside alpha-2,3-sialyltransferase 1
430	87	hsa-miR-106a-5p	USP32	ubiquitin specific peptidase 32
431	87	hsa-miR-106a-5p	ZNF148	zinc finger protein 148
432	87	hsa-miR-106a-5p	ANKRD50	ankyrin repeat domain 50
433	87	hsa-miR-106a-5p	ZNF236	zinc finger protein 236
434	87	hsa-miR-106a-5p	PPP1R21	protein phosphatase 1 regulatory subunit 21
435	86	hsa-miR-106a-5p	CEP170	centrosomal protein 170
436	86	hsa-miR-106a-5p	RAPGEFL1	Rap guanine nucleotide exchange factor like 1
437	86	hsa-miR-106a-5p	TGFB1I1	transforming growth factor beta 1 induced transcript 1
438	86	hsa-miR-106a-5p	SERF1B	small EDRK-rich factor 1B
439	86	hsa-miR-106a-5p	TFAM	transcription factor A, mitochondrial
440	86	hsa-miR-106a-5p	BICC1	BicC family RNA binding protein 1
441	86	hsa-miR-106a-5p	MFAP3L	microfibril associated protein 3 like
442	86	hsa-miR-106a-5p	37500	septin 2
443	86	hsa-miR-106a-5p	PLXNA1	plexin A1
444	86	hsa-miR-106a-5p	KIF23	kinesin family member 23
445	86	hsa-miR-106a-5p	SOX4	SRY-box 4
446	86	hsa-miR-106a-5p	ABI1	abl interactor 1
447	86	hsa-miR-106a-5p	RAB10	RAB10, member RAS oncogene family

448	86	hsa-miR-106a-5p	CRY2	cryptochrome circadian regulator 2
449	86	hsa-miR-106a-5p	P2RX4	purinergic receptor P2X 4
450	86	hsa-miR-106a-5p	ATP12A	ATPase H+/K+ transporting non-gastric alpha2 subunit
451	86	hsa-miR-106a-5p	ZSCAN20	zinc finger and SCAN domain containing 20
452	86	hsa-miR-106a-5p	ANKRD13C	ankyrin repeat domain 13C
453	86	hsa-miR-106a-5p	TSG101	tumor susceptibility 101
454	86	hsa-miR-106a-5p	FAT4	FAT atypical cadherin 4
455	86	hsa-miR-106a-5p	PDGFRA	platelet derived growth factor receptor alpha
456	86	hsa-miR-106a-5p	ARHGEF18	Rho/Rac guanine nucleotide exchange factor 18
457	86	hsa-miR-106a-5p	TANC1	tetratricopeptide repeat, ankyrin repeat and coiled-coil containing 1
458	86	hsa-miR-106a-5p	MINK1	misshapen like kinase 1
459	86	hsa-miR-106a-5p	CMPK1	cytidine/uridine monophosphate kinase 1
460	86	hsa-miR-106a-5p	RBM12B	RNA binding motif protein 12B
461	86	hsa-miR-106a-5p	DOCK4	dedicator of cytokinesis 4
462	86	hsa-miR-106a-5p	GPATCH2	G-patch domain containing 2
463	86	hsa-miR-106a-5p	AGFG2	ArfGAP with FG repeats 2
464	86	hsa-miR-106a-5p	SQSTM1	sequestosome 1
465	86	hsa-miR-106a-5p	RBBP7	RB binding protein 7, chromatin remodeling factor
466	86	hsa-miR-106a-5p	LMO3	LIM domain only 3
467	86	hsa-miR-106a-5p	SGTB	small glutamine rich tetratricopeptide repeat containing beta
468	86	hsa-miR-106a-5p	SERF1A	small EDRK-rich factor 1A
469	86	hsa-miR-106a-5p	ABCG4	ATP binding cassette subfamily G member 4

470	86	hsa-miR-106a-5p	FAM210A	family with sequence similarity 210 member A
471	86	hsa-miR-106a-5p	PARD6B	par-6 family cell polarity regulator beta
472	86	hsa-miR-106a-5p	RAB8B	RAB8B, member RAS oncogene family
473	86	hsa-miR-106a-5p	IL6ST	interleukin 6 signal transducer
474	86	hsa-miR-106a-5p	TPRG1L	tumor protein p63 regulated 1 like
475	86	hsa-miR-106a-5p	WEE1	WEE1 G2 checkpoint kinase
476	86	hsa-miR-106a-5p	MCL1	MCL1, BCL2 family apoptosis regulator
477	86	hsa-miR-106a-5p	FBXL3	F-box and leucine rich repeat protein 3
478	86	hsa-miR-106a-5p	GNS	glucosamine (N-acetyl)-6-sulfatase
479	86	hsa-miR-106a-5p	PXYLP1	2-phosphoxylose phosphatase 1
480	86	hsa-miR-106a-5p	FAM199X	family with sequence similarity 199, X-linked
481	86	hsa-miR-106a-5p	C14orf28	chromosome 14 open reading frame 28
482	86	hsa-miR-106a-5p	MAP3K8	mitogen-activated protein kinase kinase kinase 8
483	86	hsa-miR-106a-5p	SCAMP5	secretory carrier membrane protein 5
484	85	hsa-miR-106a-5p	PLAG1	PLAG1 zinc finger
485	85	hsa-miR-106a-5p	PSG3	pregnancy specific beta-1-glycoprotein 3
486	85	hsa-miR-106a-5p	TSPAN9	tetraspanin 9
487	85	hsa-miR-106a-5p	RNF6	ring finger protein 6
488	85	hsa-miR-106a-5p	CORO2B	coronin 2B
489	85	hsa-miR-106a-5p	REV3L	REV3 like, DNA directed polymerase zeta catalytic subunit
490	85	hsa-miR-106a-5p	NR2C1	nuclear receptor subfamily 2 group C member 1
491	85	hsa-miR-106a-5p	FBXO21	F-box protein 21
492	85	hsa-miR-106a-5p	ABHD5	abhydrolase domain containing 5

493	85	hsa-miR-106a-5p	MYLIP	myosin regulatory light chain interacting protein
494	85	hsa-miR-106a-5p	UXS1	UDP-glucuronate decarbox-ylase 1
495	85	hsa-miR-106a-5p	UNK	unk zinc finger
496	85	hsa-miR-106a-5p	ZBTB8A	zinc finger and BTB domain containing 8A
497	85	hsa-miR-106a-5p	GUCY1A1	guanylate cyclase 1 soluble subunit alpha 1
498	85	hsa-miR-106a-5p	WDFY2	WD repeat and FYVE domain containing 2
499	85	hsa-miR-106a-5p	ARAP2	ArfGAP with RhoGAP domain, ankyrin repeat and PH domain 2
500	85	hsa-miR-106a-5p	MAPK4	mitogen-activated protein ki-nase 4
501	85	hsa-miR-106a-5p	LYPD6	LY6/PLAUR domain contain-ing 6
502	85	hsa-miR-106a-5p	TMEM100	transmembrane protein 100
503	85	hsa-miR-106a-5p	RGMA	repulsive guidance molecule BMP co-receptor a
504	85	hsa-miR-106a-5p	PSD	pleckstrin and Sec7 domain containing
505	85	hsa-miR-106a-5p	CNOT6	CCR4-NOT transcription com-plex subunit 6
506	85	hsa-miR-106a-5p	KIF26B	kinesin family member 26B
507	85	hsa-miR-106a-5p	SLC49A4	solute carrier family 49 member 4
508	85	hsa-miR-106a-5p	MMP24	matrix metallopeptidase 24
509	85	hsa-miR-106a-5p	NUP35	nucleoporin 35
510	85	hsa-miR-106a-5p	REPS2	RALBP1 associated Eps do-main containing 2
511	85	hsa-miR-106a-5p	LCOR	ligand dependent nuclear recep-tor corepressor
512	85	hsa-miR-106a-5p	PBX3	PBX homeobox 3
513	85	hsa-miR-106a-5p	EPS15L1	epidermal growth factor recep-tor pathway substrate 15 like 1

514	84	hsa-miR-106a-5p	WFS1	wolframin ER transmembrane glycoprotein
515	84	hsa-miR-106a-5p	TMX3	thioredoxin related transmem-brane protein 3
516	84	hsa-miR-106a-5p	FOXK2	forkhead box K2
517	84	hsa-miR-106a-5p	ATXN7L1	ataxin 7 like 1
518	84	hsa-miR-106a-5p	ATXN1	ataxin 1
519	84	hsa-miR-106a-5p	MFN2	mitofusin 2
520	84	hsa-miR-106a-5p	TMEM265	transmembrane protein 265
521	84	hsa-miR-106a-5p	FASTK	Fas activated serine/threonine kinase
522	84	hsa-miR-106a-5p	SOCS6	suppressor of cytokine signal-ing 6
523	84	hsa-miR-106a-5p	RAB12	RAB12, member RAS onco-gene family
524	84	hsa-miR-106a-5p	TBC1D8B	TBC1 domain family member 8B
525	84	hsa-miR-106a-5p	TMEM167A	transmembrane protein 167A
526	84	hsa-miR-106a-5p	TENM1	teneurin transmembrane protein 1
527	84	hsa-miR-106a-5p	RETREG3	reticulophagy regulator family member 3
528	84	hsa-miR-106a-5p	BHLHE41	basic helix-loop-helix family member e41
529	84	hsa-miR-106a-5p	PTPN21	protein tyrosine phosphatase, non-receptor type 21
530	84	hsa-miR-106a-5p	BICD2	BICD cargo adaptor 2
531	84	hsa-miR-106a-5p	SYTL4	synaptotagmin like 4
532	84	hsa-miR-106a-5p	AKAP13	A-kinase anchoring protein 13
533	84	hsa-miR-106a-5p	DUSP8	dual specificity phosphatase 8
534	84	hsa-miR-106a-5p	ARHGEF3	Rho guanine nucleotide ex-change factor 3
535	84	hsa-miR-106a-5p	RHOC	ras homolog family member C
536	84	hsa-miR-106a-5p	PRR16	proline rich 16
537	84	hsa-miR-106a-5p	NEUROG1	neurogenin 1
538	84	hsa-miR-106a-5p	TAGAP	T cell activation RhoGTPase activating protein

539	84	hsa-miR-106a-5p	MAP7	microtubule associated protein 7
540	84	hsa-miR-106a-5p	RSRP1	arginine and serine rich protein 1
541	83	hsa-miR-106a-5p	PAPOLB	poly(A) polymerase beta
542	83	hsa-miR-106a-5p	SCN2B	sodium voltage-gated channel beta subunit 2
543	83	hsa-miR-106a-5p	TRIM37	tripartite motif containing 37
544	83	hsa-miR-106a-5p	PDLIM5	PDZ and LIM domain 5
545	83	hsa-miR-106a-5p	MAGI3	membrane associated guanylate kinase, WW and PDZ domain containing 3
546	83	hsa-miR-106a-5p	SH3BP5	SH3 domain binding protein 5
547	83	hsa-miR-106a-5p	ARHGEF28	Rho guanine nucleotide exchange factor 28
548	83	hsa-miR-106a-5p	DDX5	DEAD-box helicase 5
549	83	hsa-miR-106a-5p	SRPK2	SRSF protein kinase 2
550	83	hsa-miR-106a-5p	DCUN1D1	defective in cullin neddylation 1 domain containing 1
551	83	hsa-miR-106a-5p	CTSA	cathepsin A
552	83	hsa-miR-106a-5p	RNH1	ribonuclease/angiogenin inhibitor 1
553	83	hsa-miR-106a-5p	CAPN15	calpain 15
554	83	hsa-miR-106a-5p	LRPAP1	LDL receptor related protein associated protein 1
555	83	hsa-miR-106a-5p	NFIC	nuclear factor I C
556	83	hsa-miR-106a-5p	ZDHHC1	zinc finger DHHC-type containing 1
557	83	hsa-miR-106a-5p	PFKFB3	6-phosphofructo-2-kinase/fructose-2,6-biphosphatase 3
558	83	hsa-miR-106a-5p	SMAD4	SMAD family member 4
559	83	hsa-miR-106a-5p	C9orf40	chromosome 9 open reading frame 40
560	83	hsa-miR-106a-5p	GRAMD1A	GRAM domain containing 1A
561	83	hsa-miR-106a-5p	ZHX2	zinc fingers and homeoboxes 2
562	83	hsa-miR-106a-5p	MYNN	myoneurin

563	83	hsa-miR-106a-5p	RNASEH2B	ribonuclease H2 subunit B
564	83	hsa-miR-106a-5p	FLT1	fms related tyrosine kinase 1
565	83	hsa-miR-106a-5p	SNX16	sorting nexin 16
566	83	hsa-miR-106a-5p	KCNJ10	potassium voltage-gated channel subfamily J member 10
567	83	hsa-miR-106a-5p	C2orf69	chromosome 2 open reading frame 69
568	83	hsa-miR-106a-5p	MCF2L	MCF.2 cell line derived transforming sequence like
569	83	hsa-miR-106a-5p	ZBTB21	zinc finger and BTB domain containing 21
570	83	hsa-miR-106a-5p	HLF	HLF, PAR bZIP transcription factor
571	83	hsa-miR-106a-5p	RETREG2	reticulophagy regulator family member 2
572	83	hsa-miR-106a-5p	39508	membrane associated ring-CH-type finger 8
573	83	hsa-miR-106a-5p	RBL1	RB transcriptional corepressor like 1
574	83	hsa-miR-106a-5p	SCAMP2	secretory carrier membrane protein 2
575	83	hsa-miR-106a-5p	STK11	serine/threonine kinase 11
576	83	hsa-miR-106a-5p	CDC23	cell division cycle 23
577	83	hsa-miR-106a-5p	ZFPM2	zinc finger protein, FOG family member 2
578	82	hsa-miR-106a-5p	ATG2B	autophagy related 2B
579	82	hsa-miR-106a-5p	SERP1	stress associated endoplasmic reticulum protein 1
580	82	hsa-miR-106a-5p	PLCB1	phospholipase C beta 1
581	82	hsa-miR-106a-5p	CNOT6L	CCR4-NOT transcription complex subunit 6 like
582	82	hsa-miR-106a-5p	CDC37L1	cell division cycle 37 like 1
583	82	hsa-miR-106a-5p	CDKN1A	cyclin dependent kinase inhibitor 1A
584	82	hsa-miR-106a-5p	USP6	ubiquitin specific peptidase 6

585	82	hsa-miR-106a-5p	PTPDC1	protein tyrosine phosphatase domain containing 1
586	82	hsa-miR-106a-5p	NFIB	nuclear factor I B
587	82	hsa-miR-106a-5p	ZDHHC9	zinc finger DHHC-type containing 9
588	82	hsa-miR-106a-5p	KATNAL1	katanin catalytic subunit A1 like 1
589	82	hsa-miR-106a-5p	SORL1	sortilin related receptor 1
590	82	hsa-miR-106a-5p	RRAS2	RAS related 2
591	82	hsa-miR-106a-5p	PRCP	prolylcarboxypeptidase
592	82	hsa-miR-106a-5p	PRR14L	proline rich 14 like
593	82	hsa-miR-106a-5p	PAG1	phosphoprotein membrane anchor with glycosphingolipid microdomains 1
594	82	hsa-miR-106a-5p	DNAJB9	DnaJ heat shock protein family (Hsp40) member B9
595	82	hsa-miR-106a-5p	MMP2	matrix metallopeptidase 2
596	82	hsa-miR-106a-5p	NTNG1	netrin G1
597	82	hsa-miR-106a-5p	NANOS1	nanos C2HC-type zinc finger 1
598	82	hsa-miR-106a-5p	MAPK8	mitogen-activated protein kinase 8
599	82	hsa-miR-106a-5p	USP24	ubiquitin specific peptidase 24
600	82	hsa-miR-106a-5p	PAPOLA	poly(A) polymerase alpha
601	82	hsa-miR-106a-5p	NEUROG2	neurogenin 2
602	82	hsa-miR-106a-5p	SPTY2D1	SPT2 chromatin protein domain containing 1
603	82	hsa-miR-106a-5p	ZNF280B	zinc finger protein 280B
604	82	hsa-miR-106a-5p	MYO5B	myosin VB
605	82	hsa-miR-106a-5p	TMEM138	transmembrane protein 138
606	81	hsa-miR-106a-5p	ZNF25	zinc finger protein 25
607	81	hsa-miR-106a-5p	NPLOC4	NPL4 homolog, ubiquitin recognition factor
608	81	hsa-miR-106a-5p	SAMD8	sterile alpha motif domain containing 8
609	81	hsa-miR-106a-5p	TRDN	triadin

610	81	hsa-miR-106a-5p	EIF4A2	eukaryotic translation initiation factor 4A2
611	81	hsa-miR-106a-5p	TMBIM6	transmembrane BAX inhibitor motif containing 6
612	81	hsa-miR-106a-5p	KMT5B	lysine methyltransferase 5B
613	81	hsa-miR-106a-5p	MTF1	metal regulatory transcription factor 1
614	81	hsa-miR-106a-5p	ABHD2	abhydrolase domain containing 2
615	81	hsa-miR-106a-5p	ZNF597	zinc finger protein 597
616	81	hsa-miR-106a-5p	GABBR2	gamma-aminobutyric acid type B receptor subunit 2
617	81	hsa-miR-106a-5p	KLF9	Kruppel like factor 9
618	81	hsa-miR-106a-5p	RCCD1	RCC1 domain containing 1
619	81	hsa-miR-106a-5p	SLC4A4	solute carrier family 4 member 4
620	81	hsa-miR-106a-5p	RPS6KA6	ribosomal protein S6 kinase A6
621	81	hsa-miR-106a-5p	PPP3R1	protein phosphatase 3 regulatory subunit B, alpha
622	81	hsa-miR-106a-5p	AHNAK	AHNAK nucleoprotein
623	81	hsa-miR-106a-5p	RAB30	RAB30, member RAS oncogene family
624	81	hsa-miR-106a-5p	KLF12	Kruppel like factor 12
625	81	hsa-miR-106a-5p	NDEL1	nudE neurodevelopment protein 1 like 1
626	81	hsa-miR-106a-5p	ZBTB9	zinc finger and BTB domain containing 9
627	81	hsa-miR-106a-5p	NPAS3	neuronal PAS domain protein 3
628	81	hsa-miR-106a-5p	TP73	tumor protein p73
629	81	hsa-miR-106a-5p	GNPDA2	glucosamine-6-phosphate deaminase 2
630	81	hsa-miR-106a-5p	FAM13C	family with sequence similarity 13 member C
631	81	hsa-miR-106a-5p	CPEB3	cytoplasmic polyadenylation element binding protein 3
632	81	hsa-miR-106a-5p	NHLRC3	NHL repeat containing 3

633	80	hsa-miR-106a-5p	PKD1	polycystin 1, transient receptor potential channel interacting
634	80	hsa-miR-106a-5p	FAM3C	family with sequence similarity 3 member C
635	80	hsa-miR-106a-5p	RNF217	ring finger protein 217
636	80	hsa-miR-106a-5p	STYX	serine/threonine/tyrosine interacting protein
637	80	hsa-miR-106a-5p	MSR1	macrophage scavenger receptor 1
638	80	hsa-miR-106a-5p	ZNF2	zinc finger protein 2
639	80	hsa-miR-106a-5p	SLC30A7	solute carrier family 30 member 7
640	80	hsa-miR-106a-5p	SS18L1	SS18L1, nBAF chromatin remodeling complex subunit
641	80	hsa-miR-106a-5p	JAK1	Janus kinase 1
642	80	hsa-miR-106a-5p	ATG14	autophagy related 14
643	80	hsa-miR-106a-5p	SLC41A1	solute carrier family 41 member 1
644	80	hsa-miR-106a-5p	SCN2A	sodium voltage-gated channel alpha subunit 2
645	80	hsa-miR-106a-5p	MDM4	MDM4, p53 regulator
646	80	hsa-miR-106a-5p	LZIC	leucine zipper and CTNNBIP1 domain containing
647	80	hsa-miR-106a-5p	SLC45A4	solute carrier family 45 member 4
648	80	hsa-miR-106a-5p	AKT3	AKT serine/threonine kinase 3
649	80	hsa-miR-106a-5p	TAOK1	TAO kinase 1
650	80	hsa-miR-106a-5p	BMT2	base methyltransferase of 25S rRNA 2 homolog
651	80	hsa-miR-106a-5p	BCL2L11	BCL2 like 11
652	80	hsa-miR-106a-5p	KIAA1522	KIAA1522
653	80	hsa-miR-106a-5p	PTGDR	prostaglandin D2 receptor
654	80	hsa-miR-106a-5p	VASP	vasodilator stimulated phosphoprotein
655	80	hsa-miR-106a-5p	AMIGO2	adhesion molecule with Ig like domain 2
656	80	hsa-miR-106a-5p	BAMBI	BMP and activin membrane bound inhibitor

657	79	hsa-miR-106a-5p	CBLN4	cerebellin 4 precursor
658	79	hsa-miR-106a-5p	ABCD2	ATP binding cassette subfamily D member 2
659	79	hsa-miR-106a-5p	SPRY4	sprouty RTK signaling antagonist 4
660	79	hsa-miR-106a-5p	TWF1	twinfilin actin binding protein 1
661	79	hsa-miR-106a-5p	KPNA3	karyopherin subunit alpha 3
662	79	hsa-miR-106a-5p	OSBPL5	oxysterol binding protein like 5
663	79	hsa-miR-106a-5p	ZBTB47	zinc finger and BTB domain containing 47
664	79	hsa-miR-106a-5p	IKZF4	IKAROS family zinc finger 4
665	79	hsa-miR-106a-5p	CXCL14	C-X-C motif chemokine ligand 14
666	79	hsa-miR-106a-5p	PPARA	peroxisome proliferator activated receptor alpha
667	79	hsa-miR-106a-5p	MCHR2	melanin concentrating hormone receptor 2
668	79	hsa-miR-106a-5p	PAFAH1B1	platelet activating factor acetylhydrolase 1b regulatory subunit 1
669	79	hsa-miR-106a-5p	PHLPP2	PH domain and leucine rich repeat protein phosphatase 2
670	79	hsa-miR-106a-5p	ZNF362	zinc finger protein 362
671	79	hsa-miR-106a-5p	WWP2	WW domain containing E3 ubiquitin protein ligase 2
672	79	hsa-miR-106a-5p	ITCH	itchy E3 ubiquitin protein ligase
673	79	hsa-miR-106a-5p	MTURN	maturin, neural progenitor differentiation regulator homolog
674	79	hsa-miR-106a-5p	FGL2	fibrinogen like 2
675	79	hsa-miR-106a-5p	MYO1D	myosin ID
676	79	hsa-miR-106a-5p	FEM1B	fem-1 homolog B
677	79	hsa-miR-106a-5p	C6orf120	chromosome 6 open reading frame 120
678	79	hsa-miR-106a-5p	C3orf70	chromosome 3 open reading frame 70

679	78	hsa-miR-106a-5p	TBC1D15	TBC1 domain family member 15
680	78	hsa-miR-106a-5p	ESR1	estrogen receptor 1
681	78	hsa-miR-106a-5p	ERI1	exoribonuclease 1
682	78	hsa-miR-106a-5p	PAF1	PAF1 homolog, Paf1/RNA polymerase II complex component
683	78	hsa-miR-106a-5p	GBF1	golgi brefeldin A resistant guanine nucleotide exchange factor 1
684	78	hsa-miR-106a-5p	RBSN	rabenosyn, RAB effector
685	78	hsa-miR-106a-5p	SMIM14	small integral membrane protein 14
686	78	hsa-miR-106a-5p	NR4A3	nuclear receptor subfamily 4 group A member 3
687	78	hsa-miR-106a-5p	38777	membrane associated ring-CH-type finger 6
688	78	hsa-miR-106a-5p	CIT	citron rho-interacting serine/threonine kinase
689	78	hsa-miR-106a-5p	EEA1	early endosome antigen 1
690	78	hsa-miR-106a-5p	DLGAP1	DLG associated protein 1
691	78	hsa-miR-106a-5p	PTGFRN	prostaglandin F2 receptor inhibitor
692	78	hsa-miR-106a-5p	CHAF1A	chromatin assembly factor 1 subunit A
693	78	hsa-miR-106a-5p	TENT5C	terminal nucleotidyltransferase 5C
694	78	hsa-miR-106a-5p	SHTN1	shootin 1
695	78	hsa-miR-106a-5p	APBB2	amyloid beta precursor protein binding family B member 2
696	78	hsa-miR-106a-5p	UBE2D1	ubiquitin conjugating enzyme E2 D1
697	78	hsa-miR-106a-5p	PAFAH1B2	platelet activating factor acetylhydrolase 1b catalytic subunit 2
698	78	hsa-miR-106a-5p	PLEKHO2	pleckstrin homology domain containing O2
699	78	hsa-miR-106a-5p	POLR3G	RNA polymerase III subunit G

700	78	hsa-miR-106a-5p	ST6GAL-NAC3	ST6 N-acetylgalactosaminide alpha-2,6-sialyltransferase 3
701	78	hsa-miR-106a-5p	CELSR2	cadherin EGF LAG seven-pass G-type receptor 2
702	78	hsa-miR-106a-5p	KRT10	keratin 10
703	78	hsa-miR-106a-5p	COX7A2L	cytochrome c oxidase subunit 7A2 like
704	78	hsa-miR-106a-5p	M6PR	mannose-6-phosphate receptor, cation dependent
705	78	hsa-miR-106a-5p	SHANK2	SH3 and multiple ankyrin repeat domains 2
706	78	hsa-miR-106a-5p	LGI2	leucine rich repeat LGI family member 2
707	78	hsa-miR-106a-5p	SLITRK2	SLIT and NTRK like family member 2
708	78	hsa-miR-106a-5p	NEURL1B	neuralized E3 ubiquitin protein ligase 1B
709	78	hsa-miR-106a-5p	DDIAS	DNA damage induced apoptosis suppressor
710	78	hsa-miR-106a-5p	UTP23	UTP23, small subunit processome component
711	77	hsa-miR-106a-5p	ACBD5	acyl-CoA binding domain containing 5
712	77	hsa-miR-106a-5p	EIF4G2	eukaryotic translation initiation factor 4 gamma 2
713	77	hsa-miR-106a-5p	FAM117A	family with sequence similarity 117 member A
714	77	hsa-miR-106a-5p	CCSAP	centriole, cilia and spindle associated protein
715	77	hsa-miR-106a-5p	SOWAHC	sosondowah ankyrin repeat domain family member C
716	77	hsa-miR-106a-5p	E2F2	E2F transcription factor 2
717	77	hsa-miR-106a-5p	PHTF2	putative homeodomain transcription factor 2
718	77	hsa-miR-106a-5p	RB1	RB transcriptional corepressor 1

719	77	hsa-miR-106a-5p	C3orf38	chromosome 3 open reading frame 38
720	77	hsa-miR-106a-5p	UTP14C	UTP14C, small subunit processome component
721	77	hsa-miR-106a-5p	MAP3K20	mitogen-activated protein kinase kinase kinase 20
722	77	hsa-miR-106a-5p	C16orf70	chromosome 16 open reading frame 70
723	77	hsa-miR-106a-5p	CRIM1	cysteine rich transmembrane BMP regulator 1
724	77	hsa-miR-106a-5p	PTH	parathyroid hormone
725	77	hsa-miR-106a-5p	SUMF1	sulfatase modifying factor 1
726	77	hsa-miR-106a-5p	TRAF4	TNF receptor associated factor 4
727	76	hsa-miR-106a-5p	HMGA2	high mobility group AT-hook 2
728	76	hsa-miR-106a-5p	HOOK3	hook microtubule tethering protein 3
729	76	hsa-miR-106a-5p	RASA2	RAS p21 protein activator 2
730	76	hsa-miR-106a-5p	CRIPT	CXXC repeat containing interactor of PDZ3 domain
731	76	hsa-miR-106a-5p	GABPB1	GA binding protein transcription factor subunit beta 1
732	76	hsa-miR-106a-5p	CREBRF	CREB3 regulatory factor
733	76	hsa-miR-106a-5p	LONP2	lon peptidase 2, peroxisomal
734	76	hsa-miR-106a-5p	BTG2	BTG anti-proliferation factor 2
735	76	hsa-miR-106a-5p	TMEM50B	transmembrane protein 50B
736	76	hsa-miR-106a-5p	ADAT2	adenosine deaminase, tRNA specific 2
737	76	hsa-miR-106a-5p	KDM2A	lysine demethylase 2A
738	76	hsa-miR-106a-5p	PDRG1	p53 and DNA damage regulated 1
739	76	hsa-miR-106a-5p	NMUR2	neuromedin U receptor 2
740	76	hsa-miR-106a-5p	GIGYF1	GRB10 interacting GYF protein 1
741	76	hsa-miR-106a-5p	STX6	syntaxin 6

742	76	hsa-miR-106a-5p	TUSC2	tumor suppressor 2, mitochondrial calcium regulator
743	76	hsa-miR-106a-5p	ATP1A2	ATPase Na+/K+ transporting subunit alpha 2
744	76	hsa-miR-106a-5p	JAZF1	JAZF zinc finger 1
745	76	hsa-miR-106a-5p	ARSJ	arylsulfatase family member J
746	76	hsa-miR-106a-5p	KLHL20	kelch like family member 20
747	76	hsa-miR-106a-5p	PGP	phosphoglycolate phosphatase
748	76	hsa-miR-106a-5p	NRBP1	nuclear receptor binding protein 1
749	76	hsa-miR-106a-5p	ARL4C	ADP ribosylation factor like GTPase 4C
750	76	hsa-miR-106a-5p	DMTF1	cyclin D binding myb like transcription factor 1
751	76	hsa-miR-106a-5p	DSG4	desmoglein 4
752	76	hsa-miR-106a-5p	ATG7	autophagy related 7
753	76	hsa-miR-106a-5p	PREX1	phosphatidylinositol-3,4,5-trisphosphate dependent Rac exchange factor 1
754	76	hsa-miR-106a-5p	PON2	paraoxonase 2
755	76	hsa-miR-106a-5p	PLS1	plastin 1
756	76	hsa-miR-106a-5p	FRMD4B	FERM domain containing 4B
757	76	hsa-miR-106a-5p	PPP6R2	protein phosphatase 6 regulatory subunit 2
758	76	hsa-miR-106a-5p	RABEP1	rabaptin, RAB GTPase binding effector protein 1
759	75	hsa-miR-106a-5p	ST8SIA2	ST8 alpha-N-acetyl-neuraminide alpha-2,8-sialyltransferase 2
760	75	hsa-miR-106a-5p	RAPGEF4	Rap guanine nucleotide exchange factor 4
761	75	hsa-miR-106a-5p	RNF2	ring finger protein 2
762	75	hsa-miR-106a-5p	COMMD6	COMM domain containing 6
763	75	hsa-miR-106a-5p	SKIL	SKI like proto-oncogene

764	75	hsa-miR-106a-5p	MAPRE1	microtubule associated protein RP/EB family member 1
765	75	hsa-miR-106a-5p	KIF5A	kinesin family member 5A
766	75	hsa-miR-106a-5p	RASGEF1A	RasGEF domain family member 1A
767	75	hsa-miR-106a-5p	KLF10	Kruppel like factor 10
768	75	hsa-miR-106a-5p	CCSER2	coiled-coil serine rich protein 2
769	75	hsa-miR-106a-5p	CRK	CRK proto-oncogene, adaptor protein
770	75	hsa-miR-106a-5p	MAP3K12	mitogen-activated protein kinase kinase kinase 12
771	75	hsa-miR-106a-5p	SHOC2	SHOC2, leucine rich repeat scaffold protein
772	75	hsa-miR-106a-5p	TXLNA	taxilin alpha
773	75	hsa-miR-106a-5p	NR4A2	nuclear receptor subfamily 4 group A member 2
774	75	hsa-miR-106a-5p	ALX4	ALX homeobox 4
775	75	hsa-miR-106a-5p	UBE2J1	ubiquitin conjugating enzyme E2 J1
776	75	hsa-miR-106a-5p	TMEM25	transmembrane protein 25
777	75	hsa-miR-106a-5p	TBC1D17	TBC1 domain family member 17
778	75	hsa-miR-106a-5p	AKAP5	A-kinase anchoring protein 5
779	75	hsa-miR-106a-5p	FAS	Fas cell surface death receptor
780	75	hsa-miR-106a-5p	TASOR	transcription activation suppressor
781	75	hsa-miR-106a-5p	IL25	interleukin 25
782	75	hsa-miR-106a-5p	SYT17	synaptotagmin 17
783	75	hsa-miR-106a-5p	LRP1B	LDL receptor related protein 1B
784	75	hsa-miR-106a-5p	KANSL1L	KAT8 regulatory NSL complex subunit 1 like
785	75	hsa-miR-106a-5p	PPP4R3B	protein phosphatase 4 regulatory subunit 3B
786	75	hsa-miR-106a-5p	VCPKMT	valosin containing protein lysine methyltransferase

787	74	hsa-miR-106a-5p	CLEC12B	C-type lectin domain family 12 member B
788	74	hsa-miR-106a-5p	FAM53A	family with sequence similarity 53 member A
789	74	hsa-miR-106a-5p	TMEM267	transmembrane protein 267
790	74	hsa-miR-106a-5p	DLEC1	DLEC1, cilia and flagella associated protein
791	74	hsa-miR-106a-5p	IPO9	importin 9
792	74	hsa-miR-106a-5p	DOK6	docking protein 6
793	74	hsa-miR-106a-5p	ZNF805	zinc finger protein 805
794	74	hsa-miR-106a-5p	RNF11	ring finger protein 11
795	74	hsa-miR-106a-5p	ZADH2	zinc binding alcohol dehydrogenase domain containing 2
796	74	hsa-miR-106a-5p	RPS6KA1	ribosomal protein S6 kinase A1
797	74	hsa-miR-106a-5p	PKN2	protein kinase N2
798	74	hsa-miR-106a-5p	COX8C	cytochrome c oxidase subunit 8C
799	74	hsa-miR-106a-5p	SESN2	sestrin 2
800	74	hsa-miR-106a-5p	MSTO1	misato mitochondrial distribution and morphology regulator 1
801	74	hsa-miR-106a-5p	MAPK9	mitogen-activated protein kinase 9
802	74	hsa-miR-106a-5p	GPC6	glypican 6
803	74	hsa-miR-106a-5p	PRRX1	paired related homeobox 1
804	74	hsa-miR-106a-5p	SLC16A7	solute carrier family 16 member 7
805	73	hsa-miR-106a-5p	PFN2	profilin 2
806	73	hsa-miR-106a-5p	CAMK2N2	calcium/calmodulin dependent protein kinase II inhibitor 2
807	73	hsa-miR-106a-5p	TSHZ3	teashirt zinc finger homeobox 3
808	73	hsa-miR-106a-5p	F2R	coagulation factor II thrombin receptor
809	73	hsa-miR-106a-5p	RNF38	ring finger protein 38
810	73	hsa-miR-106a-5p	LHX8	LIM homeobox 8
811	73	hsa-miR-106a-5p	GID4	GID complex subunit 4 homolog

812	73	hsa-miR-106a-5p	ZBTB6	zinc finger and BTB domain containing 6
813	73	hsa-miR-106a-5p	UBE2B	ubiquitin conjugating enzyme E2 B
814	73	hsa-miR-106a-5p	PRICKLE3	prickle planar cell polarity protein 3
815	73	hsa-miR-106a-5p	SLC29A2	solute carrier family 29 member 2
816	73	hsa-miR-106a-5p	TADA2B	transcriptional adaptor 2B
817	72	hsa-miR-106a-5p	HMGB3	high mobility group box 3
818	72	hsa-miR-106a-5p	CACUL1	CDK2 associated cullin domain 1
819	72	hsa-miR-106a-5p	SH2D5	SH2 domain containing 5
820	72	hsa-miR-106a-5p	PTPRJ	protein tyrosine phosphatase, receptor type J
821	72	hsa-miR-106a-5p	GON4L	gon-4 like
822	72	hsa-miR-106a-5p	EGLN1	egl-9 family hypoxia inducible factor 1
823	72	hsa-miR-106a-5p	POLQ	DNA polymerase theta
824	72	hsa-miR-106a-5p	GINS4	GINS complex subunit 4
825	72	hsa-miR-106a-5p	COL4A3	collagen type IV alpha 3 chain
826	72	hsa-miR-106a-5p	NRP2	neuropilin 2
827	72	hsa-miR-106a-5p	TP53INP1	tumor protein p53 inducible nuclear protein 1
828	72	hsa-miR-106a-5p	MAT2B	methionine adenosyltransferase 2B
829	72	hsa-miR-106a-5p	CAAP1	caspase activity and apoptosis inhibitor 1
830	72	hsa-miR-106a-5p	RNF13	ring finger protein 13
831	72	hsa-miR-106a-5p	ITFG1	integrin alpha FG-GAP repeat containing 1
832	72	hsa-miR-106a-5p	LPIN1	lipin 1
833	72	hsa-miR-106a-5p	PURA	purine rich element binding protein A
834	72	hsa-miR-106a-5p	PDPR	pyruvate dehydrogenase phosphatase regulatory subunit

835	72	hsa-miR-106a-5p	GGCX	gamma-glutamyl carboxylase
836	72	hsa-miR-106a-5p	DPYD	dihydropyrimidine dehydrogen-ase
837	72	hsa-miR-106a-5p	HP1BP3	heterochromatin protein 1 bind-ing protein 3
838	72	hsa-miR-106a-5p	FMNL3	formin like 3
839	72	hsa-miR-106a-5p	PCMTD1	protein-L-isoaspartate (D-aspar-tate) O-methyltransferase do-main containing 1
840	72	hsa-miR-106a-5p	LAMP3	lysosomal associated membrane protein 3
841	72	hsa-miR-106a-5p	MAP3K5	mitogen-activated protein ki-nase kinase kinase 5
842	71	hsa-miR-106a-5p	HMBOX1	homeobox containing 1
843	71	hsa-miR-106a-5p	TMEM131L	transmembrane 131 like
844	71	hsa-miR-106a-5p	CADM2	cell adhesion molecule 2
845	71	hsa-miR-106a-5p	RAB3B	RAB3B, member RAS onco-gene family
846	71	hsa-miR-106a-5p	MYO5C	myosin VC
847	71	hsa-miR-106a-5p	LIF	LIF, interleukin 6 family cyto-kine
848	71	hsa-miR-106a-5p	CSGAL-NACT1	chondroitin sulfate N-acetylga-lactosaminyltransferase 1
849	71	hsa-miR-106a-5p	RELL1	RELT like 1
850	71	hsa-miR-106a-5p	ITPKB	inositol-trisphosphate 3-kinase B
851	71	hsa-miR-106a-5p	SASH1	SAM and SH3 domain contain-ing 1
852	71	hsa-miR-106a-5p	ZC3H7B	zinc finger CCCH-type contain-ing 7B
853	71	hsa-miR-106a-5p	EGR3	early growth response 3
854	71	hsa-miR-106a-5p	BECN1	beclin 1
855	71	hsa-miR-106a-5p	EPGN	epithelial mitogen
856	71	hsa-miR-106a-5p	B2M	beta-2-microglobulin
857	71	hsa-miR-106a-5p	CLEC4D	C-type lectin domain family 4 member D
858	71	hsa-miR-106a-5p	TMEM9B	TMEM9 domain family mem-ber B

859	70	hsa-miR-106a-5p	MAP3K13	mitogen-activated protein kinase kinase kinase 13
860	70	hsa-miR-106a-5p	ZNF217	zinc finger protein 217
861	70	hsa-miR-106a-5p	TMCC3	transmembrane and coiled-coil domain family 3
862	70	hsa-miR-106a-5p	MVK	mevalonate kinase
863	70	hsa-miR-106a-5p	SNTG1	syntrophin gamma 1
864	70	hsa-miR-106a-5p	XIRP2	xin actin binding repeat containing 2
865	70	hsa-miR-106a-5p	BTN3A2	butyrophilin subfamily 3 member A2
866	70	hsa-miR-106a-5p	DCUN1D3	defective in cullin neddylation 1 domain containing 3
867	70	hsa-miR-106a-5p	KIRREL1	kirre like nephrin family adhesion molecule 1
868	70	hsa-miR-106a-5p	TIMP2	TIMP metallopeptidase inhibitor 2
869	70	hsa-miR-106a-5p	BUD23	BUD23, rRNA methyltransferase and ribosome maturation factor
870	70	hsa-miR-106a-5p	RNASEL	ribonuclease L
871	70	hsa-miR-106a-5p	LRRC20	leucine rich repeat containing 20
872	70	hsa-miR-106a-5p	RILPL1	Rab interacting lysosomal protein like 1
873	70	hsa-miR-106a-5p	CSDE1	cold shock domain containing E1
874	69	hsa-miR-106a-5p	TYW5	tRNA-yW synthesizing protein 5
875	69	hsa-miR-106a-5p	CHD9	chromodomain helicase DNA binding protein 9
876	69	hsa-miR-106a-5p	SMAD7	SMAD family member 7
877	69	hsa-miR-106a-5p	TBC1D2	TBC1 domain family member 2
878	69	hsa-miR-106a-5p	KCNQ2	potassium voltage-gated channel subfamily Q member 2
879	69	hsa-miR-106a-5p	CARNMT1	carnosine N-methyltransferase 1
880	69	hsa-miR-106a-5p	YPEL2	yippee like 2

881	69	hsa-miR-106a-5p	SOBP	sine oculis binding protein homolog
882	69	hsa-miR-106a-5p	INO80	INO80 complex subunit
883	69	hsa-miR-106a-5p	SNX21	sorting nexin family member 21
884	69	hsa-miR-106a-5p	SOGA1	suppressor of glucose, autophagy associated 1
885	69	hsa-miR-106a-5p	MUC17	mucin 17, cell surface associated
886	69	hsa-miR-106a-5p	GMCL1	germ cell-less, spermatogenesis associated 1
887	69	hsa-miR-106a-5p	HCN4	hyperpolarization activated cyclic nucleotide gated potassium channel 4
888	69	hsa-miR-106a-5p	THBS2	thrombospondin 2
889	69	hsa-miR-106a-5p	PLSCR4	phospholipid scramblase 4
890	69	hsa-miR-106a-5p	DGKH	diacylglycerol kinase eta
891	69	hsa-miR-106a-5p	ISM1	isthmin 1
892	68	hsa-miR-106a-5p	EFCAB1	EF-hand calcium binding domain 1
893	68	hsa-miR-106a-5p	SLC25A36	solute carrier family 25 member 36
894	68	hsa-miR-106a-5p	CDCA7	cell division cycle associated 7
895	68	hsa-miR-106a-5p	STK11IP	serine/threonine kinase 11 interacting protein
896	68	hsa-miR-106a-5p	BMP2	bone morphogenetic protein 2
897	68	hsa-miR-106a-5p	YWHAQ	tyrosine 3-monooxygenase/tryptophan 5-monooxygenase activation protein theta
898	68	hsa-miR-106a-5p	TSC22D2	TSC22 domain family member 2
899	68	hsa-miR-106a-5p	FURIN	furin, paired basic amino acid cleaving enzyme
900	68	hsa-miR-106a-5p	PUDP	pseudouridine 5'-phosphatase
901	68	hsa-miR-106a-5p	ACIN1	apoptotic chromatin condensation inducer 1
902	68	hsa-miR-106a-5p	KLHL36	kelch like family member 36

903	68	hsa-miR-106a-5p	STARD8	StAR related lipid transfer domain containing 8
904	68	hsa-miR-106a-5p	COIL	coilin
905	68	hsa-miR-106a-5p	KCNE4	potassium voltage-gated channel subfamily E regulatory subunit 4
906	68	hsa-miR-106a-5p	SLC12A3	solute carrier family 12 member 3
907	68	hsa-miR-106a-5p	COA5	cytochrome c oxidase assembly factor 5
908	68	hsa-miR-106a-5p	COL4A1	collagen type IV alpha 1 chain
909	68	hsa-miR-106a-5p	YPEL4	yippee like 4
910	68	hsa-miR-106a-5p	CYB561D1	cytochrome b561 family member D1
911	68	hsa-miR-106a-5p	ARHGAP35	Rho GTPase activating protein 35
912	68	hsa-miR-106a-5p	NEK9	NIMA related kinase 9
913	68	hsa-miR-106a-5p	SRPK1	SRSF protein kinase 1
914	68	hsa-miR-106a-5p	SRGAP3	SLIT-ROBO Rho GTPase activating protein 3
915	68	hsa-miR-106a-5p	GCA	grancalcin
916	68	hsa-miR-106a-5p	SFXN5	sideroflexin 5
917	68	hsa-miR-106a-5p	RSBN1	round spermatid basic protein 1
918	68	hsa-miR-106a-5p	CYP26B1	cytochrome P450 family 26 subfamily B member 1
919	67	hsa-miR-106a-5p	CCSER1	coiled-coil serine rich protein 1
920	67	hsa-miR-106a-5p	GLO1	glyoxalase I
921	67	hsa-miR-106a-5p	PEAK1	pseudopodium enriched atypical kinase 1
922	67	hsa-miR-106a-5p	WDCP	WD repeat and coiled coil containing
923	67	hsa-miR-106a-5p	RASSF2	Ras association domain family member 2
924	67	hsa-miR-106a-5p	GPAM	glycerol-3-phosphate acyltransferase, mitochondrial
925	67	hsa-miR-106a-5p	SPATA1	spermatogenesis associated 1

926	67	hsa-miR-106a-5p	RRP15	ribosomal RNA processing 15 homolog
927	67	hsa-miR-106a-5p	RAB11FIP4	RAB11 family interacting protein 4
928	67	hsa-miR-106a-5p	E2F3	E2F transcription factor 3
929	67	hsa-miR-106a-5p	CYBB	cytochrome b-245 beta chain
930	67	hsa-miR-106a-5p	FAM129B	family with sequence similarity 129 member B
931	67	hsa-miR-106a-5p	MCC	MCC, WNT signaling pathway regulator
932	67	hsa-miR-106a-5p	AP3D1	adaptor related protein complex 3 subunit delta 1
933	67	hsa-miR-106a-5p	DNM1L	dynamin 1 like
934	67	hsa-miR-106a-5p	SINHCAF	SIN3-HDAC complex associated factor
935	67	hsa-miR-106a-5p	ZRANB1	zinc finger RANBP2-type containing 1
936	66	hsa-miR-106a-5p	KIAA1147	KIAA1147
937	66	hsa-miR-106a-5p	SLC31A2	solute carrier family 31 member 2
938	66	hsa-miR-106a-5p	KCNJ8	potassium voltage-gated channel subfamily J member 8
939	66	hsa-miR-106a-5p	MPDZ	multiple PDZ domain crumbs cell polarity complex component
940	66	hsa-miR-106a-5p	IGF2BP1	insulin like growth factor 2 mRNA binding protein 1
941	66	hsa-miR-106a-5p	SIK1	salt inducible kinase 1
942	66	hsa-miR-106a-5p	OGA	O-GlcNAcase
943	66	hsa-miR-106a-5p	EPHB4	EPH receptor B4
944	66	hsa-miR-106a-5p	CEP128	centrosomal protein 128
945	66	hsa-miR-106a-5p	PRRG4	proline rich and Gla domain 4
946	66	hsa-miR-106a-5p	HYDIN	HYDIN, axonemal central pair apparatus protein
947	66	hsa-miR-106a-5p	SIK1B	salt inducible kinase 1B (putative)
948	66	hsa-miR-106a-5p	SYT16	synaptotagmin 16

949	66	hsa-miR-106a-5p	VPS53	VPS53, GARP complex subunit
950	66	hsa-miR-106a-5p	VEGFA	vascular endothelial growth factor A
951	66	hsa-miR-106a-5p	TCF7L1	transcription factor 7 like 1
952	66	hsa-miR-106a-5p	SLC6A9	solute carrier family 6 member 9
953	66	hsa-miR-106a-5p	WDR33	WD repeat domain 33
954	66	hsa-miR-106a-5p	PXDN	peroxidasin
955	66	hsa-miR-106a-5p	METAP1	methionyl aminopeptidase 1
956	66	hsa-miR-106a-5p	IFIT5	interferon induced protein with tetratricopeptide repeats 5
957	66	hsa-miR-106a-5p	NETO2	neuropilin and tolloid like 2
958	66	hsa-miR-106a-5p	SERPINB8	serpin family B member 8
959	66	hsa-miR-106a-5p	ASXL3	ASXL transcriptional regulator 3
960	66	hsa-miR-106a-5p	PPP6R3	protein phosphatase 6 regula-tory subunit 3
961	66	hsa-miR-106a-5p	ADGRD1	adhesion G protein-coupled re-ceptor D1
962	65	hsa-miR-106a-5p	NSL1	NSL1, MIS12 kinetochore complex component
963	65	hsa-miR-106a-5p	ZNF532	zinc finger protein 532
964	65	hsa-miR-106a-5p	TNFSF11	TNF superfamily member 11
965	65	hsa-miR-106a-5p	ZNF417	zinc finger protein 417
966	65	hsa-miR-106a-5p	CDH24	cadherin 24
967	65	hsa-miR-106a-5p	PKMYT1	protein kinase, membrane asso-ciated tyrosine/threonine 1
968	65	hsa-miR-106a-5p	SH3GLB1	SH3 domain containing GRB2 like, endophilin B1
969	65	hsa-miR-106a-5p	PANK3	pantothenate kinase 3
970	65	hsa-miR-106a-5p	TRIM8	tripartite motif containing 8
971	65	hsa-miR-106a-5p	GABRA1	gamma-aminobutyric acid type A receptor alpha1 subunit
972	65	hsa-miR-106a-5p	LUZP1	leucine zipper protein 1

973	65	hsa-miR-106a-5p	DMRTA2	DMRT like family A2
974	65	hsa-miR-106a-5p	NOL4	nucleolar protein 4
975	65	hsa-miR-106a-5p	DPY19L3	dpy-19 like C-mannosyltrans-ferase 3
976	65	hsa-miR-106a-5p	HESX1	HESX homeobox 1
977	65	hsa-miR-106a-5p	REL	REL proto-oncogene, NF-kB subunit
978	65	hsa-miR-106a-5p	TCEAL7	transcription elongation factor A like 7
979	65	hsa-miR-106a-5p	TBX3	T-box 3
980	65	hsa-miR-106a-5p	MYF5	myogenic factor 5
981	65	hsa-miR-106a-5p	SFR1	SWI5 dependent homologous recombination repair protein 1
982	65	hsa-miR-106a-5p	FGFR1OP	FGFR1 oncogene partner
983	65	hsa-miR-106a-5p	COA1	cytochrome c oxidase assembly factor 1 homolog
984	65	hsa-miR-106a-5p	CNTNAP3	contactin associated protein like 3
985	65	hsa-miR-106a-5p	TTC39C	tetratricopeptide repeat domain 39C
986	65	hsa-miR-106a-5p	CBFB	core-binding factor subunit beta
987	65	hsa-miR-106a-5p	PKNOX1	PBX/knotted 1 homeobox 1
988	65	hsa-miR-106a-5p	PPP2R2A	protein phosphatase 2 regula-tory subunit Balpha
989	64	hsa-miR-106a-5p	NDNF	neuron derived neurotrophic factor
990	64	hsa-miR-106a-5p	KMT2C	lysine methyltransferase 2C
991	64	hsa-miR-106a-5p	PTPRT	protein tyrosine phosphatase, receptor type T
992	64	hsa-miR-106a-5p	TTPAL	alpha tocopherol transfer pro-tein like
993	64	hsa-miR-106a-5p	RAD17	RAD17 checkpoint clamp loader component
994	64	hsa-miR-106a-5p	MID1	midline 1
995	64	hsa-miR-106a-5p	NFATC2IP	nuclear factor of activated T cells 2 interacting protein

996	64	hsa-miR-106a-5p	KPNA4	karyopherin subunit alpha 4
997	64	hsa-miR-106a-5p	GTPBP10	GTP binding protein 10
998	64	hsa-miR-106a-5p	PKIA	cAMP-dependent protein kinase inhibitor alpha
999	64	hsa-miR-106a-5p	RPF2	ribosome production factor 2 homolog
1000	64	hsa-miR-106a-5p	MAP3K1	mitogen-activated protein kinase kinase kinase 1
1001	64	hsa-miR-106a-5p	LHFPL2	LHFPL tetraspan subfamily member 2
1002	64	hsa-miR-106a-5p	SUSD1	sushi domain containing 1
1003	64	hsa-miR-106a-5p	CABLES1	Cdk5 and Abl enzyme substrate 1
1004	64	hsa-miR-106a-5p	GBP3	guanylate binding protein 3
1005	64	hsa-miR-106a-5p	TNRC6B	trinucleotide repeat containing 6B
1006	63	hsa-miR-106a-5p	ZNF445	zinc finger protein 445
1007	63	hsa-miR-106a-5p	ZDHHC21	zinc finger DHHC-type containing 21
1008	63	hsa-miR-106a-5p	CDC5L	cell division cycle 5 like
1009	63	hsa-miR-106a-5p	PRDM10	PR/SET domain 10
1010	63	hsa-miR-106a-5p	TNS1	tensin 1
1011	63	hsa-miR-106a-5p	BCR	BCR, RhoGEF and GTPase activating protein
1012	63	hsa-miR-106a-5p	AK4	adenylate kinase 4
1013	63	hsa-miR-106a-5p	TNFSF13B	TNF superfamily member 13b
1014	63	hsa-miR-106a-5p	PPP1R12B	protein phosphatase 1 regulatory subunit 12B
1015	63	hsa-miR-106a-5p	FIGNL1	fidgetin like 1
1016	63	hsa-miR-106a-5p	YTHDF3	YTH N6-methyladenosine RNA binding protein 3
1017	63	hsa-miR-106a-5p	UBR5	ubiquitin protein ligase E3 component n-recognin 5
1018	63	hsa-miR-106a-5p	CERS6	ceramide synthase 6
1019	63	hsa-miR-106a-5p	MECP2	methyl-CpG binding protein 2

1020	63	hsa-miR-106a-5p	CCL1	C-C motif chemokine ligand 1
1021	63	hsa-miR-106a-5p	CBX5	chromobox 5
1022	63	hsa-miR-106a-5p	CCND2	cyclin D2
1023	63	hsa-miR-106a-5p	ABCA10	ATP binding cassette subfamily A member 10
1024	63	hsa-miR-106a-5p	NR2C2AP	nuclear receptor 2C2 associated protein
1025	63	hsa-miR-106a-5p	RND3	Rho family GTPase 3
1026	63	hsa-miR-106a-5p	FRMD4A	FERM domain containing 4A
1027	63	hsa-miR-106a-5p	GOLPH3	golgi phosphoprotein 3
1028	62	hsa-miR-106a-5p	MED17	mediator complex subunit 17
1029	62	hsa-miR-106a-5p	SLC35F3	solute carrier family 35 member F3
1030	62	hsa-miR-106a-5p	AMPD3	adenosine monophosphate deaminase 3
1031	62	hsa-miR-106a-5p	FGF5	fibroblast growth factor 5
1032	62	hsa-miR-106a-5p	CLEC12A	C-type lectin domain family 12 member A
1033	62	hsa-miR-106a-5p	CCDC68	coiled-coil domain containing 68
1034	62	hsa-miR-106a-5p	TMTC1	transmembrane and tetratricopeptide repeat containing 1
1035	62	hsa-miR-106a-5p	CAMTA2	calmodulin binding transcription activator 2
1036	62	hsa-miR-106a-5p	DAZAP2	DAZ associated protein 2
1037	62	hsa-miR-106a-5p	YES1	YES proto-oncogene 1, Src family tyrosine kinase
1038	62	hsa-miR-106a-5p	ADAMTS5	ADAM metallopeptidase with thrombospondin type 1 motif 5
1039	62	hsa-miR-106a-5p	RIMBP2	RIMS binding protein 2
1040	62	hsa-miR-106a-5p	CHIC1	cysteine rich hydrophobic domain 1
1041	62	hsa-miR-106a-5p	TMEM123	transmembrane protein 123
1042	62	hsa-miR-106a-5p	SLC2A4	solute carrier family 2 member 4

1043	62	hsa-miR-106a-5p	HECA	hdc homolog, cell cycle regulator
1044	62	hsa-miR-106a-5p	SMAD1	SMAD family member 1
1045	62	hsa-miR-106a-5p	NFE2L1	nuclear factor, erythroid 2 like 1
1046	62	hsa-miR-106a-5p	TNRC6C	trinucleotide repeat containing 6C
1047	62	hsa-miR-106a-5p	IPMK	inositol polyphosphate multikinase
1048	62	hsa-miR-106a-5p	MRPL43	mitochondrial ribosomal protein L43
1049	62	hsa-miR-106a-5p	FAXC	failed axon connections homolog
1050	62	hsa-miR-106a-5p	RBMS1	RNA binding motif single stranded interacting protein 1
1051	62	hsa-miR-106a-5p	NRG3	neuregulin 3
1052	62	hsa-miR-106a-5p	MICOS10-NBL1	MICOS10-NBL1 readthrough
1053	61	hsa-miR-106a-5p	PDK3	pyruvate dehydrogenase kinase 3
1054	61	hsa-miR-106a-5p	CNTNAP3B	contactin associated protein like 3B
1055	61	hsa-miR-106a-5p	WNK1	WNK lysine deficient protein kinase 1
1056	61	hsa-miR-106a-5p	MRGPRX3	MAS related GPR family member X3
1057	61	hsa-miR-106a-5p	IKZF2	IKAROS family zinc finger 2
1058	61	hsa-miR-106a-5p	NPM1	nucleophosmin 1
1059	61	hsa-miR-106a-5p	SZT2	SZT2, KICSTOR complex subunit
1060	61	hsa-miR-106a-5p	SLC24A4	solute carrier family 24 member 4
1061	61	hsa-miR-106a-5p	FBXO11	F-box protein 11
1062	61	hsa-miR-106a-5p	LAMP5	lysosomal associated membrane protein family member 5
1063	61	hsa-miR-106a-5p	PBXIP1	PBX homeobox interacting protein 1
1064	61	hsa-miR-106a-5p	CPOX	coproporphyrinogen oxidase

1065	61	hsa-miR-106a-5p	IL17RD	interleukin 17 receptor D
1066	61	hsa-miR-106a-5p	NR2E3	nuclear receptor subfamily 2 group E member 3
1067	61	hsa-miR-106a-5p	FGD1	FYVE, RhoGEF and PH domain containing 1
1068	61	hsa-miR-106a-5p	COX7A2	cytochrome c oxidase subunit 7A2
1069	61	hsa-miR-106a-5p	INHBA	inhibin subunit beta A
1070	61	hsa-miR-106a-5p	EPB41L5	erythrocyte membrane protein band 4.1 like 5
1071	61	hsa-miR-106a-5p	BMP8B	bone morphogenetic protein 8b
1072	61	hsa-miR-106a-5p	CAPS2	calcyphosine 2
1073	61	hsa-miR-106a-5p	DGKE	diacylglycerol kinase epsilon
1074	61	hsa-miR-106a-5p	TBL1X	transducin beta like 1 X-linked
1075	61	hsa-miR-106a-5p	MKLN1	muskelin 1
1076	61	hsa-miR-106a-5p	JRKL	JRK like
1077	60	hsa-miR-106a-5p	WNT9B	Wnt family member 9B
1078	60	hsa-miR-106a-5p	MMAA	metabolism of cobalamin associated A
1079	60	hsa-miR-106a-5p	BBIP1	BBSome interacting protein 1
1080	60	hsa-miR-106a-5p	FER	FER tyrosine kinase
1081	60	hsa-miR-106a-5p	ARL1	ADP ribosylation factor like GTPase 1
1082	60	hsa-miR-106a-5p	FXR1	FMR1 autosomal homolog 1
1083	60	hsa-miR-106a-5p	FAM8A1	family with sequence similarity 8 member A1
1084	60	hsa-miR-106a-5p	PDZD11	PDZ domain containing 11
1085	60	hsa-miR-106a-5p	MXD1	MAX dimerization protein 1
1086	60	hsa-miR-106a-5p	FNIP2	folliculin interacting protein 2
1087	60	hsa-miR-106a-5p	RXFP1	relaxin family peptide receptor 1
1088	60	hsa-miR-106a-5p	IKBIP	IKBKB interacting protein
1089	60	hsa-miR-106a-5p	ZNF107	zinc finger protein 107
1090	60	hsa-miR-106a-5p	RAX	retina and anterior neural fold homeobox

1091	60	hsa-miR-106a-5p	C2CD4A	C2 calcium dependent domain containing 4A
1092	60	hsa-miR-106a-5p	PPM1A	protein phosphatase, Mg2+/Mn2+ dependent 1A
1093	60	hsa-miR-106a-5p	NTRK2	neurotrophic receptor tyrosine kinase 2
1094	60	hsa-miR-106a-5p	UGCG	UDP-glucose ceramide glucosyltransferase
1095	60	hsa-miR-106a-5p	POGK	pogo transposable element derived with KRAB domain
1096	60	hsa-miR-106a-5p	GALNT10	polypeptide N-acetylgalactosaminyltransferase 10
1097	60	hsa-miR-106a-5p	ZFYVE21	zinc finger FYVE-type containing 21
1098	60	hsa-miR-106a-5p	MINDY2	MINDY lysine 48 deubiquitinase 2
1099	60	hsa-miR-106a-5p	NUFIP2	nuclear FMR1 interacting protein 2
1100	60	hsa-miR-106a-5p	AJUBA	ajuba LIM protein
1101	60	hsa-miR-106a-5p	RGS4	regulator of G protein signaling 4
1102	60	hsa-miR-106a-5p	VEZF1	vascular endothelial zinc finger 1
1103	59	hsa-miR-106a-5p	GDF11	growth differentiation factor 11
1104	59	hsa-miR-106a-5p	FKBP5	FKBP prolyl isomerase 5
1105	59	hsa-miR-106a-5p	DEDD	death effector domain containing
1106	59	hsa-miR-106a-5p	NAV2	neuron navigator 2
1107	59	hsa-miR-106a-5p	YTHDC1	YTH domain containing 1
1108	59	hsa-miR-106a-5p	TIGAR	TP53 induced glycolysis regulatory phosphatase
1109	59	hsa-miR-106a-5p	COQ2	coenzyme Q2, polyprenyltransferase
1110	59	hsa-miR-106a-5p	HDAC4	histone deacetylase 4
1111	59	hsa-miR-106a-5p	PIP4K2A	phosphatidylinositol-5-phosphate 4-kinase type 2 alpha
1112	59	hsa-miR-106a-5p	ARHGAP24	Rho GTPase activating protein 24

1113	59	hsa-miR-106a-5p	EDA2R	ectodysplasin A2 receptor
1114	59	hsa-miR-106a-5p	MS4A14	membrane spanning 4-domains A14
1115	59	hsa-miR-106a-5p	DIP2A	disco interacting protein 2 homolog A
1116	59	hsa-miR-106a-5p	FANCD2	FA complementation group D2
1117	59	hsa-miR-106a-5p	AP1G1	adaptor related protein complex 1 subunit gamma 1
1118	59	hsa-miR-106a-5p	C3orf35	chromosome 3 open reading frame 35
1119	59	hsa-miR-106a-5p	SULF1	sulfatase 1
1120	59	hsa-miR-106a-5p	TBCEL	tubulin folding cofactor E like
1121	59	hsa-miR-106a-5p	FOXA1	forkhead box A1
1122	59	hsa-miR-106a-5p	MXI1	MAX interactor 1, dimerization protein
1123	58	hsa-miR-106a-5p	MYCN	MYCN proto-oncogene, bHLH transcription factor
1124	58	hsa-miR-106a-5p	AVL9	AVL9 cell migration associated
1125	58	hsa-miR-106a-5p	COL19A1	collagen type XIX alpha 1 chain
1126	58	hsa-miR-106a-5p	KBTBD8	kelch repeat and BTB domain containing 8
1127	58	hsa-miR-106a-5p	SLC11A1	solute carrier family 11 member 1
1128	58	hsa-miR-106a-5p	SIRPA	signal regulatory protein alpha
1129	58	hsa-miR-106a-5p	SDC2	syndecan 2
1130	58	hsa-miR-106a-5p	SART1	spliceosome associated factor 1, recruiter of U4/U6.U5 tri-snRNP
1131	58	hsa-miR-106a-5p	FGF4	fibroblast growth factor 4
1132	58	hsa-miR-106a-5p	ADGRL3	adhesion G protein-coupled receptor L3
1133	58	hsa-miR-106a-5p	TMEM242	transmembrane protein 242
1134	58	hsa-miR-106a-5p	NRSN1	neurensin 1
1135	58	hsa-miR-106a-5p	NFE2L2	nuclear factor, erythroid 2 like 2

1136	58	hsa-miR-106a-5p	SKI	SKI proto-oncogene
1137	58	hsa-miR-106a-5p	EIF4E2	eukaryotic translation initiation factor 4E family member 2
1138	58	hsa-miR-106a-5p	PHF1	PHD finger protein 1
1139	58	hsa-miR-106a-5p	UBASH3B	ubiquitin associated and SH3 domain containing B
1140	58	hsa-miR-106a-5p	CBX1	chromobox 1
1141	58	hsa-miR-106a-5p	SEMA5A	semaphorin 5A
1142	58	hsa-miR-106a-5p	TLE4	TLE family member 4, tran-scriptional corepressor
1143	58	hsa-miR-106a-5p	AREL1	apoptosis resistant E3 ubiquitin protein ligase 1
1144	58	hsa-miR-106a-5p	ERBB3	erb-b2 receptor tyrosine kinase 3
1145	58	hsa-miR-106a-5p	CDC25A	cell division cycle 25A
1146	58	hsa-miR-106a-5p	TNC	tenascin C
1147	58	hsa-miR-106a-5p	RANBP6	RAN binding protein 6
1148	58	hsa-miR-106a-5p	CYP2U1	cytochrome P450 family 2 sub-family U member 1
1149	57	hsa-miR-106a-5p	SLC36A1	solute carrier family 36 member 1
1150	57	hsa-miR-106a-5p	KIAA1671	KIAA1671
1151	57	hsa-miR-106a-5p	FAM227A	family with sequence similarity 227 member A
1152	57	hsa-miR-106a-5p	FNDC3A	fibronectin type III domain con-taining 3A
1153	57	hsa-miR-106a-5p	AFF4	AF4/FMR2 family member 4
1154	57	hsa-miR-106a-5p	ZFP28	ZFP28 zinc finger protein
1155	57	hsa-miR-106a-5p	PRND	prion like protein doppel
1156	57	hsa-miR-106a-5p	TMUB2	transmembrane and ubiquitin like domain containing 2
1157	57	hsa-miR-106a-5p	CARD8	caspase recruitment domain family member 8
1158	57	hsa-miR-106a-5p	STC1	stanniocalcin 1
1159	57	hsa-miR-106a-5p	ELOA	elongin A

1160	57	hsa-miR-106a-5p	XRRA1	X-ray radiation resistance associated 1
1161	57	hsa-miR-106a-5p	AGA	aspartylglucosaminidase
1162	57	hsa-miR-106a-5p	SLC5A3	solute carrier family 5 member 3
1163	57	hsa-miR-106a-5p	HIBCH	3-hydroxyisobutyryl-CoA hydrolase
1164	57	hsa-miR-106a-5p	DLC1	DLC1 Rho GTPase activating protein
1165	57	hsa-miR-106a-5p	PPP1R1C	protein phosphatase 1 regulatory inhibitor subunit 1C
1166	57	hsa-miR-106a-5p	ZNF780B	zinc finger protein 780B
1167	57	hsa-miR-106a-5p	VPS26A	VPS26, retromer complex component A
1168	57	hsa-miR-106a-5p	CERS2	ceramide synthase 2
1169	57	hsa-miR-106a-5p	ACTR1A	ARP1 actin related protein 1 homolog A
1170	57	hsa-miR-106a-5p	CYP20A1	cytochrome P450 family 20 subfamily A member 1
1171	56	hsa-miR-106a-5p	NAA50	N(alpha)-acetyltransferase 50, NatE catalytic subunit
1172	56	hsa-miR-106a-5p	DPH6	diphthamine biosynthesis 6
1173	56	hsa-miR-106a-5p	KIF13A	kinesin family member 13A
1174	56	hsa-miR-106a-5p	DCTN6	dynactin subunit 6
1175	56	hsa-miR-106a-5p	PDGFRB	platelet derived growth factor receptor beta
1176	56	hsa-miR-106a-5p	SPSB4	splA/ryanodine receptor domain and SOCS box containing 4
1177	56	hsa-miR-106a-5p	GIT2	GIT ArfGAP 2
1178	56	hsa-miR-106a-5p	SLC25A27	solute carrier family 25 member 27
1179	56	hsa-miR-106a-5p	DHRS12	dehydrogenase/reductase 12
1180	56	hsa-miR-106a-5p	SLC39A6	solute carrier family 39 member 6
1181	56	hsa-miR-106a-5p	PNPLA4	patatin like phospholipase domain containing 4

1182	56	hsa-miR-106a-5p	EIF4H	eukaryotic translation initiation factor 4H
1183	56	hsa-miR-106a-5p	C15orf41	chromosome 15 open reading frame 41
1184	56	hsa-miR-106a-5p	ZNF510	zinc finger protein 510
1185	56	hsa-miR-106a-5p	PCDHA9	protocadherin alpha 9
1186	56	hsa-miR-106a-5p	FIBIN	fin bud initiation factor homolog
1187	56	hsa-miR-106a-5p	SCN3A	sodium voltage-gated channel alpha subunit 3
1188	56	hsa-miR-106a-5p	COL4A2	collagen type IV alpha 2 chain
1189	56	hsa-miR-106a-5p	TANC2	tetratricopeptide repeat, ankyrin repeat and coiled-coil containing 2
1190	56	hsa-miR-106a-5p	GK5	glycerol kinase 5
1191	56	hsa-miR-106a-5p	ENTPD7	ectonucleoside triphosphate diphosphohydrolase 7
1192	56	hsa-miR-106a-5p	CNN1	calponin 1
1193	56	hsa-miR-106a-5p	AIFM2	apoptosis inducing factor, mitochondria associated 2
1194	56	hsa-miR-106a-5p	FOXL2	forkhead box L2
1195	56	hsa-miR-106a-5p	FNBP4	formin binding protein 4
1196	55	hsa-miR-106a-5p	PSD3	pleckstrin and Sec7 domain containing 3
1197	55	hsa-miR-106a-5p	ENSA	endosulfine alpha
1198	55	hsa-miR-106a-5p	MCTP2	multiple C2 and transmembrane domain containing 2
1199	55	hsa-miR-106a-5p	KLK7	kallikrein related peptidase 7
1200	55	hsa-miR-106a-5p	ACADSB	acyl-CoA dehydrogenase short/branched chain
1201	55	hsa-miR-106a-5p	PAX6	paired box 6
1202	55	hsa-miR-106a-5p	KIF14	kinesin family member 14
1203	55	hsa-miR-106a-5p	OR2W5	olfactory receptor family 2 subfamily W member 5 (gene/pseudogene)
1204	55	hsa-miR-106a-5p	PAM	peptidylglycine alpha-amidating monooxygenase

1205	55	hsa-miR-106a-5p	HARS	histidyl-tRNA synthetase
1206	55	hsa-miR-106a-5p	RCAN3	RCAN family member 3
1207	55	hsa-miR-106a-5p	CEP126	centrosomal protein 126
1208	55	hsa-miR-106a-5p	RUBCN	rubicon autophagy regulator
1209	55	hsa-miR-106a-5p	SMAD6	SMAD family member 6
1210	55	hsa-miR-106a-5p	TMCC1	transmembrane and coiled-coil domain family 1
1211	55	hsa-miR-106a-5p	RBM41	RNA binding motif protein 41
1212	55	hsa-miR-106a-5p	HMGB2	high mobility group box 2
1213	55	hsa-miR-106a-5p	TCF4	transcription factor 4
1214	55	hsa-miR-106a-5p	EXPH5	exophilin 5
1215	55	hsa-miR-106a-5p	SNX12	sorting nexin 12
1216	55	hsa-miR-106a-5p	RPS6KA2	ribosomal protein S6 kinase A2
1217	55	hsa-miR-106a-5p	FBXO28	F-box protein 28
1218	55	hsa-miR-106a-5p	NTRK3	neurotrophic receptor tyrosine kinase 3
1219	55	hsa-miR-106a-5p	VDAC1	voltage dependent anion channel 1
1220	55	hsa-miR-106a-5p	CAMK2D	calcium/calmodulin dependent protein kinase II delta
1221	55	hsa-miR-106a-5p	IPO7	importin 7
1222	55	hsa-miR-106a-5p	RNF24	ring finger protein 24
1223	55	hsa-miR-106a-5p	UBR1	ubiquitin protein ligase E3 component n-recognin 1
1224	55	hsa-miR-106a-5p	CDC40	cell division cycle 40
1225	55	hsa-miR-106a-5p	ADAMTSL5	ADAMTS like 5
1226	55	hsa-miR-106a-5p	PTPRO	protein tyrosine phosphatase, receptor type O
1227	55	hsa-miR-106a-5p	LIAS	lipoic acid synthetase
1228	55	hsa-miR-106a-5p	SRSF2	serine and arginine rich splicing factor 2
1229	55	hsa-miR-106a-5p	SETD2	SET domain containing 2, histone lysine methyltransferase
1230	55	hsa-miR-106a-5p	FBXL22	F-box and leucine rich repeat protein 22

1231	55	hsa-miR-106a-5p	RARB	retinoic acid receptor beta
1232	55	hsa-miR-106a-5p	RPGR	retinitis pigmentosa GTPase regulator
1233	55	hsa-miR-106a-5p	ATE1	arginyltransferase 1
1234	55	hsa-miR-106a-5p	MAVS	mitochondrial antiviral signaling protein
1235	54	hsa-miR-106a-5p	RCOR1	REST corepressor 1
1236	54	hsa-miR-106a-5p	C16orf72	chromosome 16 open reading frame 72
1237	54	hsa-miR-106a-5p	C6orf141	chromosome 6 open reading frame 141
1238	54	hsa-miR-106a-5p	MAST3	microtubule associated serine/threonine kinase 3
1239	54	hsa-miR-106a-5p	GJA1	gap junction protein alpha 1
1240	54	hsa-miR-106a-5p	BCL2L15	BCL2 like 15
1241	54	hsa-miR-106a-5p	PCBP2	poly(rC) binding protein 2
1242	54	hsa-miR-106a-5p	CD36	CD36 molecule
1243	54	hsa-miR-106a-5p	INTS6	integrator complex subunit 6
1244	54	hsa-miR-106a-5p	SH3TC2	SH3 domain and tetratricopeptide repeats 2
1245	54	hsa-miR-106a-5p	RMDN3	regulator of microtubule dynamics 3
1246	54	hsa-miR-106a-5p	F2RL3	F2R like thrombin or trypsin receptor 3
1247	54	hsa-miR-106a-5p	MRPL19	mitochondrial ribosomal protein L19
1248	54	hsa-miR-106a-5p	DCUN1D5	defective in cullin neddylation 1 domain containing 5
1249	54	hsa-miR-106a-5p	UHRF1BP1	UHRF1 binding protein 1
1250	54	hsa-miR-106a-5p	MCM3	minichromosome maintenance complex component 3
1251	54	hsa-miR-106a-5p	TRAPPC10	trafficking protein particle complex 10
1252	54	hsa-miR-106a-5p	PPP2R3A	protein phosphatase 2 regulatory subunit B"alpha

1253	54	hsa-miR-106a-5p	ENTPD5	ectonucleoside triphosphate diphosphohydrolase 5
1254	54	hsa-miR-106a-5p	PBLD	phenazine biosynthesis like protein domain containing
1255	54	hsa-miR-106a-5p	UBE2W	ubiquitin conjugating enzyme E2 W
1256	54	hsa-miR-106a-5p	DDX46	DEAD-box helicase 46
1257	54	hsa-miR-106a-5p	SLITRK4	SLIT and NTRK like family member 4
1258	54	hsa-miR-106a-5p	IQSEC1	IQ motif and Sec7 domain 1
1259	54	hsa-miR-106a-5p	WDR36	WD repeat domain 36
1260	54	hsa-miR-106a-5p	AHRR	aryl-hydrocarbon receptor repressor
1261	53	hsa-miR-106a-5p	MICU3	mitochondrial calcium uptake family member 3
1262	53	hsa-miR-106a-5p	KRT23	keratin 23
1263	53	hsa-miR-106a-5p	PLEKHM3	pleckstrin homology domain containing M3
1264	53	hsa-miR-106a-5p	ELAVL2	ELAV like RNA binding protein 2
1265	53	hsa-miR-106a-5p	TMEM132B	transmembrane protein 132B
1266	53	hsa-miR-106a-5p	F2RL2	coagulation factor II thrombin receptor like 2
1267	53	hsa-miR-106a-5p	MMP3	matrix metallopeptidase 3
1268	53	hsa-miR-106a-5p	UBASH3A	ubiquitin associated and SH3 domain containing A
1269	53	hsa-miR-106a-5p	MTA3	metastasis associated 1 family member 3
1270	53	hsa-miR-106a-5p	PLPPR5	phospholipid phosphatase related 5
1271	53	hsa-miR-106a-5p	CACNB4	calcium voltage-gated channel auxiliary subunit beta 4
1272	53	hsa-miR-106a-5p	MAP3K3	mitogen-activated protein kinase kinase kinase 3
1273	53	hsa-miR-106a-5p	SNX9	sorting nexin 9
1274	53	hsa-miR-106a-5p	BTBD9	BTB domain containing 9

1275	53	hsa-miR-106a-5p	CCP110	centriolar coiled-coil protein 110
1276	53	hsa-miR-106a-5p	FOXJ2	forkhead box J2
1277	53	hsa-miR-106a-5p	EAF1	ELL associated factor 1
1278	53	hsa-miR-106a-5p	SP8	Sp8 transcription factor
1279	52	hsa-miR-106a-5p	NOTCH2NLA	notch 2 N-terminal like A
1280	52	hsa-miR-106a-5p	PRR11	proline rich 11
1281	52	hsa-miR-106a-5p	MYCT1	MYC target 1
1282	52	hsa-miR-106a-5p	MYCL	MYCL proto-oncogene, bHLH transcription factor
1283	52	hsa-miR-106a-5p	AFF1	AF4/FMR2 family member 1
1284	52	hsa-miR-106a-5p	ARSE	arylsulfatase E
1285	52	hsa-miR-106a-5p	DSTYK	dual serine/threonine and tyro-sine protein kinase
1286	52	hsa-miR-106a-5p	CTDSPL2	CTD small phosphatase like 2
1287	52	hsa-miR-106a-5p	GARS	glycyl-tRNA synthetase
1288	52	hsa-miR-106a-5p	SIRT5	sirtuin 5
1289	52	hsa-miR-106a-5p	PPARD	peroxisome proliferator activa-ted receptor delta
1290	52	hsa-miR-106a-5p	SYT10	synaptotagmin 10
1291	52	hsa-miR-106a-5p	PTGS1	prostaglandin-endoperoxide synthase 1
1292	52	hsa-miR-106a-5p	MICA	MHC class I polypeptide-re-lated sequence A
1293	52	hsa-miR-106a-5p	FAF2	Fas associated factor family member 2
1294	52	hsa-miR-106a-5p	CAMK2N1	calcium/calmodulin dependent protein kinase II inhibitor 1
1295	52	hsa-miR-106a-5p	WDR64	WD repeat domain 64
1296	52	hsa-miR-106a-5p	FAM168A	family with sequence similarity 168 member A
1297	52	hsa-miR-106a-5p	GKAP1	G kinase anchoring protein 1
1298	52	hsa-miR-106a-5p	USP53	ubiquitin specific peptidase 53
1299	51	hsa-miR-106a-5p	PLEKHM1	pleckstrin homology and RUN domain containing M1

1300	51	hsa-miR-106a-5p	AEN	apoptosis enhancing nuclease
1301	51	hsa-miR-106a-5p	SEC22C	SEC22 homolog C, vesicle trafficking protein
1302	51	hsa-miR-106a-5p	RNF145	ring finger protein 145
1303	51	hsa-miR-106a-5p	RASL12	RAS like family 12
1304	51	hsa-miR-106a-5p	C11orf58	chromosome 11 open reading frame 58
1305	51	hsa-miR-106a-5p	SLC35E2A	solute carrier family 35 member E2A
1306	51	hsa-miR-106a-5p	ASB1	ankyrin repeat and SOCS box containing 1
1307	51	hsa-miR-106a-5p	C21orf58	chromosome 21 open reading frame 58
1308	51	hsa-miR-106a-5p	UBAP1	ubiquitin associated protein 1
1309	51	hsa-miR-106a-5p	NCEH1	neutral cholesterol ester hydrolase 1
1310	51	hsa-miR-106a-5p	IPCEF1	interaction protein for cytohesin exchange factors 1
1311	51	hsa-miR-106a-5p	LTV1	LTV1 ribosome biogenesis factor
1312	51	hsa-miR-106a-5p	C9orf72	chromosome 9 open reading frame 72
1313	51	hsa-miR-106a-5p	MYO10	myosin X
1314	51	hsa-miR-106a-5p	SLCO1C1	solute carrier organic anion transporter family member 1C1
1315	51	hsa-miR-106a-5p	ZDHHC8	zinc finger DHHC-type containing 8
1316	51	hsa-miR-106a-5p	SPACA4	sperm acrosome associated 4
1317	51	hsa-miR-106a-5p	NXF1	nuclear RNA export factor 1
1318	51	hsa-miR-106a-5p	GPR26	G protein-coupled receptor 26
1319	51	hsa-miR-106a-5p	TTR	transthyretin
1320	51	hsa-miR-106a-5p	XIAP	X-linked inhibitor of apoptosis
1321	51	hsa-miR-106a-5p	SMIM13	small integral membrane protein 13
1322	51	hsa-miR-106a-5p	MBD5	methyl-CpG binding domain protein 5

1323	51	hsa-miR-106a-5p	TSKU	tsukushi, small leucine rich proteoglycan
1324	51	hsa-miR-106a-5p	FGF12	fibroblast growth factor 12
1325	50	hsa-miR-106a-5p	MOG	myelin oligodendrocyte glycoprotein
1326	50	hsa-miR-106a-5p	MARVELD3	MARVEL domain containing 3
1327	50	hsa-miR-106a-5p	SCD	stearoyl-CoA desaturase
1328	50	hsa-miR-106a-5p	MYO19	myosin XIX
1329	50	hsa-miR-106a-5p	KLF3	Kruppel like factor 3
1330	50	hsa-miR-106a-5p	MRTFB	myocardin related transcription factor B
1331	50	hsa-miR-106a-5p	OTUD1	OTU deubiquitinase 1
1332	50	hsa-miR-106a-5p	ZNF385A	zinc finger protein 385A
1333	50	hsa-miR-106a-5p	CNGB3	cyclic nucleotide gated channel beta 3
1334	50	hsa-miR-106a-5p	ABT1	activator of basal transcription 1
1335	50	hsa-miR-106a-5p	LPP	LIM domain containing preferred translocation partner in lipoma
1336	50	hsa-miR-106a-5p	TMED5	transmembrane p24 trafficking protein 5
1337	50	hsa-miR-106a-5p	MSMO1	methylsterol monooxygenase 1

Table S2. miR-106a, its regulation alterations, and its target genes by condition type (cancers, diseases, spermatogenesis, and aging).

Condition type		miR-106a regulation	Target genes	Reference
Cancers	Colorectal cancer	up	TGFBR2	18, 19
			ATG7	14
			PTEN/PI3K/AKT pathway	20
		down	E2F1	24
			caspase-9	24
	Cholangiocarcinoma	-	-	-
	Ewing Sarcoma	up	Bim	29
	Gastric cancer	up	TIMP2	34
			caspase-8	37
			PARP	37
			caspase-3	37
			FAS	38
			lncRNA-FER1L4	40, 41
			PTEN	41,42
			P-glycoprotein (P-gp)	42
			RUNX3	42, 43
	Esophageal Carcinoma	up	-	46
	Renal cell carcinoma	down	IRS-2	50
			PAK5	49
			VEGFA	57, 58
	Non-small cell lung cancer	down	-	60
		up	PTEN	61
			ABCA1	64
	Pancreatic cancer	up	TIMP-2	66
			RB1	66
	Ovarian cancer	up	PTEN	67
			ARHGAP24	67
			STAT3	68
			p130 (RBL2)	69
			Mcl-1	70
			BCL10	71
			caspase-7	71
			PDCD4	73
			lncRNA-XIST	76
	Brain tumors	up	WNT signaling pathway	79
			TIMP-2	80
			MAPK pathway	85

			NF-κB pathway	85
			MYCN	86
		down	E2F1	83
			SLC2A3	82
			FASTK	84
	Breast cancer	up	ZBTB4a	91
			PTEN	89
			DAX-1	90
			RUNX	89
	Endometrial cancer	up	BCL2L11	96
	Cervical cancer	up	TIMP-2	102
			LKB1	101
	Hepatocellular carcinoma	up	TIMP2	104
			TP53INP1	104
			CDKN1A	104
			FBXW7	109
			IncTCL6	103
			PTPN12	110
		down	Twist1	108
			FER1L4	105
			E2F1	105
	Melanoma	up	Cx43	111
		down	E2F3	116
			lncRNA H19	116
	Osteosarcoma	up	VNN2	117
			lncRNA HOTAIR	118
			STAT3	118
Non-cancer Conditions	Hepatitis B	down	IL-8	134
	Multiple sclerosis	down	RBL2	136
			APP	136
			CYP19A1	136
			BMP2	136
	Myasthenia gravis	down	-	142
	Cardiac hypertrophy	up	Mfn2	144
	Spermatogenesis	up	STAT3	152
			Ccnd1	152
	Aging	down	p21/CDKN1A	153, 158
